# U2 snRNP recognizes the branch site through a loaded-spring strand-invasion mechanism

**DOI:** 10.1093/nar/gkag429

**Published:** 2026-05-13

**Authors:** Pavlína Pokorná, Vladimir Pena, Alessandra Magistrato

**Affiliations:** CNR - Istituto Officina dei Materiali (IOM) at International School for Advanced Studies (SISSA), via Bonomea 265, 34136, Trieste, Italy; The Institute of Cancer Research, 123 Old Brompton Road, SW7 3RP, London, United Kingdom; CNR - Istituto Officina dei Materiali (IOM) at International School for Advanced Studies (SISSA), via Bonomea 265, 34136, Trieste, Italy

## Abstract

Recognition of the branch sequence (BS) by the U2 snRNP is a pivotal step in pre-mRNA splicing and spliceosome assembly. Structural studies suggest that BS recognition occurs through a toehold-mediated strand-invasion mechanism, in which U2 snRNA progressively base-pairs with the intron to form the branch helix. However, given the limited complementarity between U2 snRNA and the intronic BS, it remains unclear how spontaneous strand invasion can occur. Here, using all-atom and coarse-grained molecular dynamics simulations, we show that strand invasion proceeds spontaneously once the toehold region is engaged and the TAT-SF1 factor is released. The key finding is that the branch-stem loop (BSL) of the U2 snRNA is maintained in a supercoiled, high-energy conformation by TAT-SF1, which acts as a molecular latch holding the BSL in a poised “loaded-spring” state. Displacement of TAT-SF1 allows the BSL to relax, releasing the stored conformational energy that drives strand invasion through local strand-slip and base-pair exchange. Moreover, the simulations reveal that strand invasion can proceed bidirectionally, refining previous models of U2–BS pairing. This work establishes a “loaded-spring” mechanism as a key physical driver of the toehold-mediated strand invasion underlying branch-site recognition within the early spliceosome.

## Graphical Abstract

**Graphical Abstract:**
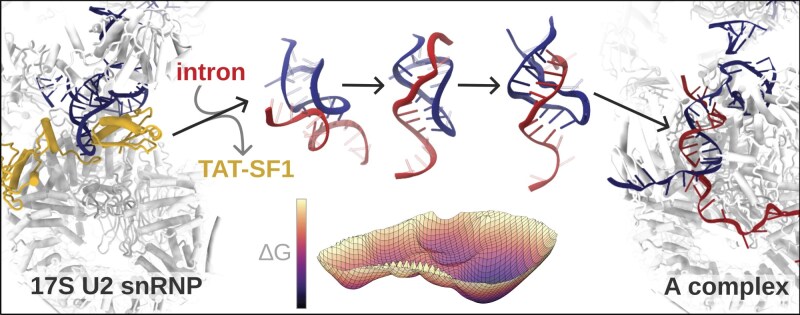

## Introduction

Strand invasion, also known as strand displacement, occurs when a single-stranded RNA or DNA strand invades a duplex, displacing one of its strands to hybridize with the other. This process occurs without external protein drivers, such as helicases [1]. *In vitro*, strand invasion processes find applications in nanotechnology [2, 3]. *In vivo*, this mechanism is suggested to mediate nucleic acid rearrangements and recognition, and is implicated in fundamental biological processes such as transcription regulation, protein synthesis, and pre-mRNA splicing [1].

Here we aimed to resolve the molecular mechanism of strand displacement in the context of pre-mRNA splicing. Splicing consists of removing non-coding regions (introns) and ligating the coding regions (exons) to produce functional protein-coding RNA and long non-coding RNAs [4, 5]. Splicing is orchestrated by the spliceosome, a large and dynamic ribonucleoprotein (RNP) engine [6–10]. Splicing fidelity, critical for maintaining proteome integrity, relies on the accurate recognition of specific pre-mRNA sequences [11]. These are the exon-intron boundaries (the 5′- and 3′-splice sites) and the branch sequence (BS) [12, 13]. The BS contains the branch point adenosine (BPA), which later initiates the first catalytic step of the splicing reaction [14, 15]. During spliceosome assembly, the BS is initially bound by the SF1 protein and is subsequently handed over to the U2 small nuclear (sn)RNP, where it pairs with the U2 snRNA and contacts the SF3b complex [16, 17].

**Figure 1.**
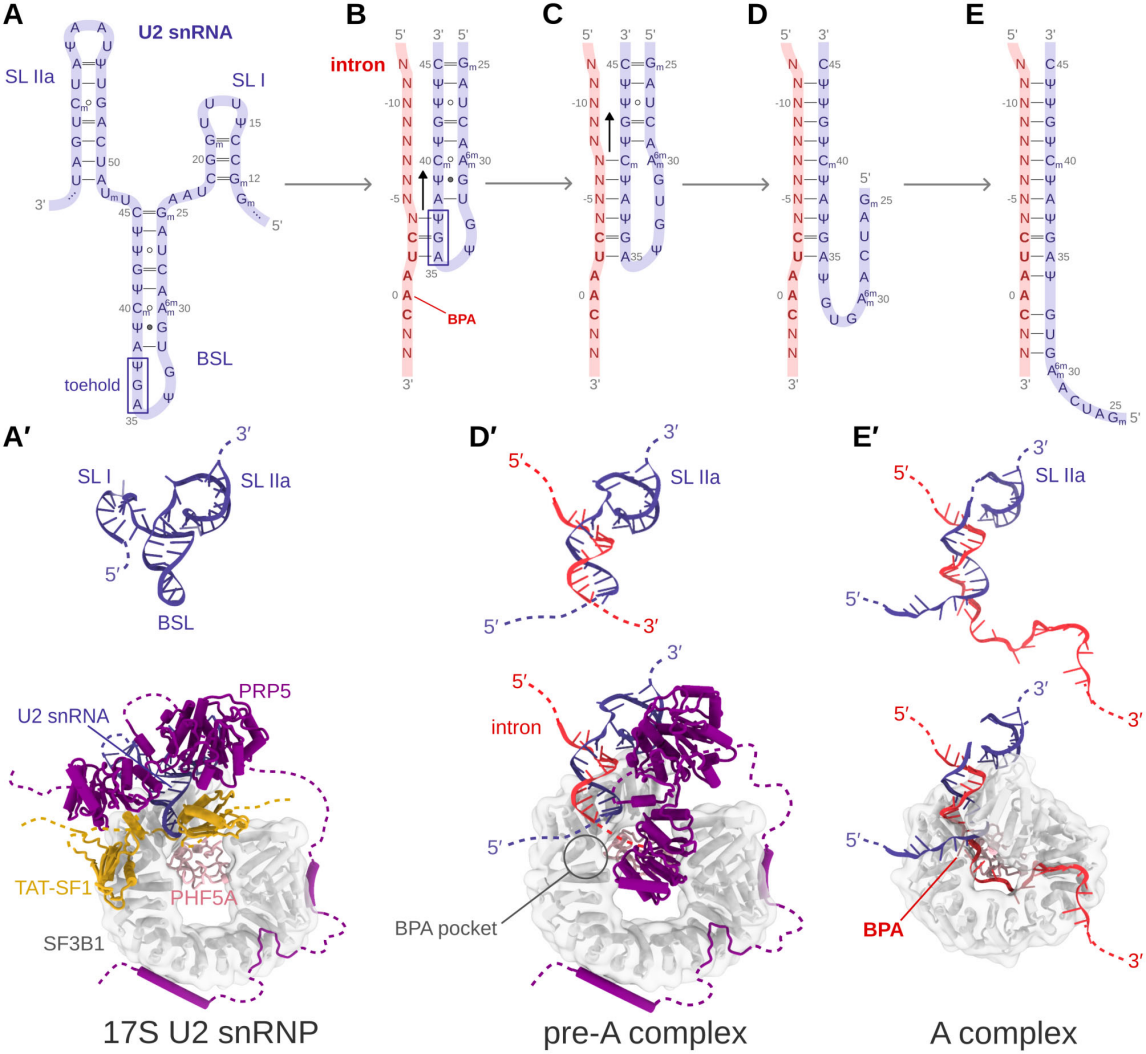
Strand displacement mechanism as proposed by Cretu *et al*. [18]. (**A**) Scheme of 3′ terminal portion of U2 snRNA. For clarity, stem loops SL I and SL IIa are omitted in panels (B−E). (**B**) Hypothesized formation of the initial toehold between the intron and the BSL of U2 snRNA. The yeast consensus BS sequence 5′-CUAAC-3′ is marked with bold red letters. The corresponding human consensus motif is 5′-YUNAY-3′, where A is the BPA, Y stands for pyrimidine and N for any base [4, 27, 28]. (**C**) Hypothesized growth mechanism of the branch helix. The 5′-part of the intron establishes additional base pair interactions with the BSL 3′-end. Notably, the intermediates depicted in panels (B) and (C) were not captured experimentally. (**D**) The invasion of the intron strand continues with the formation of the branch helix, while the 5′-end of the BSL dissociates. (**E**) Complete formation of the branch helix. (**A**′), (**D**′), and (**E**′) show RNA 3D structures corresponding those sketched in (A), (D), and (E). These 3D structures are cut from cryo-EM structures of the states captured in PDBs 7EVO [17], 7VPX [17], and 6FF4 [20]), respectively. The corresponding models, embedded in the SF3b complex, are shown in the bottom.

Based on cryo-EM studies, Cretu *et al*. have proposed that the branch-site recognition by the U2 snRNP occurs stepwise, through intron-mediated strand displacement [18]. Initially, upon binding to the U2 snRNA/SF3b complex, the intron BS hybridizes with a branch stem-loop (BSL) of the U2 snRNA (Fig. 1) [19]. Then, the pairing extends by forming a full branch helix, where the BPA is bulged out [16, 20–23].

In more detail, cryo-EM structures of the 17S U2 snRNP showed that three bases are extruded from the loop when the BSL binds to the SF3B1 protein, the main component of the SF3b complex. The rest of the BSL is instead masked by the TAT-SF1 (HTATSF1) protein (Fig. 1A). Subsequent TAT-SF1 removal and intron binding to the BSL is promoted by the PRP5 (also known as DDX46) ATPase/helicase, which also exerts intron proofreading [16, 19, 24–26]. The cryo-EM structure of a spliceosome, stalled with the splicing inhibitor spliceostatin A, has revealed an intermediate conformation, found halfway through branch duplex formation. This is a pre-A spliceosome state captured after PRP5 action, where the U2 snRNA has paired with the upstream (5′ direction) sequence of the BS to form a precursor of the branch helix (Fig. 1D) [17, 18]. Finally, BS recognition ends with the complete formation of the branch helix, which is embraced by the SF3B1 HEAT-like solenoid structure, and with the BPA docking into a binding pocket at the interface of the SF3B1/PHF5A proteins (Fig. 1E). The proposed branch helix formation mechanism [18] starts with three extruded bases of the BSL initially base pairing with the intron. Thus, these BSL bases serve as a toehold that anchors the BSL to the intron. The subsequent growth of the branch helix is suggested to occur via strand displacement, in which the intron is expected to replace the 5′-end of the BSL (Fig. 1). This results in the complete opening of the BSL [18, 19]. Strand displacement typically involves good base pair complementarity to energetically drive strand migration [1]. However, since introns typically exhibit limited base pair complementarity with the U2 snRNA, it remains unclear how the U2 snRNA can recognize the BS via a strand invasion mechanism, especially since the BS possesses only a short conserved motif characterized by a significant degree of degeneracy [4, 27, 28] (Fig. 1).

Here, we combined all-atom and coarse-grained molecular dynamics (MD) simulations to elucidate how the U2 snRNA recognizes the branch sequence through strand invasion despite weak complementarity. We show that the release of the TAT-SF1 protein triggers the relaxation of a supercoiled, high-energy conformation of the U2 branch-stem loop, which drives strand displacement through a “loaded-spring” mechanism. This work reveals the physical basis of U2-mediated branch-site recognition and provides a dynamic framework for understanding early spliceosome assembly.

## Materials and methods

### Model building

**Figure 2.**
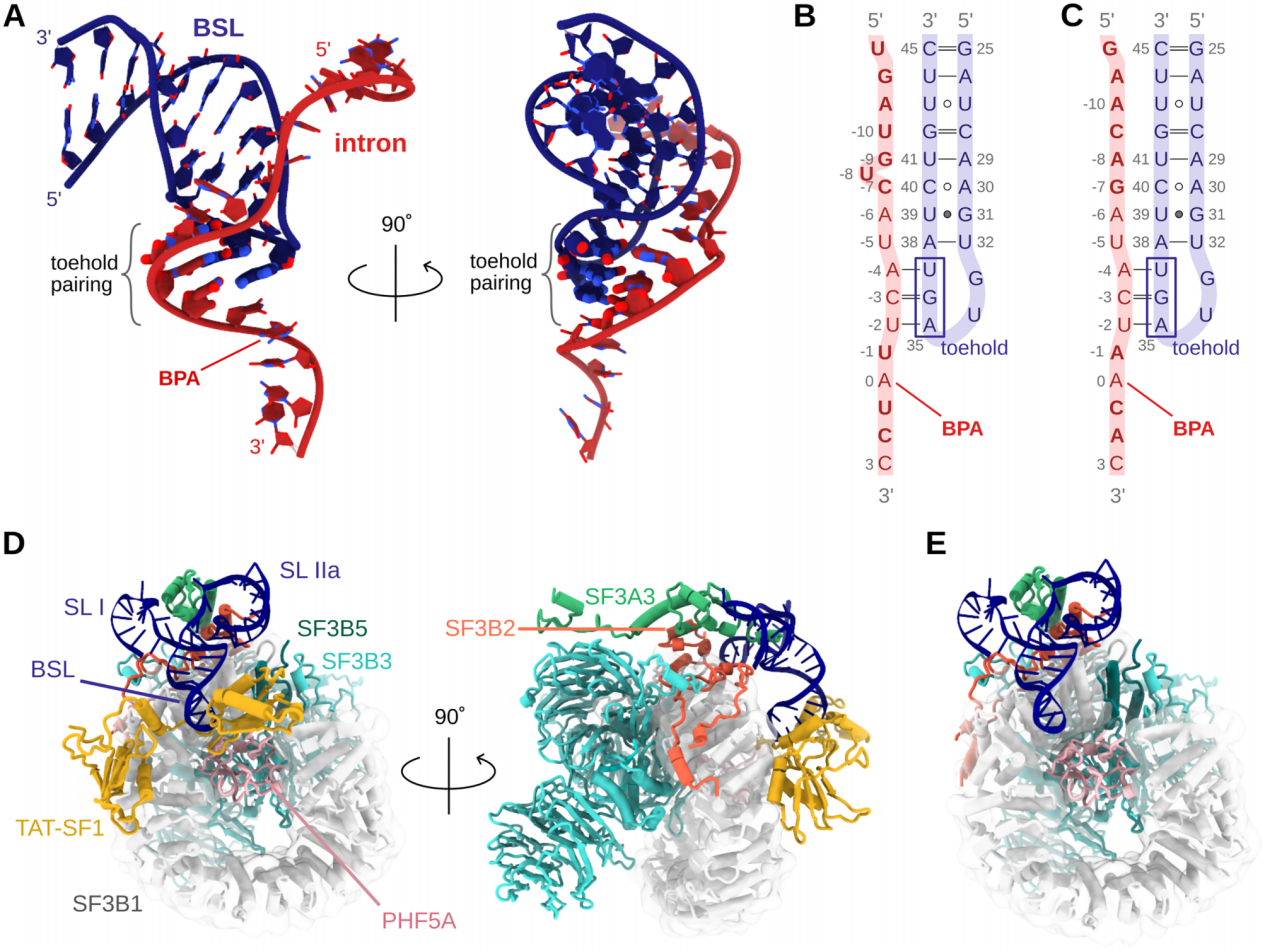
Models systems used in simulations. (**A**) Intron-Branch Stem Loop (BSL) model. Base pairs forming the toehold are shown with thicker sticks. Hydrogen atoms are omitted for clarity. Schematic representation of the intron-BSL model with an intron sequence having partial (**B**) and (**C**) full base pair complementarity to the BSL sequences. Residues differing in (B) and (C) are highlighted in bold. (**D**) 17S U2/SF3b model system. (**E**) 17S U2/SF3b model system without TAT-SF1.

The all-atom model of the intron-BSL system with the putative three base-pairs-long toehold was built based on the cryo-EM structure of the human 17S U2 snRNP/SF3b complex (PDB ID: 7EVO [17]), considering the BSL structure only. The intron was instead modelled as an A-RNA helix using the Nucleic Acid Builder of AmberTools [29]. The resulting helix was docked onto the BSL by aligning the three bases of the intron-complementary strand to the three bases of the toehold of the U2 BSL. The remaining strand of the A-RNA helix was then removed. In the resulting model (Fig. 2A), two base pairs of the toehold (U37 and G36) formed canonical base pairs with the intron (A−4 and C−3), i.e. A−4:U37 and C−3:G36. Conversely, one base pair, U−2:A35, was slightly distorted and formed only one hydrogen bond. Steric clashes between the 5′-end of the intron and the U2-BSL were manually resolved by remodelling the intron structure.

We built two intron-BSL RNA models considering intron sequences with partial and full base-pair complementarity to the U2 snRNA (Fig. 2B and C). In the first model, the intron had the so-called MINX sequence, commonly used in experimental studies and for which structural information is available. In the second model, the intron sequence was fully complementary to the U2 snRNA. We also prepared four variations of the first model, which featured (i) an A−6U mutation, resulting in an even lower degree of complementarity to the U2 snRNA, (ii) an idealized, canonically paired BSL stem, prepared by substituting nucleotides of the 5′-strand of the stem, (iii) a variation combining both the A−6U mutation and an idealized (canonical) BSL stem, and, lastly, (iv) a variant featuring pseudouridines (Ψs) at positions 34 and 37 in the BSL loop. Overall, we considered six possible variants of the intron/BSL sequences.

Finally, we built a model of the human 17S U2/SF3b system containing U2 snRNA residues 12 − 65 (SL I, BSL, and SL II stem-loops), the SF3B1, SF3B3, SF3B5, PHF5A, and TAT-SF1 proteins, and portions of SF3A3 and SF3B2 adjacent to the RNA (Fig. 2D and E). This model was based on the structure reported in PDB ID 7EVO. Missing protein loops were modelled using RoseTTA [30] and the missing U2 snRNA residues of the SL I loop were modelled with the SimRNA web server [31]. The N- and C-termini of PHF5A were capped with acetyl and methylamine groups (ACE and NME residues in Amber), respectively. One set of the 17S U2/SF3b simulations was run with the TAT-SF1 protein, and another set with TAT-SF1 removed.

### Classical MD simulations

Topologies of the different models were built using the t-leap module of the AmberTools suite. AMBER force fields (FFs) OL3 [32–35] and ff14SB [32, 33, 36, 37] were used for the RNA and protein, respectively. The molecules were solvated in octahedral boxes of TIP3P water [38] with a minimal distance between the solute and box border of 12 Å for the RNA-only systems, and 20 Å for the 17S U2/SF3b complex. Excess KCl salt at a 0.15 M concentration was added to all systems; the monovalent ions were described using Li&Merz 12-6 parameters [39]. Zinc ions, coordinated by the PHF5A and SF3A3 proteins in the 17S U2/SF3b complex, were covalently bound to the protein. The ZAFF force field [40] was used for the zinc sites, and the parameters for the zinc site of SF3A3 were derived following the ZAFF protocol available at https://ambermd.org/tutorials/advanced/tutorial20/ZAFF.php. For the pseudouridines (Ψs), we used recent modXNA parameters [41], loading only the Ψ-specific parameters to t-leap. The parameter files are available in the (Supplementary Information SI).

Overall, the solvated systems contained approximately 36 000−37 000 atoms and 328 000−390 000 atoms for the RNA-only and the 17S U2/SF3b systems, respectively.

Mg²⁺ ions were not included in the present simulations. Their modelling would be challenging, as their positions, if present, are not resolved in available cryo-EM structures of A-like spliceosomes, and their coordination and exchange dynamics are difficult to capture reliably with currently available FFs [42]. A hydrated Mg²⁺ ion was resolved in the X-ray crystal structure of a branch helix with G at the –1 position at a relatively high (10 mM) concentration of MgCl_2_, but no Mg^2+^ ion is present at that site when A is present at position −1, like in the sequences used here [43]. Experimental evidence shows that spliceosome assembly stalls at the B complex at low Mg²⁺ (∼0.3 mM), indicating that earlier steps, including strand invasion, can occur with minimal Mg²⁺ [44]. This suggests Mg²⁺ is not a primary driver of these RNA rearrangements, although its potential modulatory role cannot be excluded. Nevertheless, to investigate if positively charged ions may localize in the vicinity of the RNA during MD simulations, we performed an analysis of the K^+^ ion distribution over the MD simulation trajectories. This analysis showed the expected transient K^+^ binding to phosphate oxygen atoms and acceptor groups of the bases, including the loop region (Supplementary Table S1) [45].

To accelerate sampling in the unbiased MD simulations, we also employed two modified variants of the AMBER FF: namely, hydrogen-bond fix (HB-fix) [46, 47] and stacking-fix (sta-fix) [48] modifications. In the first variant, the short-ranged HB-fix potential adds an energetic barrier (1 kcal/mol) to the breakage of the formed hydrogen bond. Notably, this barrier applies only when the hydrogen bond is spontaneously formed, thus contributing to stabilizing it without preventing its breakage. The HB-fix potential was applied to the base pairs of the branch helix that were expected to form, including the toehold base pairs. In this manner, the conformational equilibrium of the RNA was shifted towards the formation of the branch helix without applying any biasing force. Conversely, the sta-fix modification, developed to prevent excessive RNA self-interactions and to enhance sampling, scales down van der Waals interactions between specific RNA atoms. This allows for a more efficient exploration of the conformational space. Here, a scaling factor of 0.9 was applied to all RNA nucleotides.

Additionally, to prevent spurious intron circularization—specifically the sticking of the 5′ and 3′-ends of the intron together—MD simulations of the intron-BSL constructs were performed by applying a wall of 30 Å to the end-to-end distance of the phosphorus atoms at the 5′- and 3′-termini of the intron.

Each system was equilibrated following an established protocol [49], which consisted of 11 steps of minimizations and short 1 ns-long MD runs with positional restraints decreasing from 5 to 0.5 kcal/mol. This was followed by a 1 ns-long unbiased MD simulation. After equilibration, the production MD simulations were run using the GROMACS program [50] with a 2 fs integration step, keeping bonds involving hydrogen atoms restrained with the LINCS algorithm. Electrostatics in the periodic boundary field were treated with Particle Mesh Ewald method with a cutoff of 10 Å for short-range interactions. The temperature of 298.16 K was maintained with the v-rescale thermostat [51], while the Parrinello-Rahman barostat [52] was used to control the pressure. The velocities from the equilibrated states were randomized at the beginning of each production replica to heterogenize the sampling.

A summary of the MD simulations performed is reported in the Supplementary Table S2. Unlike classical statistical experiments, MD simulations of such large systems do not sample independent events from a well-defined distribution. Due to finite sampling, the simulation outcomes are subject to random fluctuations and are sensitive to initial conditions. Thus, to increase their statistical significance, MD simulations are typically performed using multiple independent replicas and the collective behaviour is analysed. In this context, the fraction of trajectories showing a given behaviour should not be interpreted as a direct probability but rather as qualitative trend. When an event is observed multiple times in multiple independent MD simulations, it proves that it is not rare or artefactual, but an accessible and recurring pathway. Moreover, we further validate the reliability of the simulation results by verifying that the observed event is consistent with structural and energetic constraints derived from experimental data, such as cryo-EM structures.

For each combination of intron-BSL construct and FF setup, we performed 10 replicas, resulting in a total of 160 MD simulation replicas. All these simulations were run for at least 500 ns and then, after a visual inspection, either prolonged up to 2 μs or terminated if the trajectories became trapped in states preventing the formation of the branch helix. Since most base pairing events leading to the formation of the branch helix occurred in the early simulation stages (Supplementary Table S3), we argue that running multiple short MD simulation replicas allows for better sampling of branch helix formation than a few longer MD simulations [53].

MD simulations of the 17S U2/SF3b model were run in two replicas for the complex with the TAT-SF1 protein, and in four replicas for the complex where the TAT-SF1 protein was removed. All replicas were simulated for 2 μs. Helical parameters were calculated using the x3-DNA program [54].

### Metadynamics simulations

To estimate the energetics associated with the formation of the branch helix at three base pairs flanking the toehold, we performed well-tempered metadynamics simulations [55]. Here, we considered two small RNA-only models, with the intron having either full or partial base pair complementarity to the BSL. These models contained the U2-BSL’s nucleotides from U28 to G42 and the intron’s nucleotides from C/G−7 to C−3. To increase their stability, the models were capped with one G:C pair at the BSL terminus, and by another G:C pair at the toehold, effectively replacing the native U27:U43 and U−2:A35 base pairs of the BSL and intron-BSL duplex, respectively (Supplementary Fig. S1).

The well-tempered metadynamics simulations were run using two collective variables (CVs). We selected as the CV the εRMSD, a geometrical descriptor capturing the relative positions and orientations of the bases with respect to a reference structure [56]. This CV distinguishes different base-pairing patterns better than the canonical RMSD metric. For this reason, εRMSD is routinely applied to study the conformational remodelling of nucleic acids [57]. Here, we considered the εRMSD to both the BSL and the branch helix. As a reference structure for the εRMSD to the BSL, we used the structure deposited in PBD ID: 7EVO [17]. Conversely, the εRMSD to the branch helix with a partially complementary intron was calculated using a reference structure deposited in PDB ID: 7QTT [58]. Finally, the εRMSD to the branch helix, formed with a fully complementary intron sequence, was calculated with respect to a reference canonical A-RNA helix structure built using the Nucleic Acid Builder of the AmberTools suite. Aiming to simulate the initial steps of strand invasion, we only biased the disruption/formation of the first three base pairs beyond the toehold. Namely, these are the intra-BSL base pairs U32:A28, G31:U39, and A30:C40, and the branch helix base pairs U−5:A38, A−6:U39, and C/G−7:C40. The εRMSD cutoff in the metadynamics simulations was set to 3.2 to cover larger areas of the conformational space [57]. To build the bias, we used Gaussian hills with a width of 0.1, deposited every 200 steps on a grid ranging from 0 to 5. The bias factor was set to 10, and the τ value, controlling the Gaussian hill height was set to 70. The simulations were run using PLUMED-patched GROMACS [59].

To prevent the system from becoming trapped in conformations far from the target state, we applied wall restraints to limit the conformational space explored during the simulations. Namely, we applied wall restraints on (i) the hydrogen bond distances of the base pairs that were not involved in the BSL-branch helix exchange; (ii) the distance of the two base pairs enclosing the biased bases (A−4:U37 and A29:U41); (iii) the backbone conformation of the intron strand using pseudo-dihedrals defined over C4′ and P atoms [60]; (iv) an upper wall on the cumulative εRMSD; and (v) a lower wall on the (εRMSD_BSL_ – εRMSD_BH_)^2^ – min(εRMSD_BSL_,εRMSD_BH_)^2^ value, where BH denotes the branch helix. We then ran four metadynamics simulations differing in the the extent of backbone walls applied. Namely, in the first replica of the partially complementary intron sequence, we applied a restraint wall on the backbone of the C−7 and A−6 bases, while in the second replica, the restraint wall was extended to the U−5 and A−4 bases. Conversely, in the first replica of the fully complementary intron, we applied analogous restraint walls to G−7, A−6, U−5, and A−4, while in the second replica, the same pseudo-diherals were targeted, but they were restrained to a narrower value range. Details are listed in Supplementary Methods section. Applying these restraints enhanced the sampling of BSL-branch helix transitions but did not fully eliminate the exploration of off-pathway states. Therefore, trajectory segments corresponding to off-pathway states were excluded from the analysis (see Supplementary Methods and Supplementart Figs S2 and S3 for details).

Free energies were calculated using reweighting. We considered all frames with an εRMSD to the reference state lower than 0.7 as belonging to a specific free energy minimum. An εRMSD cutoff of 2.4 was used for analysis. For the partially complementary intron, the free energy was evaluated using a time-averaged bias potential. The averaging was done over the full trajectory, except the first 1 µs. For the fully complementary intron, raw (non-averaged) bias files were used due to lower convergence. The errors were estimated using bootstrap analysis.

### Coarse-grained simulations

For coarse-grained simulations, we used the oxRNA model [61], which approximates nucleotides as rigid bodies with two beads—one for the base, and one for the backbone. This model has been successfully used for strand displacement simulations [62, 63]. oxRNA defines interaction potentials for A:U, G:C, and G:U base pairs only. Therefore, we performed these simulations considering only the fully complementary intron sequence. The starting coordinates and topology were generated from an all-atom structure, as shown in Fig. 2A, using the oxView server [64]. We then ran CG simulations in 100 replicas in which the structures were first relaxed via Monte Carlo and molecular dynamics, followed by a production run for 25 × 10^6^ steps using a time step of 0.001 in the oxRNA internal time units. This formally corresponds to 750 ns. However, the CG nature of the model makes a direct comparison to real timescales non-trivial, and the actual corresponding physical timescales are typically significantly longer [61, 65]. Therefore, we focused only on the relative timing of individual trajectories in branch helix formation, without attempting to assign physical timescales to the CG trajectories.

The resulting trajectories were analysed using the OAT package [66]. A base pair was considered formed if its hydrogen-bond energy was below −0.2 oxRNA internal units. The BPA was considered to be bulged out if its neighbouring base pairs A−1:U34 and C1:G33 were formed, the stacking energy of the A−1/C1 pair and of the U34/G33 pair was below zero, and the stacking energy of the BPA (A0) and both its neighbouring residues (A−1 and C1) was zero.

## Results

### A slip-stranded conformation of BSL triggers branch helix formation

To monitor the initial steps of the intron strand invasion into the U2-BSL, we considered two models, differing in the extent (partial or full) of the base pair complementarity of the intron to the BSL (Fig. 2B and C). The starting structures accounted only for the initial binding to the toehold. Namely, the U−2, C−3, and U−4 bases of the intron were paired to the A35, G36, and U37 toehold nucleotides of the BSL, respectively. For each model, 30 independent MD simulations of 0.5–2.0 μs were carried out. In detail, three FF variations were considered per model, with 10 replicas executed for each FF setting. Namely, we used the standard AMBER OL3 FF [32–35]; a variant of this FF, which stabilized all hydrogen-bonds of the branch-helix base pairs (HB-fix FF variant) [46, 47]; and a FF variant in which RNA self-interactions were also weakened (combining HB-fix and sta-fix corrections) [48].

MD simulations, performed with the standard AMBER OL3 FF, revealed that the U−2:A35 base pair of the toehold was the least stable among the three toehold base pairs. Indeed, U−2:A35 remained fully stable only in ∼40% of the MD simulation trajectories with the standard FF, likely due to a geometric constraint of the BSL loop on the A35 backbone conformation. Instead, the two remaining bases, G36 and U37 of the toehold, formed stable interactions with C−3 and A−4 of the intron, respectively. As expected, greater stability of all toehold base pairs was instead observed when introducing the HB-fix variant of the FF.

**Figure 3.**
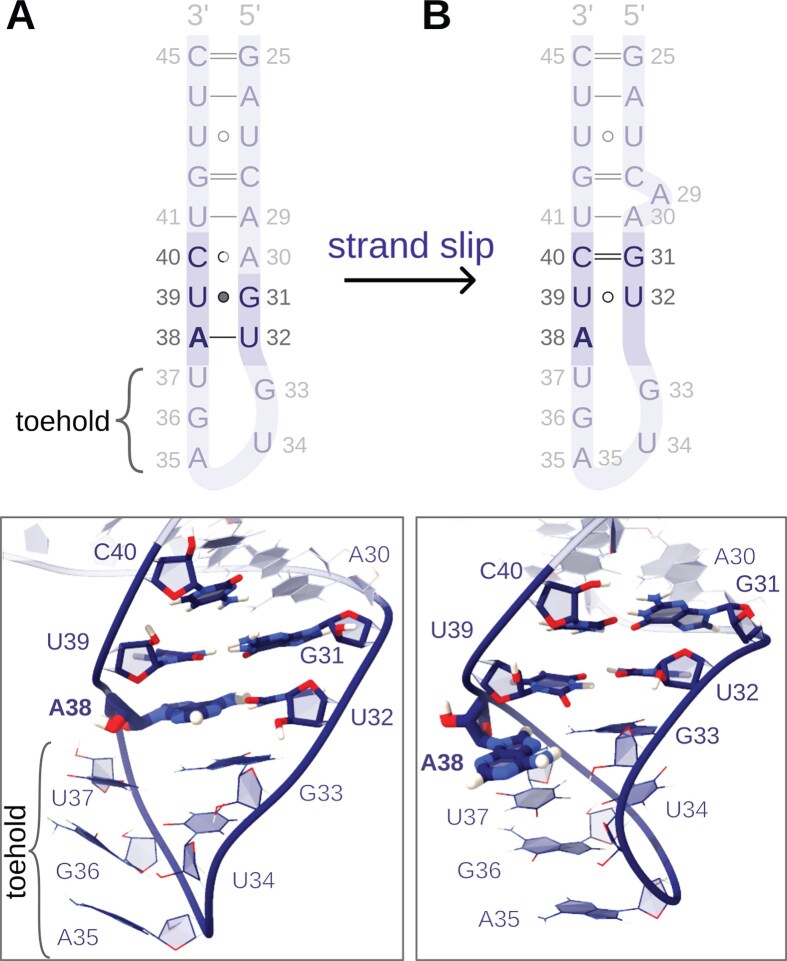
Conformational rearrangement of the BSL. (**A**) The initial conformation of the BSL and (**B**) BSL slip-stranded state. In the sketch above, residues involved in the slip-stranded state are highlighted. In the structural models below, the residues involved in the slip-stranded state are shown in sticks, while the remaining residues are shown in lines. The intron, bound to the toehold residues, is omitted for clarity.

**Figure 4.**
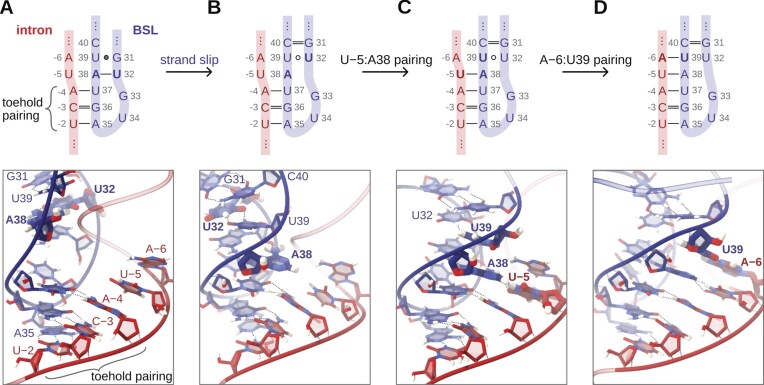
Early steps of branch helix formation via the slip-stranded path. Schematic representations (top panel), and snapshots (bottom panels) of the intermediates observed in all-atom simulations. Residues involved in the formation of the toehold pairing and BSL:intron remodelling steps are shown in sticks; the remaining residues are not shown. (**A**) A three-base-pair long toehold is initially formed, based on the base pairing interactions between A−4:U37, C−3:G36, and U−2:A35 of the intron and BSL, respectively. The BSL contains the U32:A38 and G31:U39 base pairs. (**B**) The BSL transits to the slip-stranded conformation with the formation of U32:U39 and G31:C40 base pairs, while A38 remains unpaired and protrudes towards the intron. (**C**) A38 pairs with U−5 of the intron. (**D**) U39 pairs with A−6 of the intron, further extending the growing branch helix.

Importantly, almost half of all simulations (27/60) sampled spontaneous toehold extension. Moreover, while the standard AMBER FF formed up to two new base pairs in the intron’s 5′-end direction, the modified FFs aided the formation of three (HB-fix) and four (HB-fix and sta-fix) new base pairs (Supplementary Table S2 and Supplementary Figs S4−S6). Notably, in most of the trajectories leading to the branch helix extension (18/27), we observed the formation of a slip-stranded BSL intermediate preceding the extension (Figs 3 and 4). The same BSL slip-stranded state was also observed in eight additional simulations, although these simulations did not result in a productive toehold extension. The slip-stranded state featured an intramolecular register shift in the BSL base pairing, which resulted in a shift of the BSL 3′-strand by one base towards the 5′-end. As a result, the A38:U32 base pair of the BSL broke and a U32:U39 pair newly formed, thus liberating A38 for the first step of branch helix extension (Fig. 3). This slip-stranded conformation also aided the subsequent BSL displacement step. Here, U39 of the BSL formed a weak intra-BSL base pair with U32, facilitating the binding of U39 to the intron’s A−6. After the formation of the slip-stranded state, a propagation of the register shift to the flanking bases was commonly observed. This induced the formation of a second slip-stranded pair, G31:C40, and, occasionally, a third, A30:U41 (Fig. 3). Upon formation of the A30:U41 base pair, A29 remained unbound, inducing a destabilization of the C28:G42 base pair, which resulted in C28 bulging. Sometimes, the formation of the slip-stranded states was only short-lived (i.e. ns-long for the U32:U39 pair). Yet, this was sufficient to trigger the formation of the new branch helix base pair.

Conversely, in the few MD simulation replicas, where the initial slip-stranded event was not sampled, the formation of the first two base pairs (U−5:A38 and A−6:U39) of the branch helix occurred immediately after the opening of the A38:U32 and U39:G31 intra-BSL base pairs. Occasionally, the base-pair exchange proceeded via transient intermediate states in which a nucleotide engaged simultaneously with both its original and incoming pairing partners, reminiscent of triple-like interactions (Supplementary Fig. S7). This mechanism also applied to the formation of the third (C/G−7:C40) and fourth (A−8:U41) base pairs of the branch helix.

Extension of the branch helix at the other extremity of the toehold (intron 3′-direction) was observed only in four replicas out of 60. In all of them, the A/U−1:U34 base pair formed after a portion of the BSL was already unwound (Supplementary Fig. S8). We thus suggest that U34 cannot bind the intron beforehand (i.e. by forming an additional toehold base pair) due to steric constraints imposed by the BSL loop (Supplementary Fig. S8).

We complemented these results by running two additional sets of simulations. The first set was performed with an intron featuring further reduced base pair complementarity to the U2 snRNA. This was achieved by introducing the A−6U mutation in the partially complementary (MINX) intron sequence. As a result, when using the standard FF, the branch helix grew by two base pairs in only one MD simulation replica (out of ten). Instead, when considering the HB-fix or sta-fix FF variants, we observed growth by three base pairs, with a frequency comparable to that observed for the original partially complementary sequence (Supplementary Figs S4 and S9).

In the second simulation set, we used an idealized BSL model, formed only by canonical base pairs rather than non-canonical ones. This construct can still form the slip-stranded U32:U39 pair. With this sequence, we observed branch helix extension to a maximum of two base pairs. Nevertheless, none of the 60 replicas sampled a branch helix extension beyond the second base pair—an outcome that contrasts with all simulations of the native BSL, including those carrying the A6–U mutation (Supplementary Figs S4, S10, and S11). As expected, the idealized BSL exhibited lower stem flexibility compared with the native BSL (Supplementary Fig. S12). These simulations indicate that enforcing canonical base pairing reduces the intrinsic flexibility of the BSL and limits branch helix propagation.

### Early steps of branch helix formation are energetically favourable

**Figure 5.**
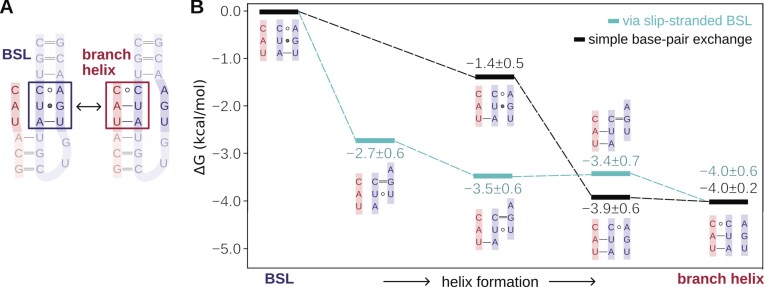
Energetics of the first three steps of toehold extension. (**A**) Model system used in metadynamics simulations featuring an intron with a partially complementary sequence to the BSL. The boxes mark the bases that were biased in the metadynamics simulations. (**B**) Scheme of the relative free energy (kcal/mol) of the two competitive pathways (slip-stranded, cyan lines; and base-pair exchange, black lines) leading to the formation of the branch helix. The scheme shows only the residues involved in interaction remodelling. Free energies are averaged over two metadynamics replicas (see Supplementary Fig. S13 for individual runs).

To characterize the energetics of branch helix formation, we performed sets of two metadynamics simulations on the reduced BSL-intron model, considering intron sequences partially and fully complementary to the BSL (Fig. 5A). Each metadynamics simulation of the partially complementary intron sequence sampled 13 transitions from BSL to the branch helix, which were found to be energetically favourable; the calculated free energy differences between the starting BSL and final branch helix states (Δ*G*_BH-BSL_) were −4.6 ± 1.3 and −3.4 ± 1.9 kcal/mol in the two replicas. This process became even more energetically favourable when considering the fully complementary sequence (Δ*G*_BH-BSL_= −9.3 ± 4.8 and −5.0 ± 3.1 in the two replicas). However, with the fully complementary sequence, the BSL to branch helix transition was sampled only 4–5 times per simulation. Hence, due to the limited sampling of the transitions, these metadynamics simulations are affected by larger errors. As such, although these simulations can provide only qualitative information, they still suggest that, irrespective of the intron sequence, the formation of the first branch helix base pairs is thermodynamically more favourable than the intramolecular BSL base pairing.

Spontaneous formation of the slip-stranded state was also sampled in the metadynamics simulations, despite not being explicitly enforced by the CVs used. Notably, the first and the second steps of the slip-stranded pathway (register shift of the U−5:A38 pairing) are energetically more favourable than the starting BSL conformation (Fig. 5, Δ*G*_BH-BSL_= −2.7 ± 0.6 and −3.5 ± 0.6, respectively), with the first step being the most energetically favourable. Conversely, the following steps of branch helix extension are, within the error of our calculations, isoenergetic. An alternative path, which avoided the formation of slip-stranded intermediates, was also sampled. As discussed above, this path consisted of an exchange of intra-BSL base pairs in favour of intron-BSL base pairs. In this base pair exchange path, the formation of the first (U37:A−4) and the second (A38:U−5) base pairs was thermodynamically favourable (−1.4 ± 0.5 and −3.9 ± 0.6, respectively). Importantly, a qualitative estimate of the free energy barriers for the conversion between states indicates that they were small (~2 kcal/mol, Supplementary Fig. S14) in both the slip stranded and base pair exchange paths, suggesting that all states were thermally accessible. Thus, although the slip-stranded pathway appears to be slightly more favourable, both pathways seem kinetically and thermodynamically viable at room temperature.

### TAT-SF1 prevents formation of the slip-stranded state of BSL in the 17S U2/SF3b complex

**Figure 6.**
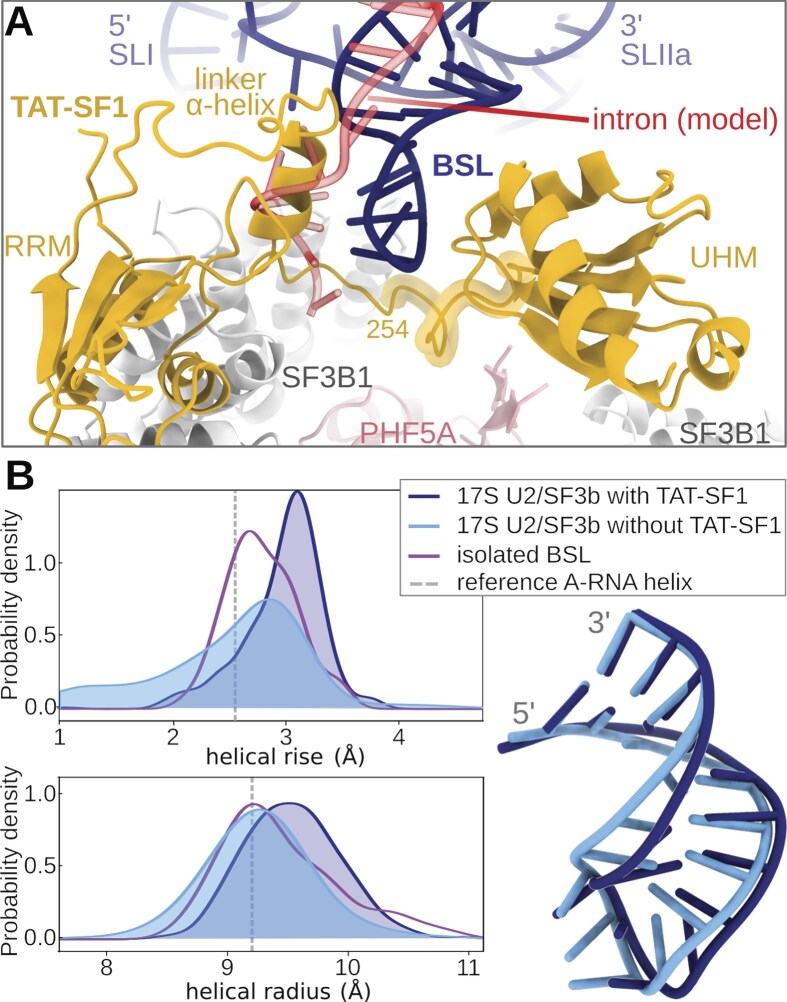
BSL structure. (**A**) Magnified view of the 17S U2/SF3b with TAT-SF1 masking the U2 snRNA BSL. The red transparent cartoon marks the hypothetical position of the intron when bound to the BSL toehold. The segment on the TAT-SF1 linker, highlighted with a yellow shadow, corresponds to residues 254–264. (**B**) Helical parameters of the BSL stem. The left panel shows the distribution of helical rise values, defined as the distance between the planes of the consecutive base pairs and of the helical radius values, defined as the distance between the helical axis and phosphorus atoms. The plots contain data from two merged trajectories of the 17S U2/SF3b with TAT-SF1, and four merged trajectories of the 17S U2/SF3b without TAT-SF1. For a reference, data from a 250 ns-long trajectory of isolated BSL are also shown. Reference values for an A-RNA helix were taken from [54]. The right panel shows the corresponding average BSL structures with (dark blue) and without (light blue) the TAT-SF1 protein. Structures are aligned with respect to the two terminal base pairs.

Comparison of our toehold-bound model with the 17S U2 snRNP cryo-EM structure shows that the TAT-SF1 linker sterically precludes premature intron binding to the U2 snRNA toehold (Fig. 6A). However, it is unclear whether TAT-SF1 acts only as a steric block or also impacts BSL stability. We therefore assessed the BSL dynamics within the U2/SF3b protein environment and the impact of TAT-SF1 by simulating the 17S U2/SF3b particle either in the presence or absence of TAT-SF1 (Fig. 2D and E). The model without TAT-SF1 represents a hypothetical state formed after TAT-SF1 dissociation aided by PRP5, but in which the intron is not yet bound.

**Figure 7.**
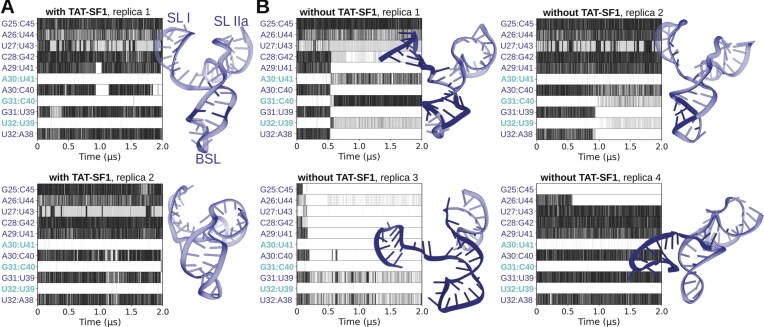
BSL conformation in the presence and absence of TAT-SF1 protein. MD simulations of the 17S U2/SF3b complex were performed with (**A**), and without the TAT-SF1 protein (**B**). Graphs show the evolution of base pairing formation versus simulation time (μs) in different models and replicas. The base pairs labelled in cyan participate in the strand-slipped conformation. Black, grey, and white colours indicate that the base pair is fully formed, weakly bound (suboptimal pairing geometry, lower number of hydrogen bonds), or absent, respectively, during the MD simulation trajectory. The U2 snRNA structures sampled in the last snapshot of each trajectory are shown on the sides of the trajectory with restructured bases highlighted in a darker tone.

During multi-replica, 2 µs-long MD simulations, the U2/SF3b core was structurally stable, while the TAT-SF1 protein, BSL, and SL I stem-loop of U2 snRNA experienced large fluctuations, consistent with the weak electron densities observed in cryo-EM experiments [17, 67] (Supplementary Fig. S15). Nevertheless, the BSL pairing remained stable (Fig. 7A). In contrast, TAT-SF1 removal resulted in increased fluctuations of the BSL and triggered its restructuring (Fig. 7B). Namely, two MD simulation replicas, where TAT-SF1 was absent, sampled the formation of the slip-stranded BSL. Conversely, in the other two replicas, we observed (i) a marked restructuring of the BSL via a complete change of the base pairing pattern and (ii) SL I relocation towards the BSL, causing a rupture of the G25:C45 and A26:U44 base pairs while the region adjacent to the BSL loop remained stable. As such, MD simulations reveal that the BSL slip-stranded state can also form within the 17S U2/SF3b complex.

The spontaneous and readily accessible BSL rearrangements observed in the absence of TAT-SF1 suggest that this protein might keep the BSL in a structurally tense state. Indeed, an analysis of helical parameters revealed that TAT-SF1 stabilized an overtwisted BSL conformation characterized by a larger helical rise and radius. This contrasts notably with the BSL in isolation or within the 17S U2 snRNP that lacks the TAT-SF1 protein (Fig. 6B).

**Figure 8.**
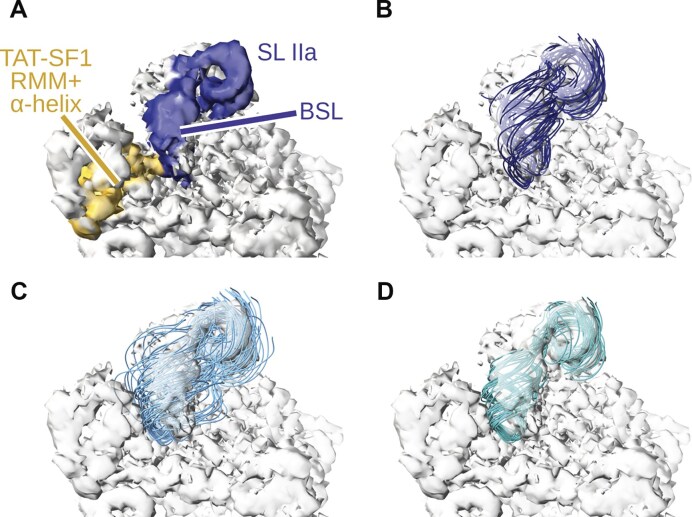
Fitting of MD simulation conformational ensembles to cryo-EM-derived electron density. (**A**) Reference density of the 17S U2 snRNP (EMDB 13793, [67]). Regions corresponding to U2 snRNA and TAT-SF1 are coloured in blue and yellow, respectively. (**B**) Conformational ensemble of BSL + SLIIa from MD simulations of the U2/SF3b complex with TAT-SF1. SF3B1 was used as a reference for fitting. (**C**) Conformational ensemble of BSL + SLIIa from MD simulations of the U2/SF3b complex without TAT-SF1. (**D**) Selected frames of the conformational ensemble corresponding to the slip-stranded state.

Consistent with this, comparison of MD simulation ensembles of the BSL in the presence and absence of TAT-SF1 shows differences in the position of the BSL loop tip and the adjacent stem region (Fig. 8 and Supplementary Fig. S16). Specifically, aligning the MD-generated BSL ensemble lacking TAT-SF1 with cryo-EM densities of the 17S U2/SF3b complex reveals noticeable deviations, including steric clashes with density corresponding to the TAT-SF1 linker α-helix. Notably, previous 3D classification of cryo-EM data indicated additional BSL conformations beyond the predominant “ground-state” model [67]. The conformational ensembles sampled in our MD simulations in the absence of TAT-SF1, including the slip-stranded state, experience a shift of the BSL stem in a similar direction to the observed density variations (Supplementary Fig. S16C). While the presence of such BSL states within the full 17S U2/SF3b assembly cannot be excluded, our results indicate that TAT-SF1 constrains the BSL into the ground-state conformation. Accordingly, dissociation of TAT-SF1 is likely the key event that shifts the equilibrium towards alternative, energetically favourable BSL conformations.

To understand the mechanism by which TAT-SF1 stabilizes the overtwisted BSL conformation, we analysed the RNA−protein interactions in the MD trajectories. Although a quantitative analysis is hindered by the limited sampling of the simulations, the flexible nature of the BSL/TAT-SF1 adduct, and the fact that a small portion of the linker (residues 257−263) was modelled, we could identify some general trends shared across MD simulation replicas. Indeed, MD simulations pinpointed that the strongest interactions between the BSL and TAT-SF1 are established between the tip of the BSL loop (A35 and G36) and the TAT-SF1 linker region (Arg254 to Arg264, Fig. 6A). Dynamic interactions are also formed between arginines of the UHM domain of TAT-SF1 and the RNA, while the α-helix of the linker (residues 236−246) establishes only transient interactions with the RNA (Supplementary Table S4). These results suggest that TAT-SF1 may restrain the BSL ensemble by anchoring the BSL loop with its linker and by providing a steric constraint via the linker α-helix.

### BSL nucleotide modifications stabilize the BSL loop

The native U2 snRNA is decorated with nucleotide modifications that may affect its dynamics (Fig. 1) [68]. Due to the current limited validation of the FF parameters for modified nucleobases [69, 70], we refrained from including all the modifications in our simulations. However, we searched for a minimal relevant model to assess their impact. To this end, we selected the pseudouridine modifications Ψ34 and Ψ37 of the BSL, which potentially appear to interact with other parts of the U2 snRNA or proteins (Supplementary Fig. S17). Considering only Ψ34 and Ψ37 in the BSL, we performed 10 MD simulations of the RNA-only construct (Fig. 2A) containing the partially complementary intron sequence.

**Figure 9.**
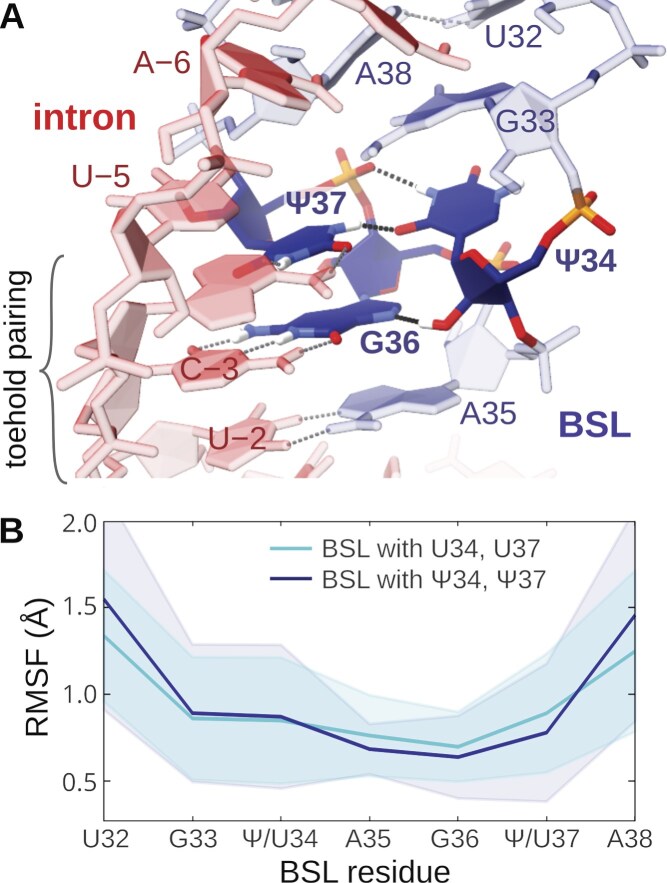
Molecular dynamics simulations of the intron-BSL construct with pseudouridines. (**A**) Hydrogen-bond network established by Ψ34 and Ψ37. (**B**) Root-mean-square fluctuations (RMSF, Å) of the BSL in ten simulation replicas without (cyan) and with (dark blue) Ψs in the BSL loop. The structures were aligned to the loop residues (G33-Ψ/U37); the RMSF of the stem is already affected by strand invasion. Data refer to MD simulations performed with the standard FF.

We observed that pseudouridines enable the formation of an additional H-bond in the BSL region, Ψ37(H1)-Ψ34(O4) (Fig. 9A) in all MD simulation replicas. This interaction also helps stabilize additional nearby H-bonds, which are also present, yet less persistent, without pseudouridines. Notably, the stability of these H-bonds appears correlated to that of the toehold base pairs (Supplementary Fig. S18), suggesting that the pseudouridine may contribute to the overall stabilization of the toehold-intron binding. The fluctuations of loop residues are also slightly reduced when pseudouridines are present (Fig. 9B). Nevertheless, the presence of pseudouridines did not alter the relative occurrence of the slip-stranded pathway versus the branch helix extension pathway (Supplementary Fig. S19).

Overall, the pseudouridine modifications seem to help stabilize the BSL conformation, without changing the mechanism of branch helix growth, indicating a role in fine-tuning the occurrence of strand invasion. Given that pseudouridines are known to stabilize RNA helices [43, 71–73], these modified bases, especially Ψ34, may also contribute to the stabilization of the fully formed branch helix.

### Coarse-grained simulations support the bidirectional model of the branch helix growth

**Figure 10.**
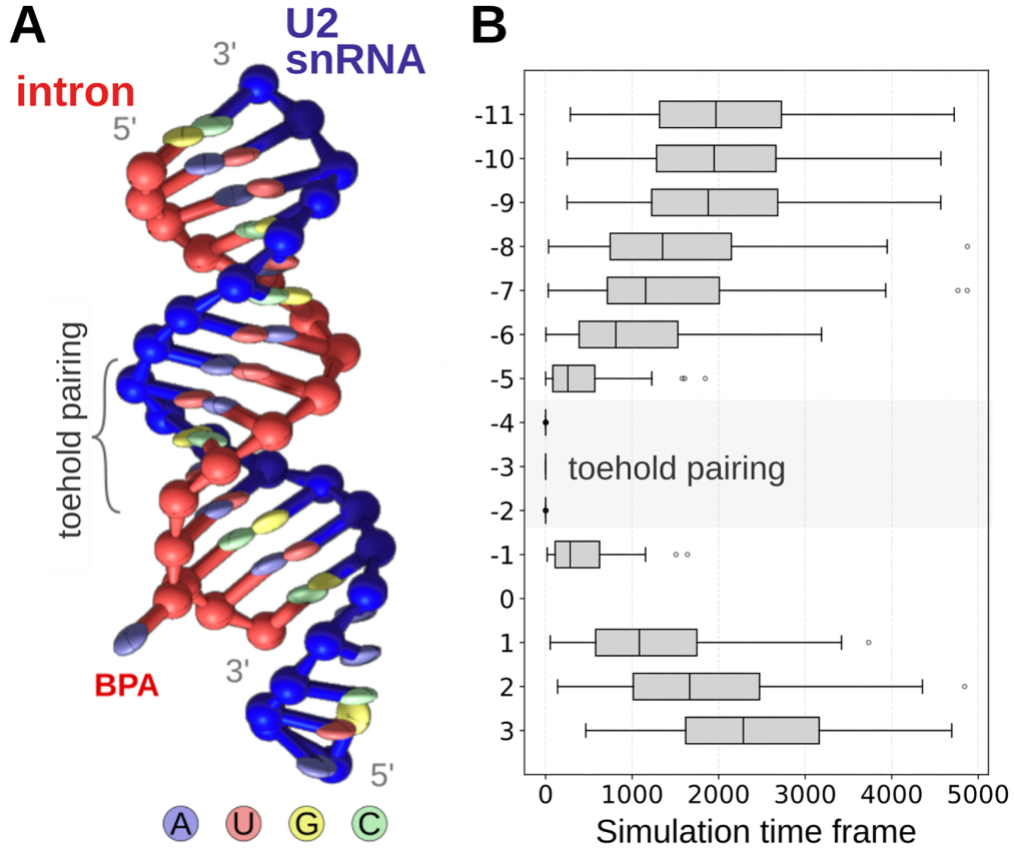
Branch helix formation in coarse-grained (CG) simulations. (**A**) Final structure with the full branch helix in the CG representation. (**B**) Statistics on branch helix base-pair formation versus simulation timeframe. The average is collected over 100 CG trajectories. The toehold-pairing area is marked by a grey box.

The only cryo-EM structure capturing strand invasion at an intermediate stage of branch duplex formation (Fig. 1D, [18]) was obtained in the presence of the splicing inhibitor spliceostatin A. Although this inhibitor was critical to stall the prespliceosome, it may also constrain helix propagation in the intron 3′ direction. In fact, our all-atom simulations of the intron-BSL construct suggested that the branch helix can grow bidirectionally once the BSL stem is loose enough (Supplementary Fig. S8). Thus, to further investigate strand invasion in the absence of the inhibitor, we complemented the cryo-EM model with coarse-grained simulations of the intron–BSL system. The CG model comes at the cost of losing atomic-level detail but allowed us to accelerate the sampling. To this end, we employed the oxRNA CG model, as it has already been successfully applied to study strand invasion [62, 63] (Fig. 10A and Supplementary Fig. S20). We simulated only the intron sequence fully complementary to the BSL.

By performing 100 CG simulation replicas starting from the toehold-bound state, we observed the complete formation of the branch helix in the vast majority of trajectories (83 runs). Consistent with the all-atom simulations, the formation of the branch helix proceeded in a stepwise manner. Only the terminal portion of the BSL (i.e. involving intron nucleotides from position −9 to −11) formed concertedly with complete BSL stem unwinding (Fig. 10B). Importantly, the growth of the branch helix started from the bases flanking the toehold and proceeded linearly in both directions at a similar speed (Fig. 10B). During the growth of the branch helix, the BPA remained intrahelical, being bulged out only after complete formation of the branch helix (Supplementary Fig. S21). In the intrahelical state, the BPA was mostly unpaired, pairing transiently with U34 or G33 (Supplementary Fig. S22). A more detailed analysis is provided in the Supplementary  Fig. S20. Given the limited accuracy of CG models and their inability to capture non-canonical base-pairs, we did not observe the slip-stranded state. Nevertheless, these simulations indicate that branch helix growth can proceed bidirectionally during branch-site recognition.

## Discussion

RNA conformational remodelling plays a crucial role in many pivotal biological processes, including translation regulation, protein synthesis, and pre-mRNA splicing [74–76]. During remodelling, RNA conformations typically adopt two mutually exclusive sets of base pairing interactions. Transitioning from one conformation to another requires breaking one set of base pairing interactions in favour of a new set. While RNA helices can be unwound by helicases in an ATP-dependent manner [77–80], RNA restructuring can also occur through a non-ATP-driven strand displacement mechanism [2]. In this process, the spontaneous dissociation of an RNA helix, which requires overcoming a high free energy barrier, is unlikely to serve as the primary mechanism for RNA remodelling. An alternative stepwise process, based on a progressive exchange of interactions, is more energetically viable and thus most likely to take place. The strand displacement mechanism, in which a single RNA strand invades a duplex and displaces one of the strands to hybridize with the other, falls into this category.

While RNA strand displacement is well characterized *in vitro* and used for nanotechnological applications [3, 81], its characterization in a cellular context lags. Among biological systems suggested to exert their biological function via strand displacement is the CRISPR–Cas system, where the spacer region of the guide RNA leads the complex to the target genome region and disrupts the DNA:DNA duplex to form an RNA:DNA duplex, a process known as R-loop propagation [82–84]. RNA rearrangement also occurs during co-transcriptional folding, as the nascent RNA begins to fold while being transcribed. Since the initial folding pattern of the RNA transcript does not necessarily represent the lowest-free-energy conformation of the entirely synthesized sequence, the RNA can rearrange its secondary structure through strand displacement as the transcription proceeds [85]. Some of the dramatic changes in riboswitch conformation in response to ligand binding have also been suggested to occur via a strand displacement process [86]. Finally, non-coding RNAs are also hypothesized to engage with their targets via strand displacement [87].

Among biological systems proposed to exploit strand displacement, spliceosome assembly represents a particularly intriguing case. Cryo-EM studies suggest that U2 snRNA recognizes the intronic branch sequence (BS) through a strand invasion process [18]. This mechanism, however, poses a puzzle: how can the intron spontaneously invade and replace the U2 snRNA branch-stem loop (BSL) pairing despite their typically limited base pair complementarity?

**Figure 11.**
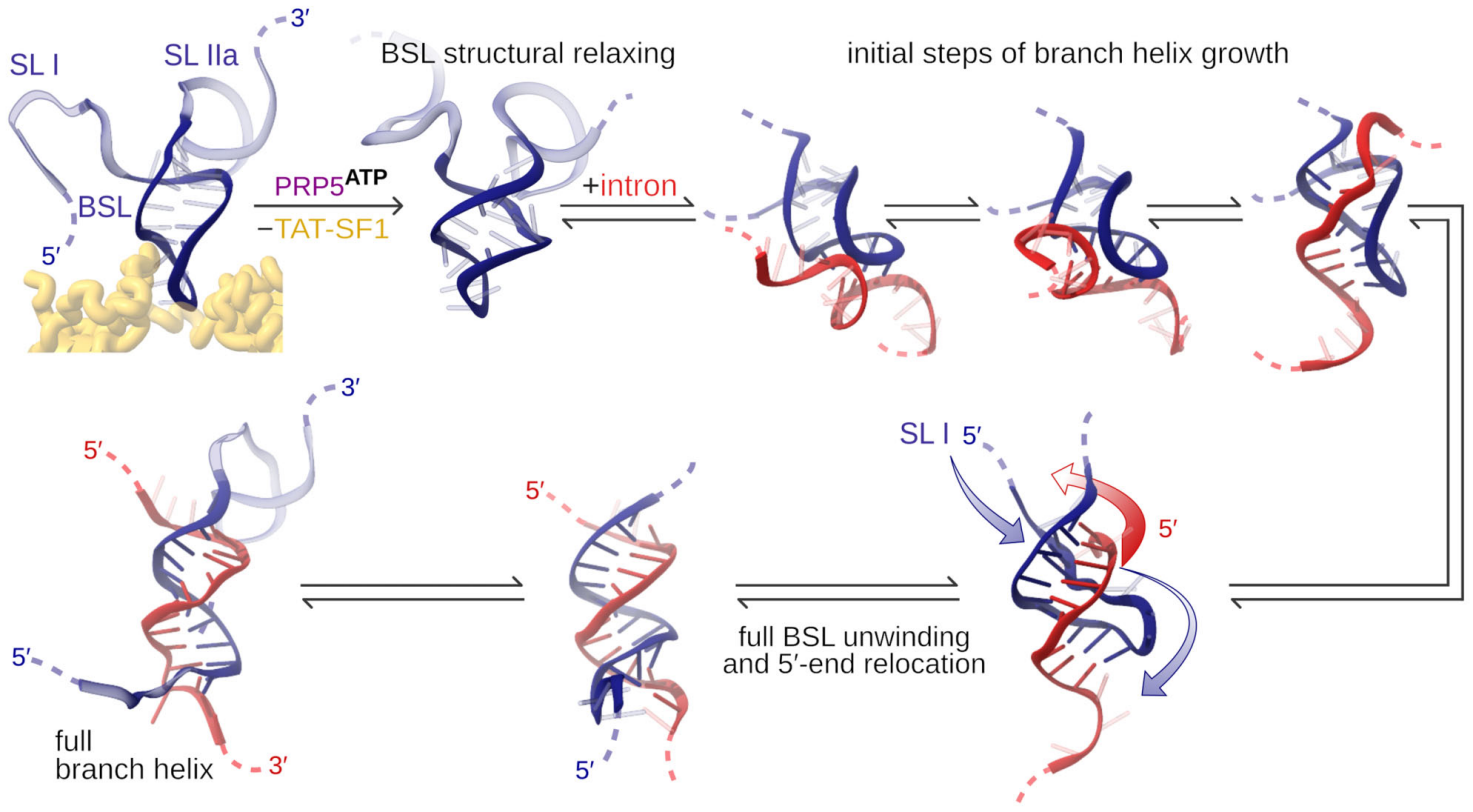
Proposed mechanism of intron-U2 hybridization. In the first stage, after TAT-SF1 is released, the BSL relaxes, the BS binds and quickly forms a few new base pairs with the aid of the slip-stranded BSL state (top row). The rest of the branch helix extension occurs bidirectionally and more slowly, requiring also a swap of the intron and U2 snRNA 5′-ends (bottom row, indicated by arrows). Namely, the SL I has to unwind and pass below the intron, while the intron has to pass in the opposite direction. This exchange is restricted by the presence of SL II and SF3B1 in the full U2/SF3b system (Supplementary Fig. S23).

Here, we provide a mechanistic answer to this question by revealing the physical basis of strand displacement during U2-mediated branch-site recognition. Using all-atom and coarse-grained MD simulations, we show that strand invasion can proceed spontaneously once the TAT-SF1 protein dissociates and the toehold region of the BSL engages in base pairing interactions with the intron. The key discovery is that TAT-SF1 maintains the BSL in a supercoiled, high-energy conformation, acting as a molecular latch that holds the U2 snRNA in a “loaded-spring” state (Fig. 11). Our MD simulations, showing that the BSL readily adopts multiple conformations—including a slip-stranded state—in the absence of TAT-SF1, support a model in which TAT-SF1 dissociation relaxes the strained configuration of the BSL, allowing it to unleash the stored twisting energy that drives the strand invasion.

The slip-stranded state observed in MD simulations features a register-shifted base-pairing pattern (Figs 3 and 4), which liberates A38 of the BSL for intron selection. This facilitates the readout of the intron at position −5, which pairs with A38. In principle, the BSL might adopt the slip-stranded state after the intron binds to the three-base toehold of the BSL, or before intron binding, thus creating a four-base-pair-long toehold. Such small RNA structural switching, involving non-canonical base pairs and/or loop-adjacent areas, occurs spontaneously on the μs timescale [88–90]. The relevance of the strand slippage mechanism observed here is supported by its recurrence in other biological processes, such as gene expression control [91] or guanine quadruplex folding [92–94]. As an alternative, a more conventional base-exchange pathway, bypassing the slip-stranded state, can also take place. In this path, which nevertheless appears to be slightly less energetically favourable, intra-BSL base pairs interactions are progressively lost in favour of the formation of inter-BSL-intron base pairs.

Branch-helix formation is thermodynamically favourable in both slip-strand and base-exchange pathways (Fig. 5). Both pathways are viable even when the intron has limited base pair complementarity to the BSL, although branch helix extension becomes less effective when the complementarity decreases (Supplementary Fig. S4). Importantly, in the presence of intron sequences with low complementarity to U2 snRNA, the branch helix growth may be additionally aided by the nucleotide modifications of the BSL, such as pseudouridylations and methylations, which have been shown to favour helical base paring [71–73, 95, 96]. Furthermore, the SF3B1 and SF3A2 form a channel that accommodates the branch helix by surface complementarity. It is likely that this composite groove facilitates and stabilizes the growth of branch helices of introns even when the complementarity is low. Finally, since the BSL stem distant from the toehold-forming region is characterized by a high base pairing complementarity—and it is therefore thermodynamically stable—one can assume that the formation of the final portion of the branch helix proceeds at a lower pace.

Our simulations also reveal that strand invasion can proceed bidirectionally (Fig. 10 and Supplementary Fig. S8), suggesting a dynamic interplay of base-pair exchange along both the 5′- and 3′-directions of the branch helix. The unidirectional strand displacement observed by cryo-EM might be due to the constraints imposed by the spliceostatin A, which occupies the BPA binding pocket, therefore acting as an obstacle preventing the bidirectional strand invasion [18].

To fully hybridize with the intron, the BSL loop might need to temporarily detach from the SF3B1 surface. Briefly, the 5′-end of U2 snRNA, containing SL I, must unwind and pass between the BSL 3′-end and SF3B1, while the intron, in turn, must undergo the opposite movement to be accommodated in the narrow space between the U2 snRNA and SF3B1 (Fig. 11 and Supplementary Fig. S23). This non-trivial rearrangement is expected to further slow down the final steps of branch helix formation. However, since the BSL and SL I are very flexible, it is possible that, after TAT-SF1 removal, they may dissociate from SF3B1 to allow for the BSL/intron strand exchange before the newly formed branch helix docks back onto the SF3B1 (Supplementary Fig. S23).

Regarding the sequence conservation, the toehold binds to the highly variable BS sequence from human intron “nyUnAy” (Fig. 1) [28]. It is striking that the −2 uracil of the toehold-complementary “nyU” motif is largely invariant—a feature observed only for the branch point adenosine itself. This conservation might derive from its key role in the toehold usage, and the requirement to stabilize the helical geometry adjacent to the bulged adenosine (Supplementary Fig. S24). The −3 position can host a C or U, able to pair with the BSL via canonical G:C or wobble G:U pairs, respectively. This base pair appears to be essential for forming a stable toehold-intron contact, as also suggested by our simulations (Supplementary Fig. S25). Position −4 can be occupied by any base. This could be explained by an intra-BSL base pairing to pseudouracil Ψ37, which proved to be a versatile base, able to pair with all four canonical bases [72]. The base pairing involving the degenerate base at position −4 seems to be further stabilized by the Ψ37/Ψ34 interaction network (Fig. 9). Overall, despite its high degeneracy, the branch site motif in humans retains sufficient conservation to allow base pairing to the BSL’s toehold, thus contributing to ensuring recognition specificity.

In contrast to metazoans, the branch site motif in baker’s yeast shows limited sequence degeneracy and strong complementarity to U2. In yeast, removal of Cus2 (the TAT-SF1 homolog) by Prp5 is normally required for efficient splicing and viability. However, deletion of Cus2 or disruption of its interaction with SF3b1 still permits A complex formation without the need for active Prp5 [24]. These observations suggest that, in yeast, intrinsic base-pairing complementarity is sufficient to drive branch helix formation, reducing the need for a high-energy intermediate imposed by Cus2/TAT-SF1. Consistent with the relatively small number of introns and their conserved branch sites, yeast spliceosomes may therefore rely less on a “loaded spring” mechanism that, in metazoans, could facilitate splicing across a broader range of sequence variability.

The mechanistic picture collectively provided by our simulations also aligns with the kinetic proofreading model, which was proposed upon capturing the U2/SF3b complex in the absence of both TAT-SF1 and the intron, where the U2 snRNA was restructured [67]. Namely, the proofreading, underlying BS selection, has been suggested to occur via a kinetic competition between intron binding and large-scale restructuring of the U2 snRNA. The first path leads to the progression of the splicing cycle, while the latter leads to the formation of an inactive state, which arrests progression. The competition is triggered by TAT-SF1 removal, which releases the BSL’s structural tension, leading to BSL restructuring. Thus, the BSL conformational switch observed here enables the quick formation of an initial short branch helix precursor, promoting the binding of suitable introns. Otherwise, the formation of an inactive state and intron rejection will be promoted [67].

The branch site is initially bound by SF1, a protein indirectly connected to the polypyrimidine tract and the 3′ splice site through its interactions with the U2AF complex [97]. SF1 is particularly important for the splicing of introns with weaker branch sequences [98, 99]. The mechanism of transfer of the branch site from this early complex to the U2 snRNP is unclear. However based on available structural information, the branch point adenosine can initially be buried within a binding pocket of SF1, while adjacent nucleotides remain partially solvent-exposed and thus accessible to U2 snRNA (Supplementary Fig. S26) [98]. This is consistent with our model in which the U2 toehold first engages these flanking nucleotides (positions −4 to −2), prior to incorporation of the BPA into the branch helix (Fig. 1D′) [18]. We propose that the SF1–U2AF complex facilitates positioning of the U2 snRNP in proximity to the branch site without rigidly constraining it, thereby allowing the U2 BSL to sample nearby sequences and engage the optimal site through strand invasion [100]. This scanning may be facilitated by ATP-dependent translocation along the intron, potentially mediated by DHX15/PRP43—a splicing helicase physically associated with the U2 snRNP [18, 100, 101]. Once intron-U2 pairing at suitable positions −4 to −2 is established, SF1 can be displaced from the intron, either due to strand invasion alone or along with helicase-driven remodelling (see also Supplementary Fig. S26).

Overall, we reveal that the intrinsic flexibility of the BSL, owing to non-canonical base pairing of the stem, is crucial for its function. BSL plasticity indeed facilitates its conformational remodelling. This mechanism is most likely shared by other biological systems that leverage intrinsic RNA flexibility to enhance the strand invasion. Indeed, the presence of non-canonical, flexible base-pairs was recently demonstrated to enhance strand displacement in riboswitches as well [85, 102, 103].

Finally, this work underscores the power of molecular simulations to complement cryo-EM structural data and capture transitions, corresponding to short-lived intermediates that are difficult to access in purified complexes. By revealing the dynamic and energetic underpinnings of U2 snRNA strand invasion, our study identifies the loaded-spring mechanism as critical for branch site recognition, and suggests that it may represent a more general paradigm for understanding how RNA elements harness internal strain to drive conformational remodelling in complex RNP assemblies.

## Supplementary Material

gkag429_Supplemental_File

## Data Availability

All simulation input files, derived parameter files, reference structures, and analysis scripts are available in the SI, at https://github.com/ppokor/U2_BH, and at the Zenodo database (DOI: 10.5281/zenodo.19332257).
