# Supplementary material for: U2 snRNP recognizes the branch site through a loaded-spring strand-invasion mechanism
Source: Nucleic Acids Res. 2026 May 13;54(9):gkag429. doi: 10.1093/nar/gkag429 (PMC13167188; doi:10.1093/nar/gkag429)
Supplement: gkag429_Supplemental_File [file gkag429_supplemental_file.pdf]

Supplementary Information for

# U2 snRNP recognizes the branch site through a loaded-spring strand-invasion mechanism

Pavlína Pokorná,[1] Vladimir Pena,[2] and Alessandra Magistrato[1,*]

[1] CNR-IOM at SISSA, via Bonomea 265, 34136, Trieste, Italy
[2] The Institute of Cancer Research, 123 Old Brompton Road, SW7 3RP, London, United Kingdom
[*] corresponding author, e-mail: alema@sissa.it

Supplementary Methods - Metadynamics simulations........2
Supplementary Tables........4
Supplementary Figures........12
Supplementary References........47

## Supplementary Methods - Metadynamics simulations

Below we list the wall restraints used in the metadynamics simulations, see also Figure S1 and the PLUMED input files. Notably, test metadynamics simulations performed with additional walls did not improve the sampling of BSL-branch helix formation events, but induced spurious restructuring in the non-biased segment of the BSL loop instead.

**Metadynamics simulations with an intron having partial complementarity to BSL, replica 1:**

1. upper wall with harmonic restraints on X-H…Y H-bond (hydrogen-acceptor distance) for all H-bonds of the four G=C pairs closing the construct (see the main text for the scheme of the construct): distance of 0.22 nm, force constant of 150 kJ/mol
2. upper wall with harmonic distance restraint on the center-of-mass of the A-U pairs adjacent to the biased nucleotides: distance of 2.0 nm, force constant of 150 kJ/mol
3. pseudodihedral of $C_{-7}$(C4')-$A_{-6}$(P)-$A_{-6}$(C4')-$U_{-5}$(P): lower wall with harmonic restraint on sin(pseudodihedral): sin of 0.0, force constant of 150 kJ/mol
4. upper wall with harmonic restraint on cumulative εRMSD for both target regions: sum(εRMSD) of 4.0, force constant of 300 kJ/mol
5. lower wall with harmonic restraint on $(\varepsilon RMSD_{BSL} - \varepsilon RMSD_{BH})^2 - \min(\varepsilon RMSD_{BSL}, \varepsilon RMSD_{BH})^2$, value of -2.0 force constant of 300 kJ/mol. This setting prevents sampling of regions where both εRMSD values are large.

**Metadynamics simulations with an intron having partial complementarity to BSL, replica 2:**

1., 2., 4., 5., as defined above

3. a set of walls with harmonic restraints on pseudodihedrals, all with a force constant of 150 kJ/mol:

    $C_{-7}$(C4')-$A_{-6}$(P)-$A_{-6}$(C4')-$U_{-5}$(P): lower wall on sin(pseudodihedral): sin of 0.0
    $A_{-6}$(P)-$A_{-6}$(C4')-$U_{-5}$(P)-$U_{-5}$(C4'): upper wall on sin(pseudodihedral): sin of 0.0
    $A_{-6}$(C4')-$U_{-5}$(P)-$U_{-5}$(C4')-$A_{-4}$(P): lower wall on sin(pseudodihedral): sin of 0.0
    $U_{-5}$(P)-$U_{-5}$(C4')-$A_{-4}$(P)-$A_{-4}$(C4'): upper wall on sin(pseudodihedral): sin of 0.0

**Metadynamics simulations with an intron having full complementarity to BSL, replica 1:**

1., 2., 4., 5., as defined above

3. a set of walls with harmonic restraints on pseudodihedrals, all with a force constant of 300 kj/mol:

    $G_{-7}$(C4')-$A_{-6}$(P)-$A_{-6}$(C4')-$U_{-5}$(P): lower wall on sin(pseudodihedral): sin of 0.0; upper wall on sin(pseudodihedral): sin of 0.8
    $A_{-6}$(P)-$A_{-6}$(C4')-$U_{-5}$(P)-$U_{-5}$(C4'): upper wall on sin(pseudodihedral): sin of 0.0
    $A_{-6}$(C4')-$U_{-5}$(P)-$U_{-5}$(C4')-$A_{-4}$(P): lower wall on sin(pseudodihedral): sin of 0.0; upper wall on sin(pseudodihedral): sin of 0.8
    $U_{-5}$(P)-$U_{-5}$(C4')-$A_{-4}$(P)-$A_{-4}$(C4'): upper wall on sin(pseudodihedral): sin of 0.0
    $U_{-5}$(C4')-$A_{-4}$(P)-$A_{-4}$(C4')-$C_{-3}$(P): lower wall on sin(pseudodihedral): sin of −0.2; upper wall on sin(pseudodihedral): sin of 0.8

**Metadynamics simulations with an intron having full complementarity to BSL, replica 2:**

1., 2., 4., 5., as defined above

3. a set of walls with harmonic restraints on pseudodihedrals, all with a force constant of 300 kJ/mol:

$G_{-7}$(C4')-$A_{-6}$(P)-$A_{-6}$(C4')-$U_{-5}$(P): lower wall on sin(pseudodihedral): sin of 0.0; upper wall on sin(pseudodihedral): sin of 0.8

$A_{-6}$(P)-$A_{-6}$(C4')-$U_{-5}$(P)-$U_{-5}$(C4'): upper wall on sin(pseudodihedral): sin of 0.0

$A_{-6}$(C4')-$U_{-5}$(P)-$U_{-5}$(C4')-$A_{-4}$(P): lower wall on sin(pseudodihedral): sin of 0.0; upper wall on sin(pseudodihedral): sin of 0.8

$U_{-5}$(P)-$U_{-5}$(C4')-$A_{-4}$(P)-$A_{-4}$(C4'): upper wall on sin(pseudodihedral): sin of −0.2; lower wall on sin(pseudodihedral): sin of -0.2

$U_{-5}$(C4')-$A_{-4}$(P)-$A_{-4}$(C4')-$C_{-3}$(P): lower wall on sin(pseudodihedral): sin of −0.2; upper wall on sin(pseudodihedral): sin of 0.8

## Supplementary Tables

**Table S1:** Analysis of $K^+$ ion coordination to RNA atoms over molecular dynamics simulation trajectories. Population is computed as the number of frames where the coordination is present relative to the total number of frames in the trajectory.

**$K^+$ bridges in the toehold+loop region** with population >= 0.05%[a]

**MINX intron, standard FF**

| Residues | Population (%) |
|---|---|
| U32:G33:U-5 | 0.08 |
| A38:U39:U-5 | 0.06 |

**Fully complementary intron, standard FF**

| Residues | Population (%) |
|---|---|
| A35:U-2:A-1[b] | 0.11 |
| U34:A35:G36:U-2 | 0.07 |

**$K^+$ coordination** with population >= 30%[c]

**MINX intron, standard FF**

| Residue@atom | Population (%) |
|---|---|
| G33@O6 | 0.66 |
| G42@O6 | 0.58 |
| G36@O6 | 0.55 |
| U43@O4 | 0.50 |
| U34@O4 | 0.48 |
| A30@N7 | 0.48 |
| U32@O4 | 0.46 |
| C-7@O2 | 0.46 |
| U27@O4 | 0.38 |
| G-9@O6 | 0.36 |
| G31@N7 | 0.36 |
| A35@N7 | 0.35 |
| G36@N7 | 0.35 |
| U34@O2' | 0.34 |
| C-7@N3 | 0.30 |

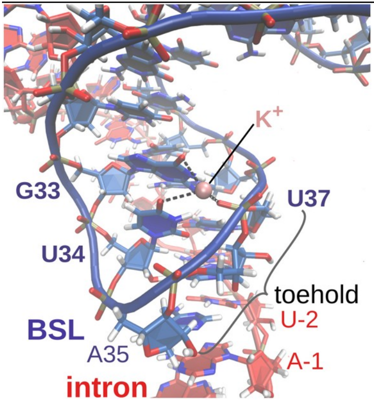


**Fully complementary intron, standard FF**

| Residue@atom | Population (%) |
|---|---|
| G33@O6 | 0.73 |
| G-7@O6 | 0.64 |
| G42@O6 | 0.60 |
| U43@O4 | 0.59 |
| U32@O4 | 0.58 |
| G-7@N7 | 0.54 |
| U34@O4 | 0.52 |
| G31@O6 | 0.50 |
| A-8@N7 | 0.49 |
| A-8@OP2 | 0.45 |
| A35@N7 | 0.44 |
| C-9@OP2 | 0.43 |
| U41@O4 | 0.42 |
| C+3@O2 | 0.40 |
| U-5@O4 | 0.36 |
| A30@N7 | 0.35 |
| G33@N7 | 0.35 |
| A38@OP2 | 0.34 |
| U27@O4 | 0.34 |
| U34@O2' | 0.34 |
| A-1@N7 | 0.33 |
| A26@OP2 | 0.32 |

[a] calculated with Cpptraj, using a distance cutoff of 4.0 Å and no angle cutoff.

[b] associated mostly with non-canonical A11:U-2 base pairing.

[c] calculated with Cpptraj, using a distance cutoff of 3.0 Å and no angle cutoff. For the BSL stem and the short branch helix segment, the coordinating atoms and populations correspond to expected values for an A-RNA helix. The inset shows an example snapshot with the loop's G33@O6 and U34@O4 coordinating $K^+$.

**Table S2:** List of molecular dynamics simulations performed.

| Force field modifications | Number of replicas | Range of replica lengths (μs) |
|---|---|---|
| **Intron-BSL RNA construct, intron with partial complementarity** | | |
| - | 10 | 0.5-1.0 |
| HBfix | 10 | 1.0-1.5 |
| HBfix, stafix | 10 | 0.5-1.7 |
| **Intron-BSL RNA construct, intron with full complementarity** | | |
| - | 10 | 0.5-1.0 |
| HBfix | 10 | 0.5-1.0 |
| HBfix, stafix | 10 | 0.5-1.5 |
| **Intron-BSL RNA construct, intron with partial complementarity to BSL, A-6U** | | |
| - | 10 | 1.0 |
| HBfix | 10 | 0.5 |
| HBfix, stafix | 10 | 0.5 |
| **Intron-canonical BSL RNA construct, intron with partial complementarity to BSL** | | |
| - | 10 | 1.0 |
| HBfix | 10 | 0.5 |
| HBfix, stafix | 10 | 0.5 |
| **Intron-canonical BSL RNA construct, intron with partial complementarity to BSL, A-6U** | | |
| - | 10 | 1.0 |
| HBfix | 10 | 0.5 |
| HBfix, stafix | 10 | 0.5 |
| **Intron-BSL RNA construct, intron with partial complementarity to BSL and with pseudouridines** | | |
| - | 10 | 1.0 |
| **Small intron-BSL RNA construct, intron substitutions in positions -3 to -5 (SI only)** | | |
| - | 100 | 0.5 |
| **U2 17S construct** | | |
| - | 2 | 2.0 |
| **U2 17S construct without TAT-SF1** | | |
| - | 4 | 2.0 |
| **Metadynamics, intron with partial complementarity** | | |
| - | 2 | 3.0-6.0 |

| | | |
|---|---|---|
| **Metadynamics, intron with full complementarity** | | |
| - | 2 | 1.0-5.2 |
| **Metadynamics, intron with partial complementarity, distance CV (SI only)** | | |
| - | 1 | 7.75 |
| **intron-BSL RNA construct, intron with full complementarity** | | |
| coarse grained | 100 | $25 \times 10^6$ steps |

**Table S3:** Restructuring events leading to branch helix formation in the molecular dynamics simulations of the complete RNA model performed with different Amber force field variants. Simulations which did not sample any new branch helix base pairs are not listed.

| Simulation force field | Length (μs) | 1st BP formed (ns) | 2nd BP formed (ns) | 3rd BP formed (ns) | 4th BP formed (ns) |
|---|---|---|---|---|---|
| **Intron-BSL RNA construct, intron with partial complementarity to BSL** | | | | | |
| standard | 1.0 | 393 | | | |
| standard | 0.5 | 47 | 69 | | |
| standard | 0.5 | 31 | | | |
| HBfix | 1.5 | 174 | | | |
| HBfix | 1.5 | 136 | | | |
| HBfix | 1.5 | | 498 | 723 | |
| HBfix | 1.5 | 107 | 776 | 266 | |
| HBfix, stafix | 1.7 | 97 | 102 | 128 | |
| HBfix, stafix | 0.5 | 20 | | 125 | |
| HBfix, stafix | 0.5 | | 379 | | |
| HBfix, stafix | 2.0 | 372 | 447 | | |
| **Intron-BSL RNA construct, intron with full complementarity to BSL** | | | | | |
| standard | 1.0 | 100 | | | |
| standard | 1.0 | 248 | 264 | | |
| standard | 1.0 | 886 | | | |
| standard | 1.0 | 236 | | | |
| standard | 1.0 | 59 | 90 | | |
| HBfix | 1.0 | 341 | 395 | | |
| HBfix | 1.0 | 61 | 64 | | |
| HBfix | 1.0 | 92 | 86 | | |
| HBfix | 1.0 | 76 | 326 | | |
| HBfix | 1.0 | 123 | 186 | | |
| HBfix | 1.0 | 107 | 119 | 228 | |
| HBfix | 1.0 | 50 | | 55 | |
| HBfix, stafix | 1.0 | 22 | 209 | 14 | 249 |
| HBfix, stafix | 1.0 | 352 | 352 | | |
| HBfix, stafix | 1.0 | 127 | 151 | 211 | |
| HBfix, stafix | 0.5 | 32 | 103 | | |
| HBfix, stafix | 1.5 | 76 | 131 | 275 | 342 |
| **Intron-idealized (canonical) BSL RNA construct, intron with partial complementarity to BSL** | | | | | |

| | | | | |
|---|---|---|---|---|
| standard | 1.0 | 219 | 219 | |
| standard | 1.0 | 49 | | |
| standard | 1.0 | 199 | 439 | |
| standard | 1.0 | 271 | | |
| HBfix | 0.5 | 223 | 311 | |
| HBfix | 0.5 | 31 | | |
| HBfix | 0.5 | 35 | 41 | |
| HBfix | 0.5 | 98 | 140 | |
| HBfix | 0.5 | 21 | | |
| HBfix, stafix | 0.5 | 38 | 45 | |
| HBfix, stafix | 0.5 | 82 | 290 | |
| HBfix, stafix | 0.5 | 382 | 402 | |
| HBfix, stafix | 0.5 | 490 | | |
| **Intron-BSL RNA construct, intron with partial complementarity to BSL, A-6U** | | | | |
| standard | 1.0 | 852 | | |
| HBfix | 0.5 | 209 | | |
| HBfix | 0.5 | 99 | 104 | |
| HBfix | 0.5 | 482 | 482 | |
| HBfix | 0.5 | 18 | 18 | |
| HBfix | 0.5 | 272 | 273 | 482 |
| HBfix, stafix | 0.5 | 21 | | |
| HBfix, stafix | 0.5 | 52 | 52 | |
| HBfix, stafix | 0.5 | 20 | 20 | 453 |
| HBfix, stafix | 0.5 | 37 | 39 | 374 |
| HBfix, stafix | 0.5 | 103 | 116 | 370 |
| HBfix, stafix | 0.5 | 40 | 47 | |
| HBfix, stafix | 0.5 | 492 | 498 | |
| **Intron-idealized (canonical) BSL RNA construct, intron with partial complementarity to BSL, A-6U** | | | | |
| standard | 1.0 | 410 | | |
| HBfix | 0.5 | 14 | 27 | |
| HBfix | 0.5 | 145 | | |
| HBfix | 0.5 | 9 | 10 | |
| HBfix | 0.5 | 28 | 30 | |
| HBfix | 0.5 | 113 | 225 | |

| | | | | |
|---|---|---|---|---|
| HBfix, stafix | 0.5 | 153 | 153 | |
| HBfix, stafix | 0.5 | 98 | | |
| HBfix | 0.5 | 443 | 445 | |
| HBfix, stafix | 0.5 | 222 | | |
| HBfix, stafix | 0.5 | 72 | 77 | |
| **Intron-BSL RNA construct, intron with partial complementarity to BSL and with pseudouracils** | | | | |
| standard | 0.5 | 138 | | |
| standard | 0.5 | 48 | | |
| standard | 0.5 | 58 | | 144 |

**Table S4:** RNA-protein BSL/TAT-SF1 interactions evaluated with Key Interaction Finder [1] on a merged trajectory of the U2/SF3b complex with TAT-SF1. The first 400 ns of each trajectory are not included in the analysis. Occupancy is computed as the number of frames where the coordination is present relative to the total number of frames in the trajectory.

| RNA residue | BSL region | Protein residue | TAT-SF1 domain | Type | Residue segment[a] | Average strength (internal KIF units) | Occupancy (%) |
|---|---|---|---|---|---|---|---|
| G36 | toehold | Arg254 | linker | Hbond | sc-sc | 13.8 | 99.9 |
| A35 | toehold | Arg254 | linker | Hbond | sc-sc | 11.2 | 99.6 |
| A35 | toehold | Ser258 | linker | Hbond | sc-sc | 4.8 | 97.2 |
| A35 | toehold | Arg264 | UHM | Hbond | sc-sc | 6.1 | 93.9 |
| A35 | toehold | Arg261 | linker | Hbond | sc-sc | 4.1 | 90.4 |
| U34 | loop | Arg302 | UHM | Hbond | sc-sc | 5.4 | 89.3 |
| G36 | toehold | Arg261 | linker | Hbond | sc-sc | 3.3 | 77.8 |
| A35 | toehold | Glu252 | linker | Hbond | sc-bb | 4.6 | 75.6 |
| G36 | toehold | Arg264 | UHM | Hbond | sc-sc | 3.1 | 75.5 |
| G36 | toehold | Ala255 | linker | Hbond | sc-bb | 1.7 | 74.6 |
| A35 | toehold | Arg259 | linker | Hbond | sc-bb | 3.5 | 71.0 |
| U34 | loop | Arg264 | UHM | Hbond | sc-sc | 4.7 | 63.9 |
| G33 | loop | Arg302 | UHM | Hbond | sc-sc | 3.9 | 60.1 |
| A35 | toehold | Met260 | linker | Hbond | sc-bb | 4.2 | 57.0 |
| A35 | toehold | Ala255 | linker | Hbond | sc-bb | 2.0 | 55.6 |
| U37 | toehold | Arg254 | linker | Hbond | sc-sc | 3.1 | 55.0 |
| A35 | toehold | Arg253 | linker | Other | sc-bb | 3.4 | 54.5 |
| G36 | toehold | Pro251 | linker | Hbond | sc-sc | 2.2 | 54.0 |
| U32 | stem | Arg320 | UHM | Hbond | sc-sc | 3.8 | 50.5 |
| A35 | toehold | Pro251 | UHM | Hbond | sc-bb | 5.3 | 50.0 |
| G33 | loop | Arg320 | UHM | Hbond | sc-sc | 2.9 | 50.0 |
| A35 | toehold | Arg250 | linker | Hbond | sc-sc | 1.2 | 49.8 |
| G36 | toehold | Arg250 | linker | Hbond | sc-sc | 1.7 | 48.8 |
| U34 | loop | Arg320 | UHM | Hbond | sc-bb | 2.9 | 47.6 |
| A35 | toehold | Arg302 | UHM | Hbond | sc-sc | 2.8 | 45.5 |
| G31 | stem | Arg320 | UHM | Hbond | sc-sc | 2.3 | 45.0 |
| U32 | stem | Arg302 | UHM | Hbond | sc-sc | 2.5 | 44.9 |
| C40 | stem | Lys238 | linker α-helix | Hbond | sc-sc | 1.7 | 43.2 |
| U37 | toehold | Ala255 | linker | Hbond | sc-bb | 1.5 | 42.3 |
| U34 | loop | Lys303 | UHM | Hbond | sc-sc | 1.5 | 40.3 |

[a] "sc" stands for side chain, "bb" stands for backbone.

## Supplementary Figures

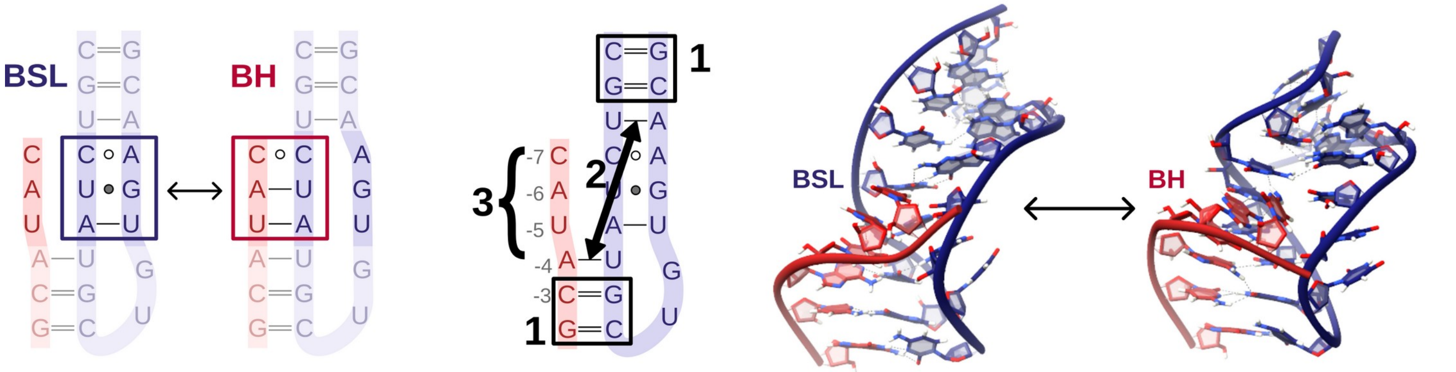


**Figure S1:** RNA model system used in the metadynamics simulations. The left panel shows a cartoon of the model with the intron sequence depicted in red and the BSL in blue. The box denotes the base pairs biased in the metadynamics simulations. The middle panel presents a scheme of some of the applied wall restraints; numbering corresponds to the list reported in Supplementary Results 1. The right panel shows the structures of the target BSL and branch helix (BH) states.

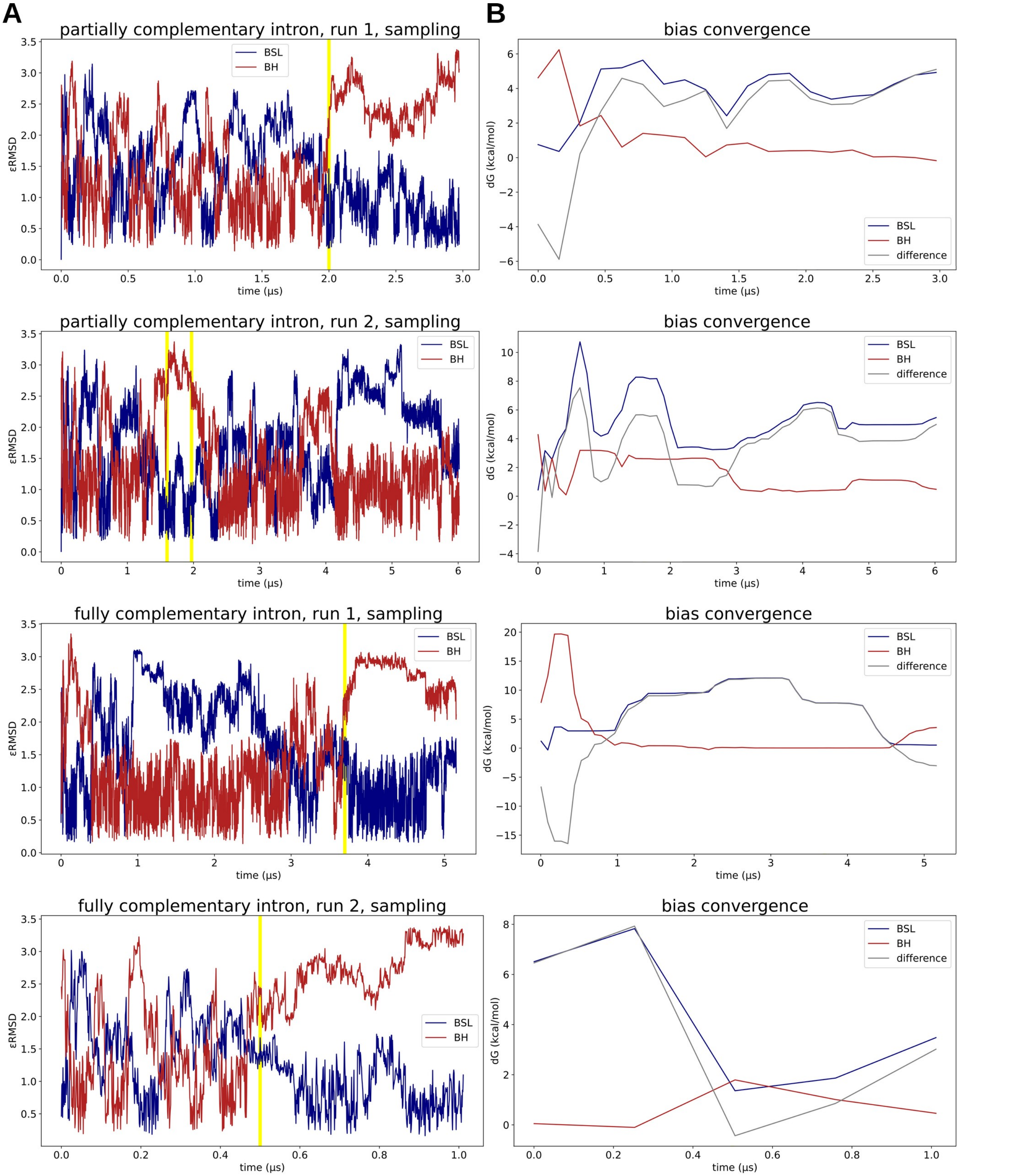


**Figure S2: Progress of metadynamics simulations. (A)** εRMSD as a function of simulation time for the different systems and replicas. The blue and red lines report the εRMSD evolution with respect to the BSL and the branch helix (BH) target structures. The yellow vertical lines mark the time after which (or the interval in which) the simulations were truncated for the analysis. **(B)** Convergence of the non-averaged bias versus simulation time for the different systems and replicas. Note the different time scales in each graph.

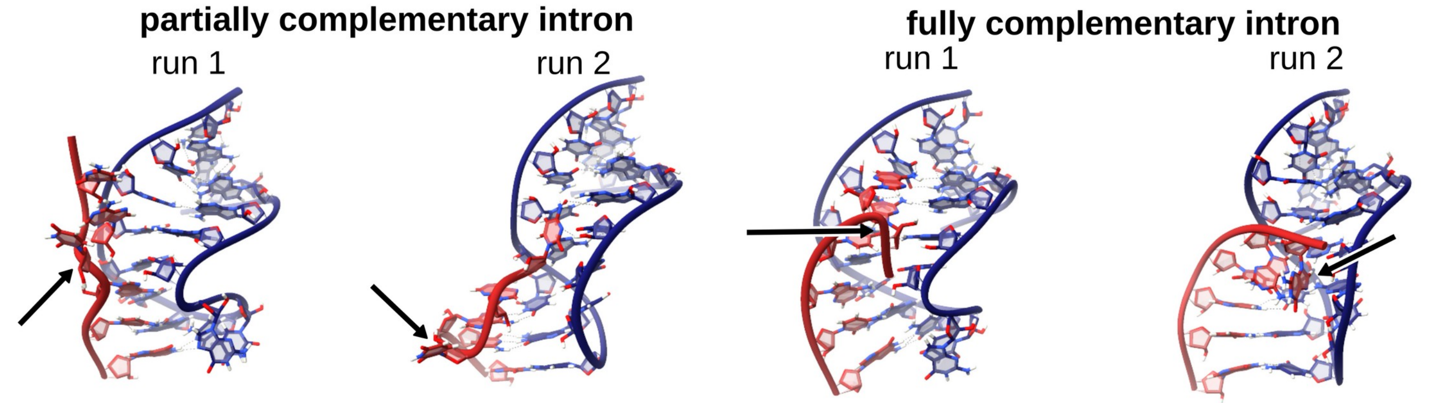


**Figure S3: Off-target states visited in metadynamics simulations of the intron (red ribbons) featuring partial (left) and full (right) base pair complementarity to the BSL (blue ribbons).** The arrows point to problematic interactions. Namely, in the first and second figures, U−5 is bulged out, in the third figure a sharp backbone turn causes G−7 stacking between A−6 and U−5; and in the last figure G−7 is stacked away from the BSL minor groove.

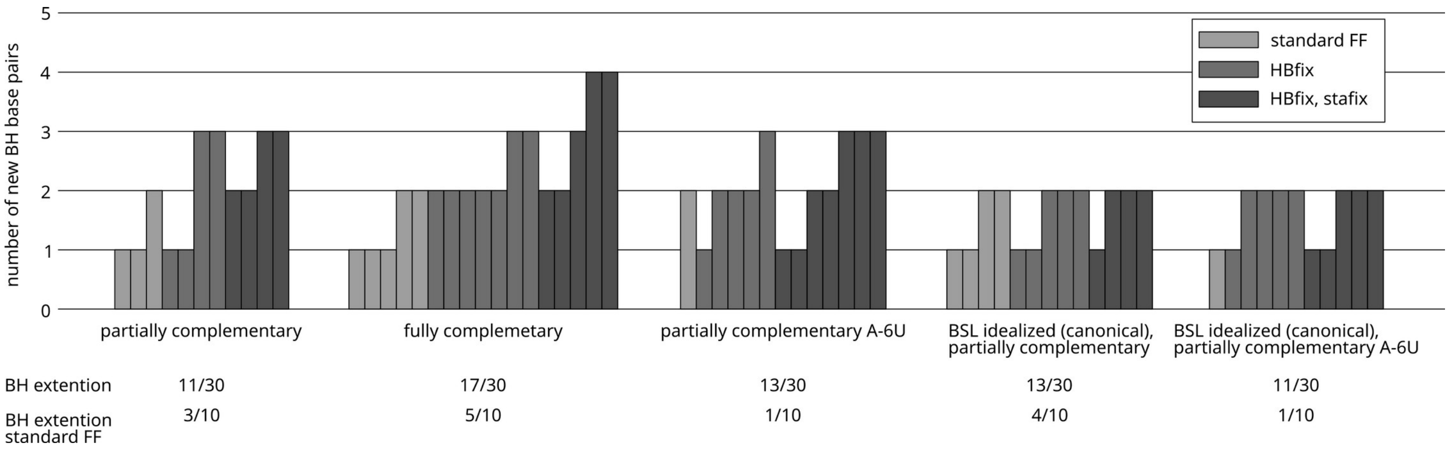


**Figure S4: Sampling of the branch helix (BH) extension in the different intron-BSL simulation sets.** Columns in the graph represent individual replicas which sampled the formation of new branch-helix base pair(s). For each system, 10 MD simulation replicas were run with the standard force field, 10 replicas with the HB-fix FF variant, and 10 replicas with the HB-fix+sta-fix FF variant. Trajectories that did not sample any new branch-helix base pairs are not included in the analysis. The numbers shown below the graphs indicate the number of replicas in which the branch-helix extension of at least one base pair was detected, relative to the total number of replicas.

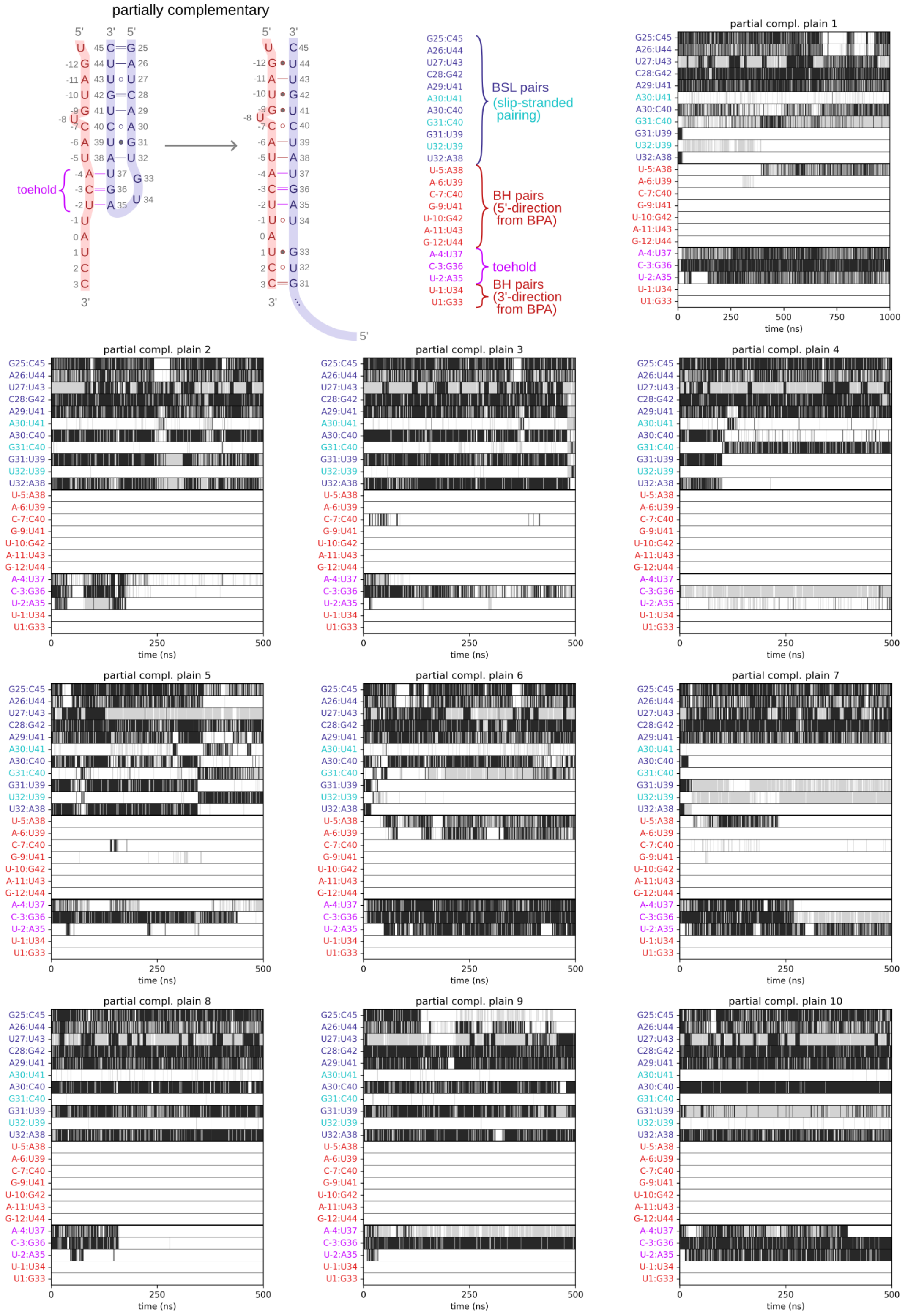

**Figure S5A:** Simulations with the partially complementary intron and no force-field modification. The graphs show the formation of the base pairs versus simulation time in the 10 simulation replicas per system/force-field variant. Black, grey, and white colours indicate the full formation of the base pair (heavy-atom distance < 3.2 Å and angle > 140° for all expected H-bonds), weak pairing (distance < 3.5 Å and angle > 120° for at least one of the expected H-bonds), and the absence of base pairing. The pairs of BSL are labelled in blue, while the hydrogen-bonds of the branch helix are labelled in red, the slip-stranded pairs are cyan and the toehold pairs are violet. A scheme of the simulated system is shown in the upper left corners.

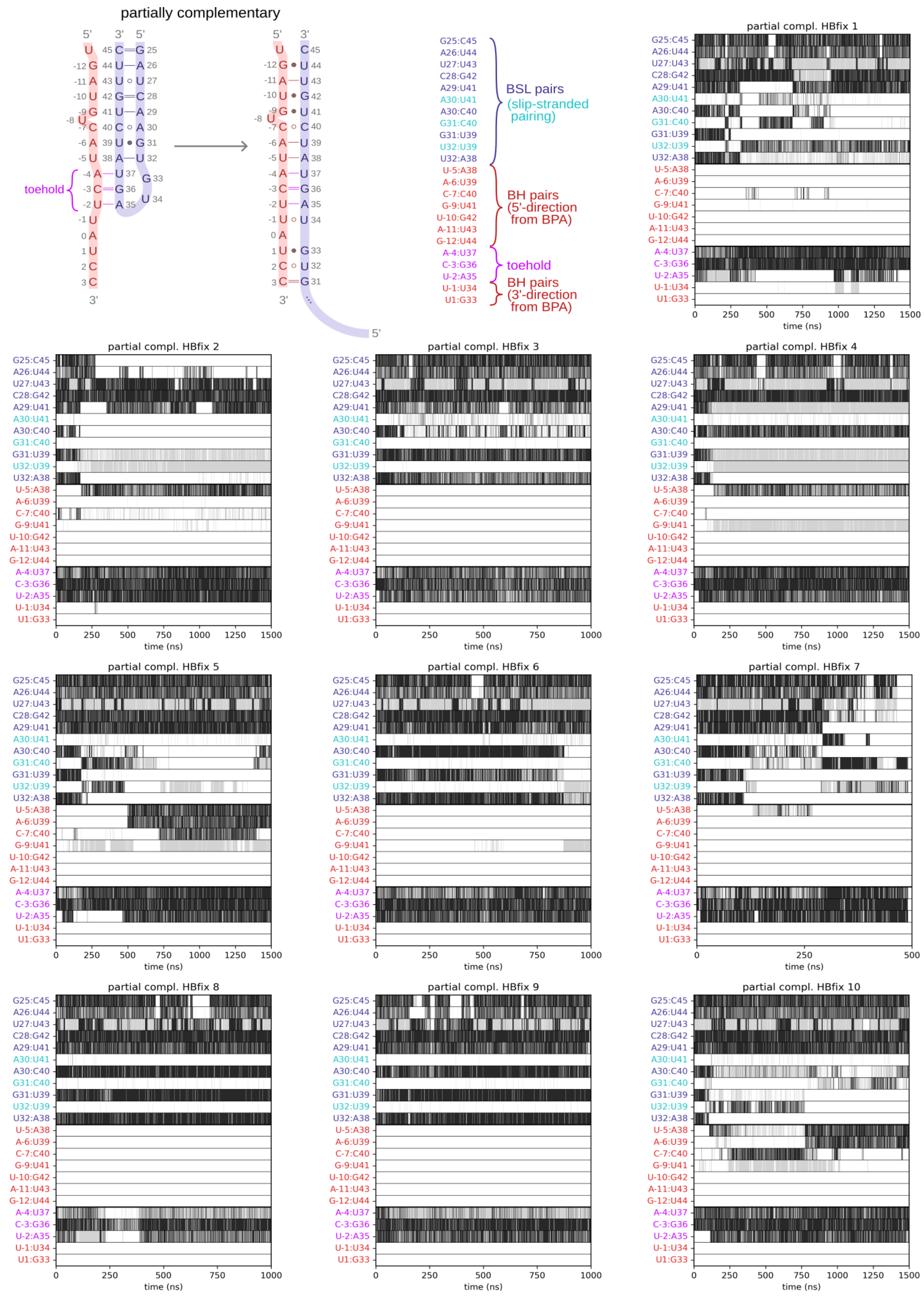


**Figure S5B:** Simulations with the partially complementary intron and HB-fix modification. Run 7 is not counted in the branch helix extension statistics as the helix geometry there is strongly deformed. Black, grey, and white colours indicate the full formation of the base pair (heavy-atom distance < 3.2 Å and angle > 140° for all expected H-bonds), weak pairing (distance < 3.5 Å and angle > 120° for at least one of the expected H-bonds), and the absence of base pairing. The pairs of BSL are labelled in blue, while the hydrogen-bonds of the branch helix are labelled in red, the slip-stranded pairs are cyan and the toehold pairs are violet. A scheme of the simulated system is shown in the upper left corners.

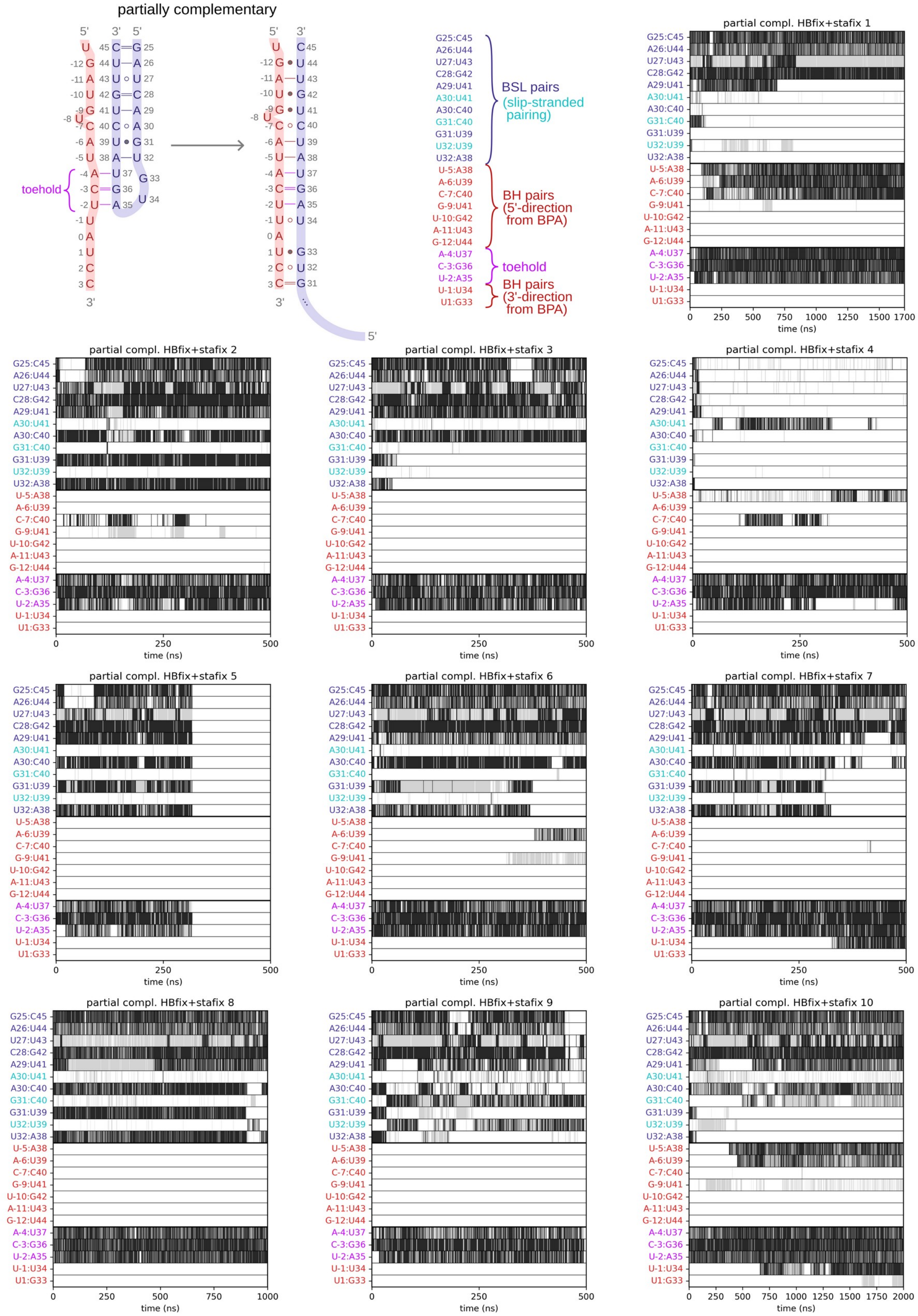


**Figure S5C:** Simulations with the partially complementary intron and HB-fix+sta-fix modifications. Black, grey, and white colours indicate the full formation of the base pair (heavy-atom distance < 3.2 Å and angle > 140° for all expected H-bonds), weak pairing (distance < 3.5 Å and angle > 120° for at least one of the expected H-bonds), and the absence of base pairing. The pairs of BSL are labelled in blue, while the hydrogen-bonds of the branch helix are labelled in red, the slip-stranded pairs are cyan and the toehold pairs are violet. A scheme of the simulated system is shown in the upper left corners.

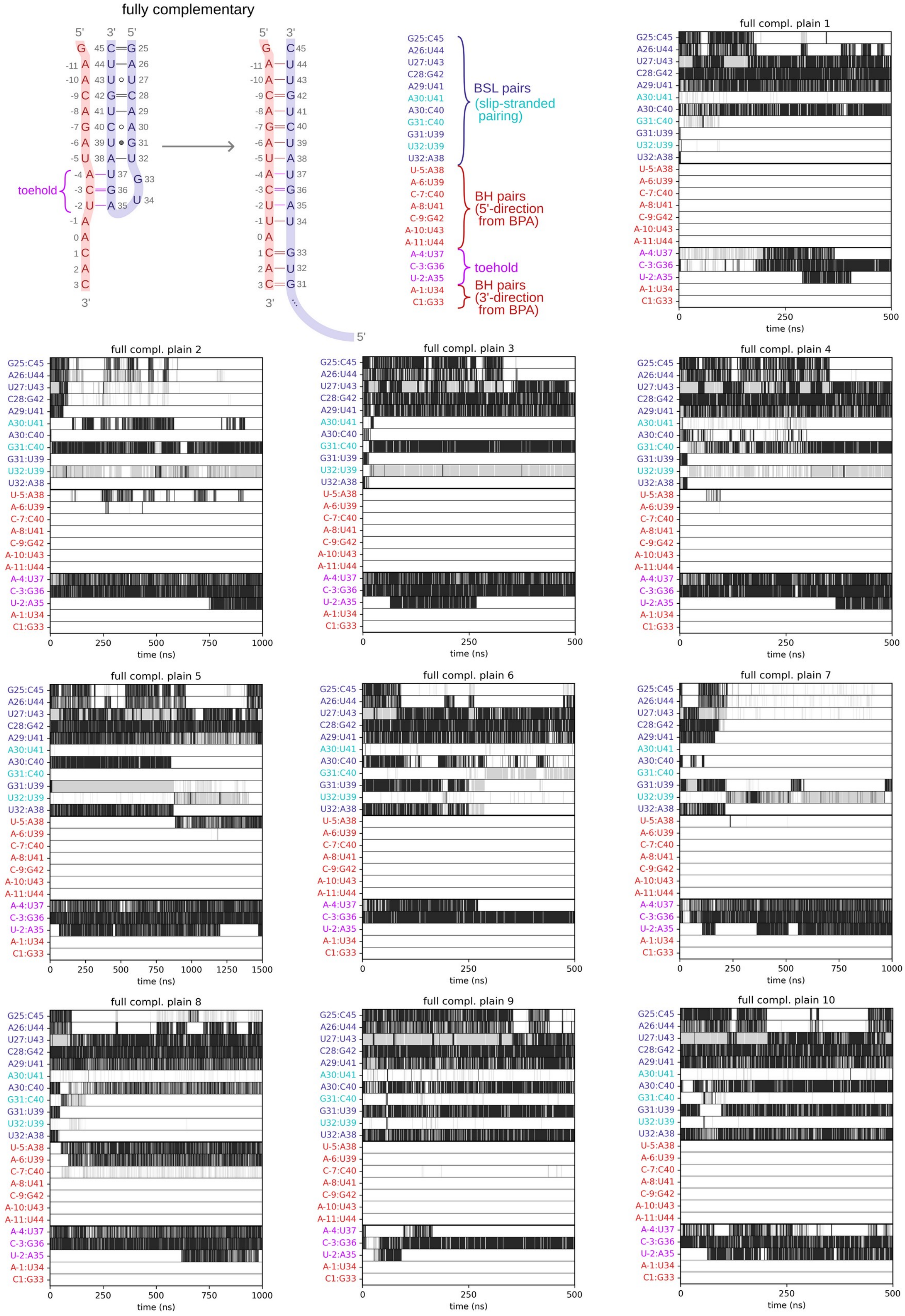


**Figure S6A:** Simulations with the fully complementary intron, and no force-field modification. Black, grey, and white colours indicate the full formation of the base pair (heavy-atom distance < 3.2 Å and angle > 140° for all expected H-bonds), weak pairing (distance < 3.5 Å and angle > 120° for at least one of the expected H-bonds), and the absence of base pairing. The pairs of BSL are labelled in blue, while the hydrogen-bonds of the branch helix are labelled in red, the slip-stranded pairs are cyan and the toehold pairs are violet. A scheme of the simulated system is shown in the upper left corners.

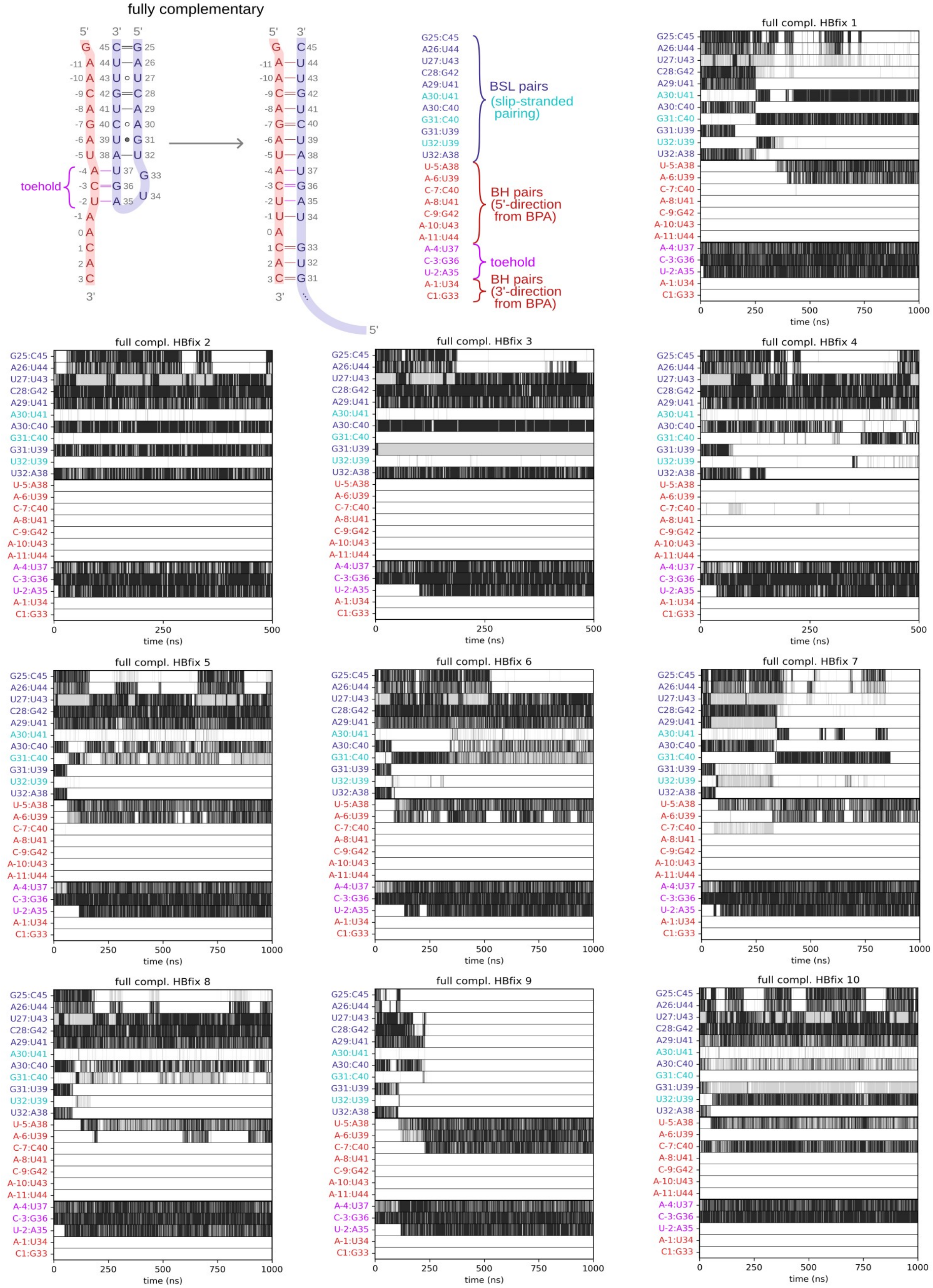


**Figure S6B**: Simulations with the fully complementary intron, and HB-fix modification. Black, grey, and white colours indicate the full formation of the base pair (heavy-atom distance < 3.2 Å and angle > 140° for all expected H-bonds), weak pairing (distance < 3.5 Å and angle > 120° for at least one of the expected H-bonds), and the absence of base pairing. The pairs of BSL are labelled in blue, while the hydrogen-bonds of the branch helix are labelled in red, the slip-stranded pairs are cyan and the toehold pairs are violet. A scheme of the simulated system is shown in the upper left corners.

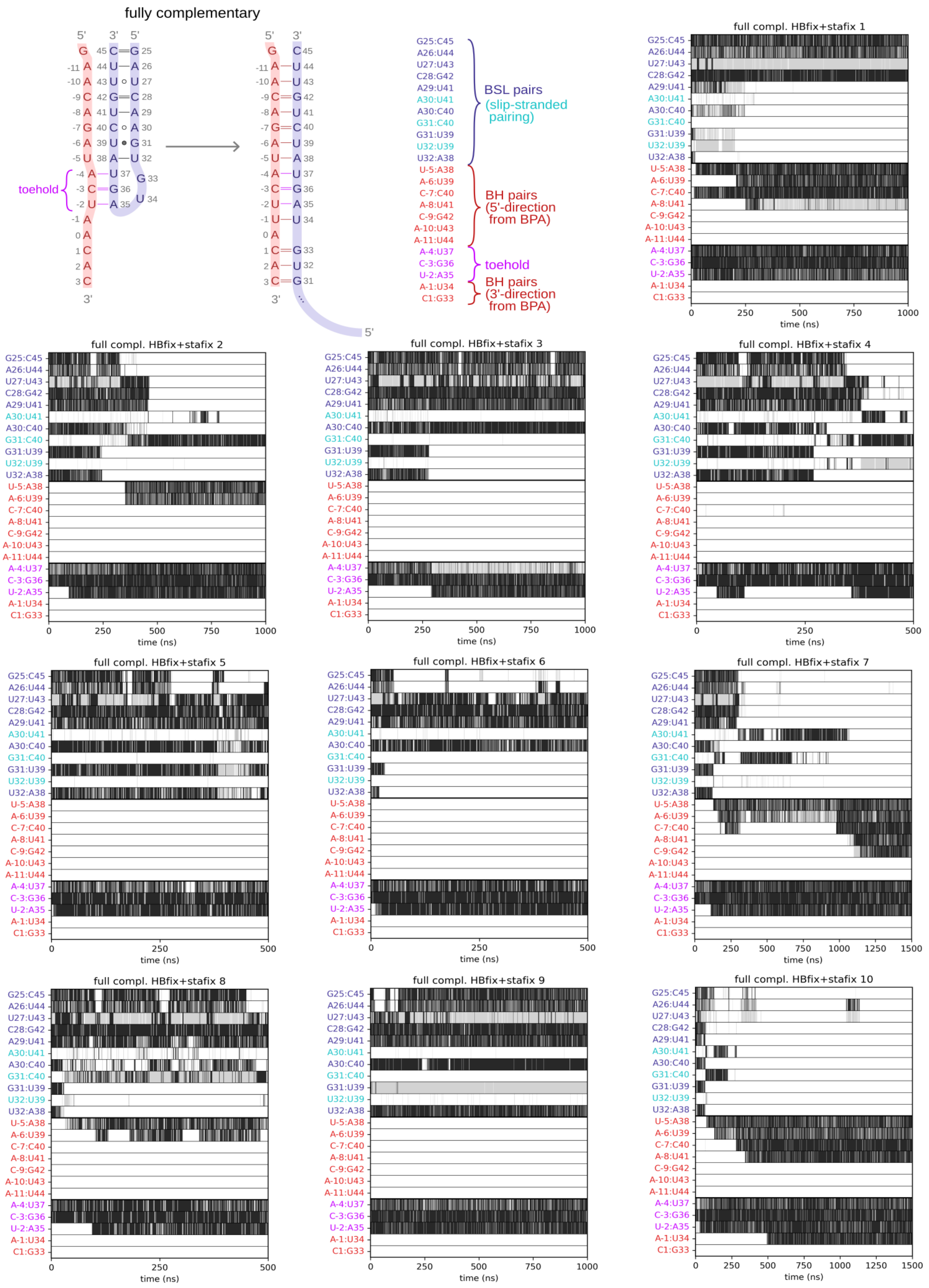


**Figure S6C:** Simulations with the fully complementary intron, and HB-fix+sta-fix modifications. Black, grey, and white colours indicate the full formation of the base pair (heavy-atom distance < 3.2 Å and angle > 140° for all expected H-bonds), weak pairing (distance < 3.5 Å and angle > 120° for at least one of the expected H-bonds), and the absence of base pairing. The pairs of BSL are labelled in blue, while the hydrogen-bonds of the branch helix are labelled in red, the slip-stranded pairs are cyan and the toehold pairs are violet. A scheme of the simulated system is shown in the upper left corners.

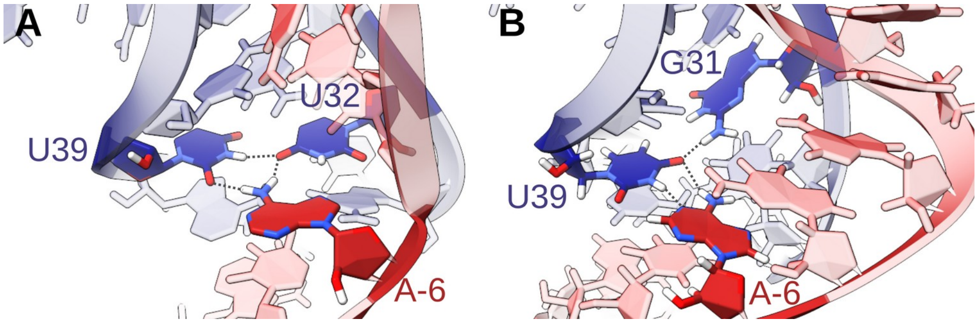


**Figure S7: Examples of base triples formed during the base-pair exchanges in the simulations of the RNA-only constructs. (A)** Transition from the slip-stranded U32:U39 base pairing to the A−6:U39 pair. **(B)** Transition from the non-strand-slipped G31:U39 base pairing to the A−6:U39 pair.

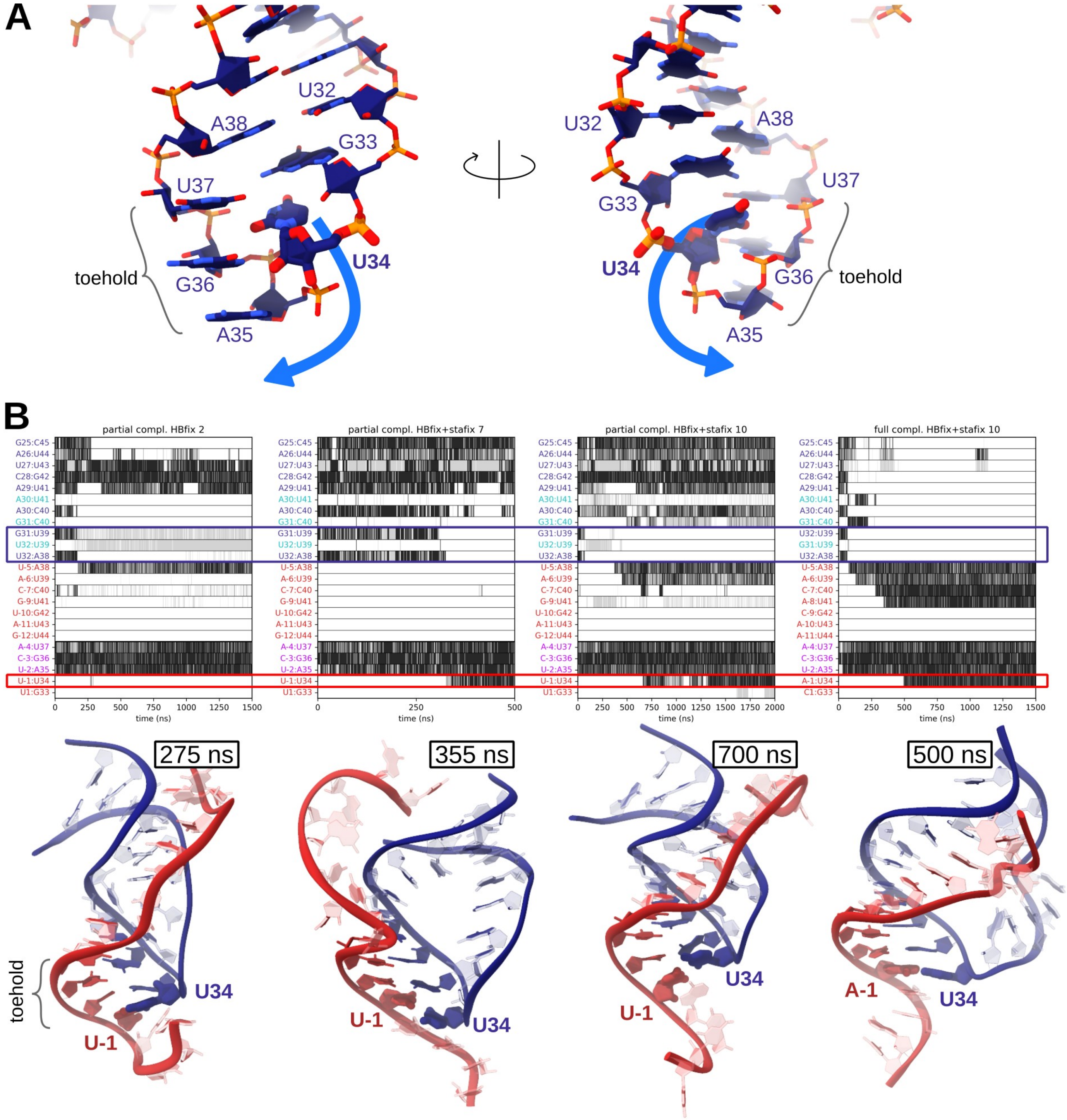


**Figure S8: BSL U34 and the pairing with the −1 residue of the intron. (A)** Description of the experimental structure of the BSL tip (PDB 7EVO), emphasizing the position of U34. The blue arrows indicate the distance U34 would need to relocate below the A35 base to participate in binding to the intron. **(B)** MD simulations which sampled the formation of the U/A−1:U34 base pair, i.e., the branch helix growth in the opposite direction from the toehold pairing. The upper graphs show the development of the base pairing as analysed in Figures S5 and S6; the black, grey, and white colours indicate the full formation of the respective base pair, weak base pairing, and the absence of base pairing. The U/A−1:U34 pair is highlighted by the red box. The structures below show selected snapshots at the onset of formation of the U/A−1:U34 base pair. Notably, in all these trajectories, the BSL pairing adjacent to the toehold (involving U32 and G1, highlighted by a blue box in the graphs) is lost before the U/A−1:U34 pair forms.

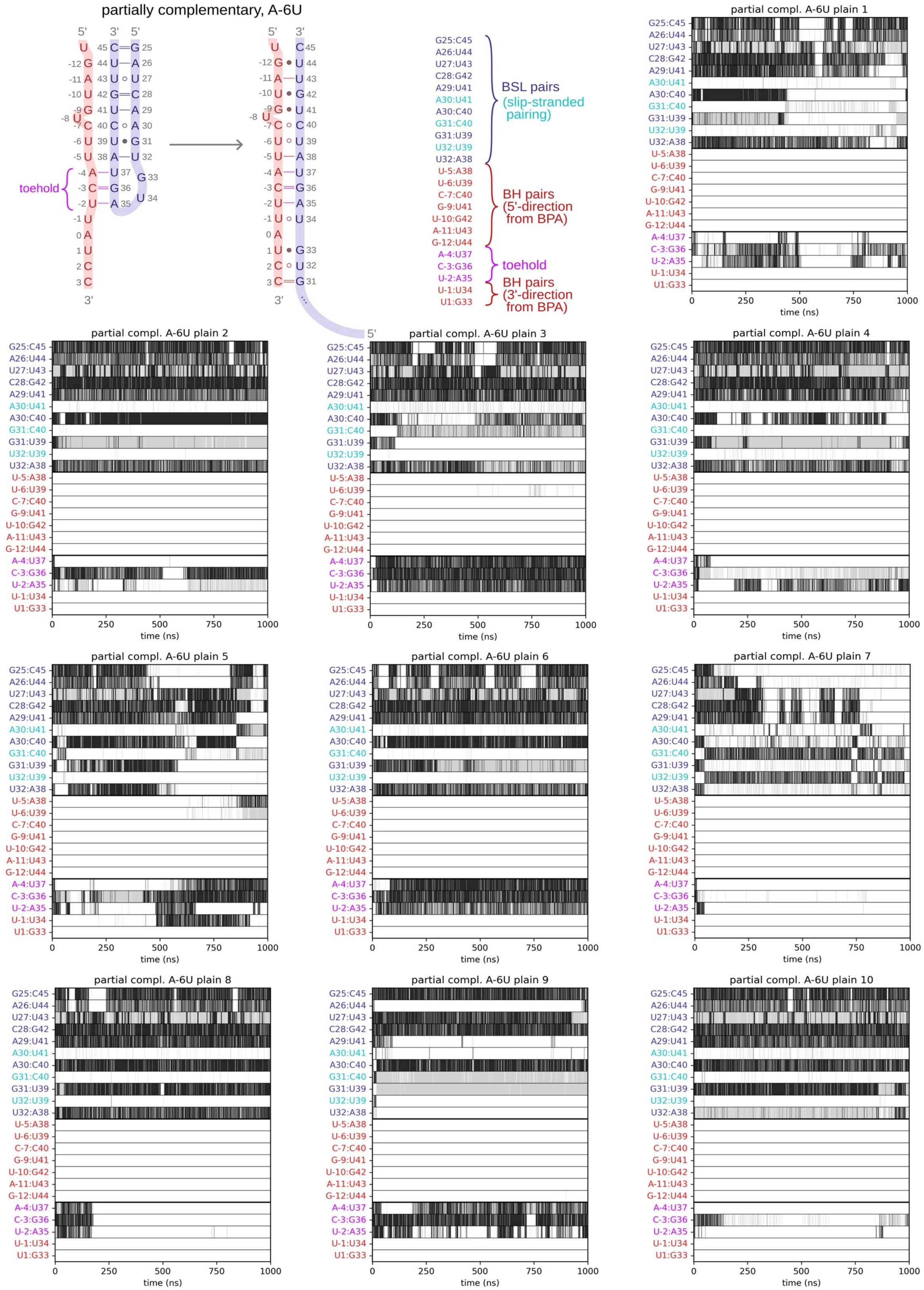


**Figure S9A:** Simulations with the partially complementary intron with A−6U mutation, and no force-field modification. Black, grey, and white colours indicate the full formation of the base pair (heavy-atom distance < 3.2 Å and angle > 140° for all expected H-bonds), weak pairing (distance < 3.5 Å and angle > 120° for at least one of the expected H-bonds), and the absence of base pairing. The pairs of BSL are labelled in blue, while the hydrogen-bonds of the branch helix are labelled in red, the slip-stranded pairs are cyan and the toehold pairs are violet. A scheme of the simulated system is shown in the upper left corners.

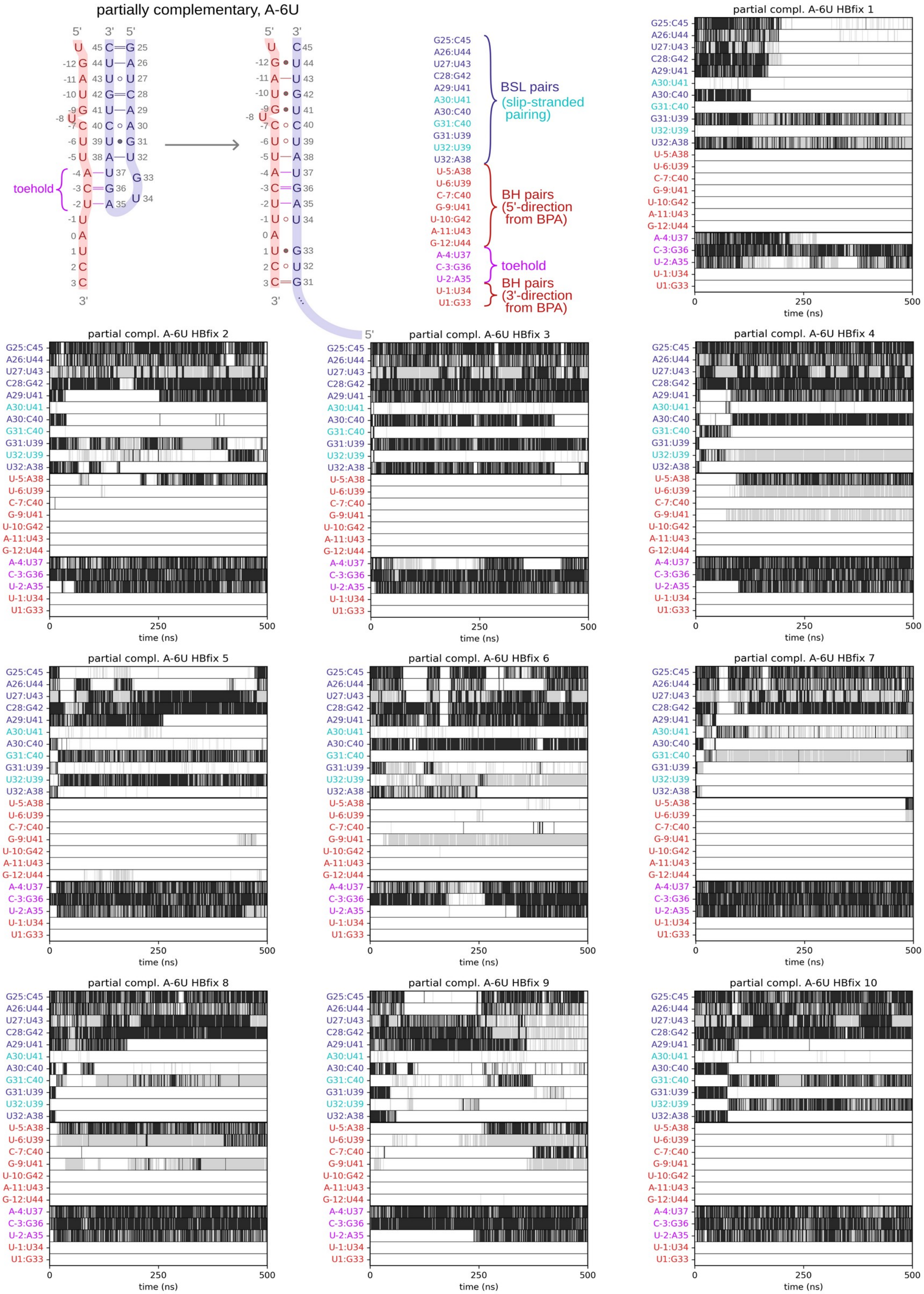


**Figure S9B:** Simulations with the partially complementary intron with A−6U mutation, and HB-fix modification. Black, grey, and white colours indicate the full formation of the base pair (heavy-atom distance < 3.2 Å and angle > 140° for all expected H-bonds), weak pairing (distance < 3.5 Å and angle > 120° for at least one of the expected H-bonds), and the absence of base pairing. The pairs of BSL are labelled in blue, while the hydrogen-bonds of the branch helix are labelled in red, the slip-stranded pairs are cyan and the toehold pairs are violet. A scheme of the simulated system is shown in the upper left corners.

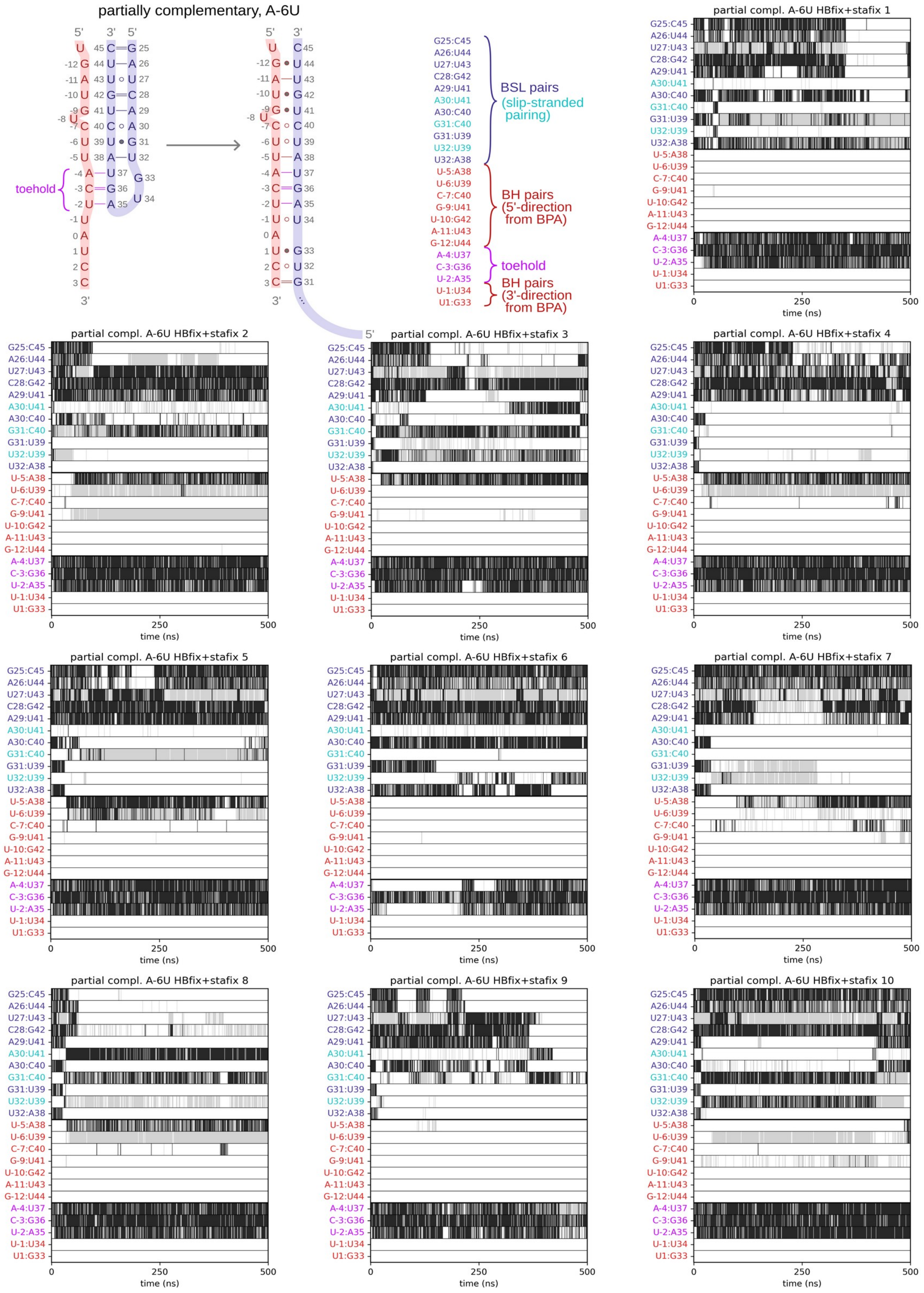


**Figure S9C:** Simulations with the partially complementary intron with A−6U mutation, and HB-fix+sta-fix modifications. Black, grey, and white colours indicate the full formation of the base pair (heavy-atom distance < 3.2 Å and angle > 140° for all expected H-bonds), weak pairing (distance < 3.5 Å and angle > 120° for at least one of the expected H-bonds), and the absence of base pairing. The pairs of BSL are labelled in blue, while the hydrogen-bonds of the branch helix are labelled in red, the slip-stranded pairs are cyan and the toehold pairs are violet. A scheme of the simulated system is shown in the upper left corners.

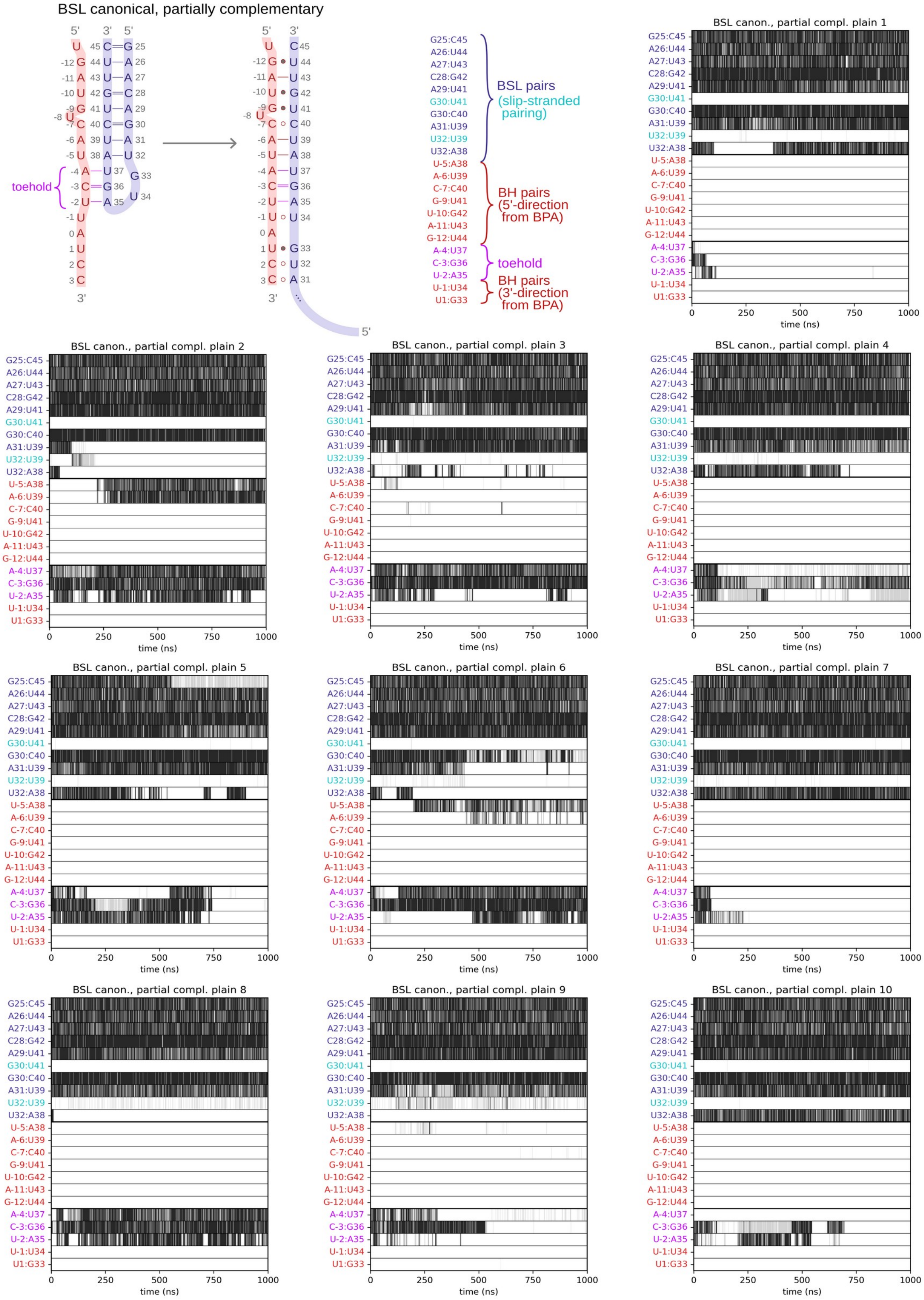


**Figure S10A:** Simulations with the partially complementary intron, idealized canonical BSL, and no force-field modification. Black, grey, and white colours indicate the full formation of the base pair (heavy-atom distance < 3.2 Å and angle > 140° for all expected H-bonds), weak pairing (distance < 3.5 Å and angle > 120° for at least one of the expected H-bonds), and the absence of base pairing. The pairs of BSL are labelled in blue, while the hydrogen-bonds of the branch helix are labelled in red, the slip-stranded pairs are cyan and the toehold pairs are violet. A scheme of the simulated system is shown in the upper left corners.

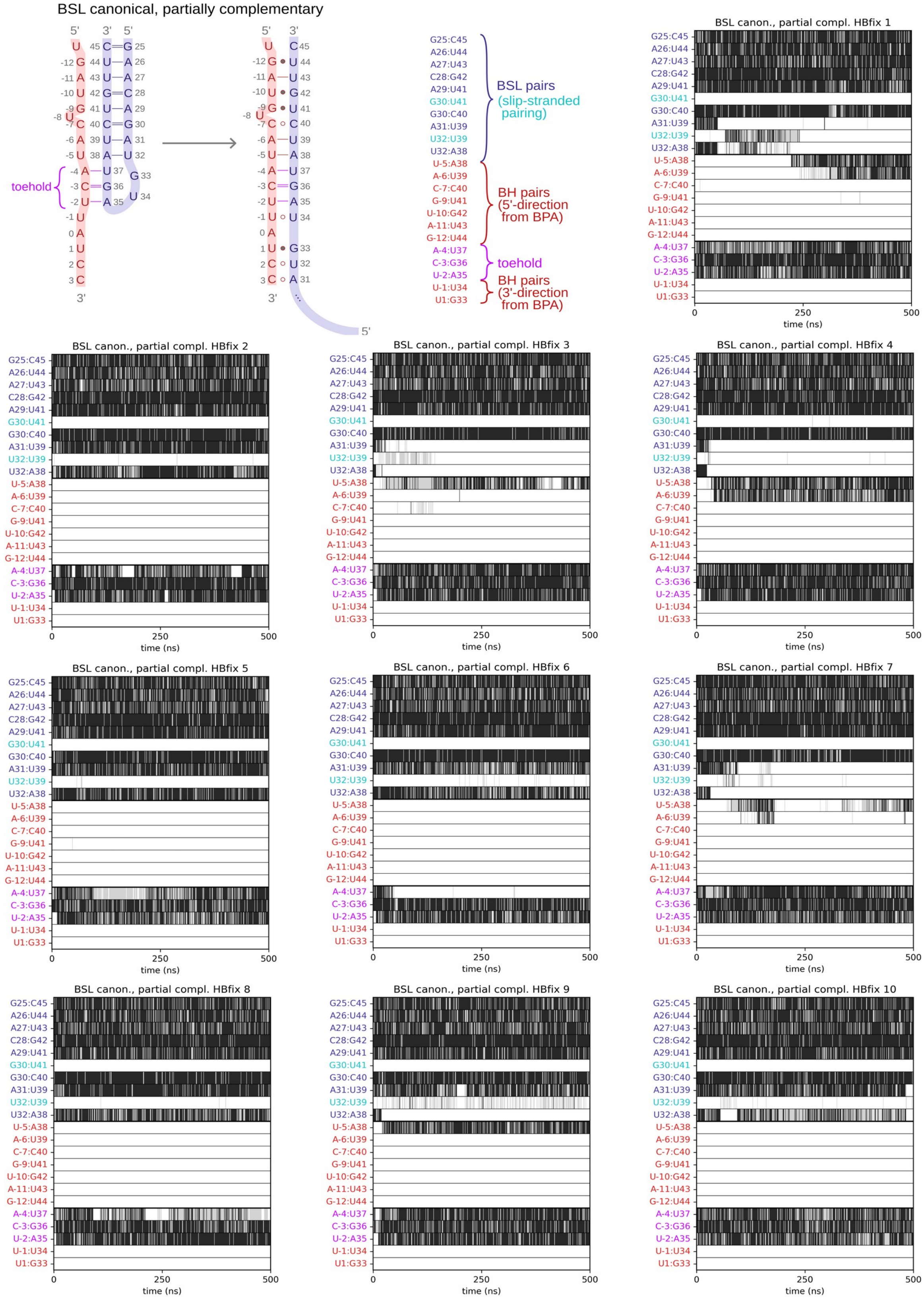


**Figure S10B:** Simulations with the partially complementary intron, idealized canonical BSL, and HB-fix modification. Black, grey, and white colours indicate the full formation of the base pair (heavy-atom distance < 3.2 Å and angle > 140° for all expected H-bonds), weak pairing (distance < 3.5 Å and angle > 120° for at least one of the expected H-bonds), and the absence of base pairing. The pairs of BSL are labelled in blue, while the hydrogen-bonds of the branch helix are labelled in red, the slip-stranded pairs are cyan and the toehold pairs are violet. A scheme of the simulated system is shown in the upper left corners.

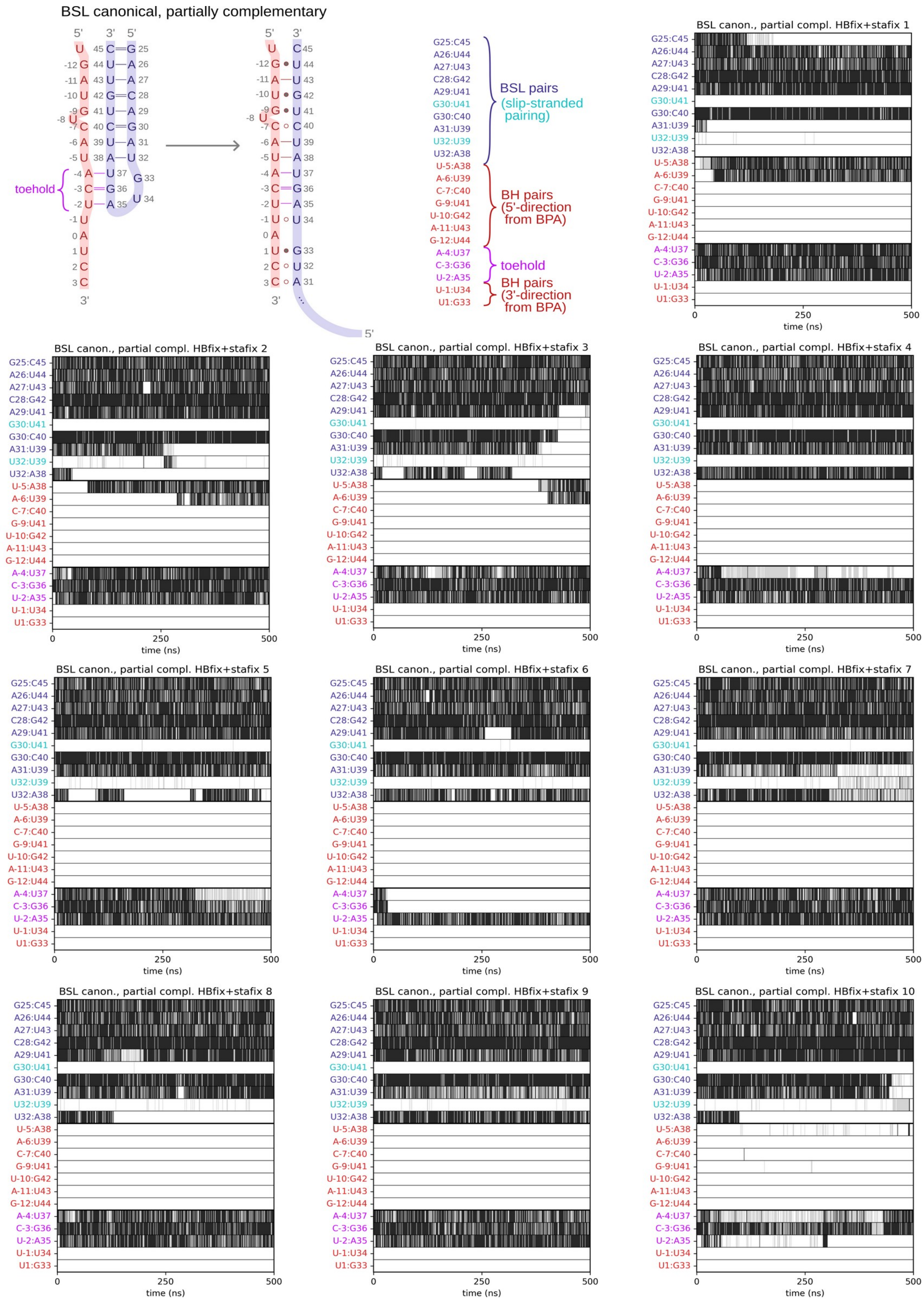


**Figure S10C:** Simulations with the partially complementary intron, idealized canonical BSL, and HB-fix+sta-fix modifications. Black, grey, and white colours indicate the full formation of the base pair (heavy-atom distance < 3.2 Å and angle > 140° for all expected H-bonds), weak pairing (distance < 3.5 Å and angle > 120° for at least one of the expected H-bonds), and the absence of base pairing. The pairs of BSL are labelled in blue, while the hydrogen-bonds of the branch helix are labelled in red, the slip-stranded pairs are cyan and the toehold pairs are violet. A scheme of the simulated system is shown in the upper left corners.

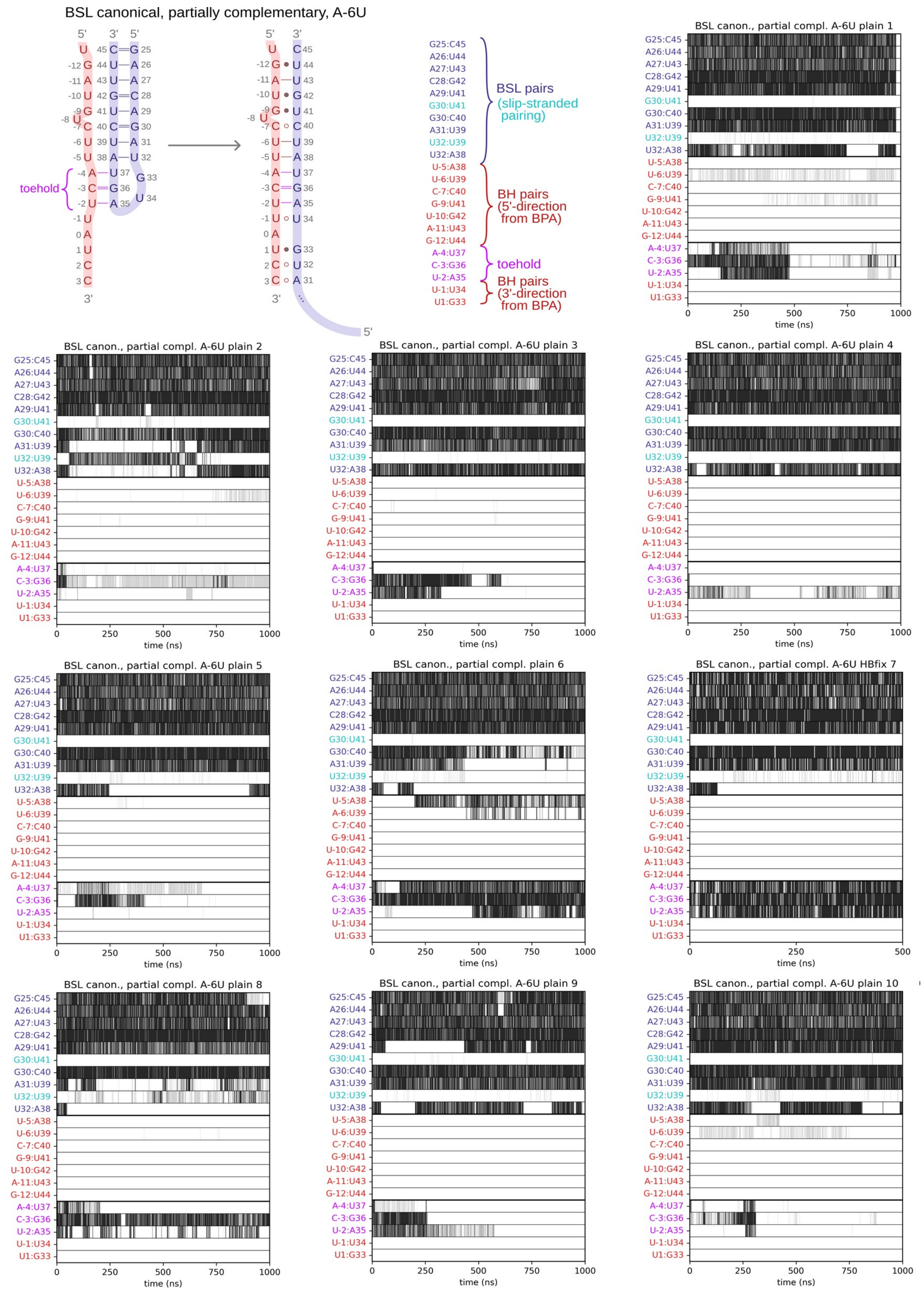


**Figure S11A:** Simulations with the partially complementary intron with A−6U mutation, idealized canonical BSL, and no force field modification. Black, grey, and white colours indicate the full formation of the base pair (heavy-atom distance < 3.2 Å and angle > 140° for all expected H-bonds), weak pairing (distance < 3.5 Å and angle > 120° for at least one of the expected H-bonds), and the absence of base pairing. The pairs of BSL are labelled in blue, while the hydrogen-bonds of the branch helix are labelled in red, the slip-stranded pairs are cyan and the toehold pairs are violet. A scheme of the simulated system is shown in the upper left corners.

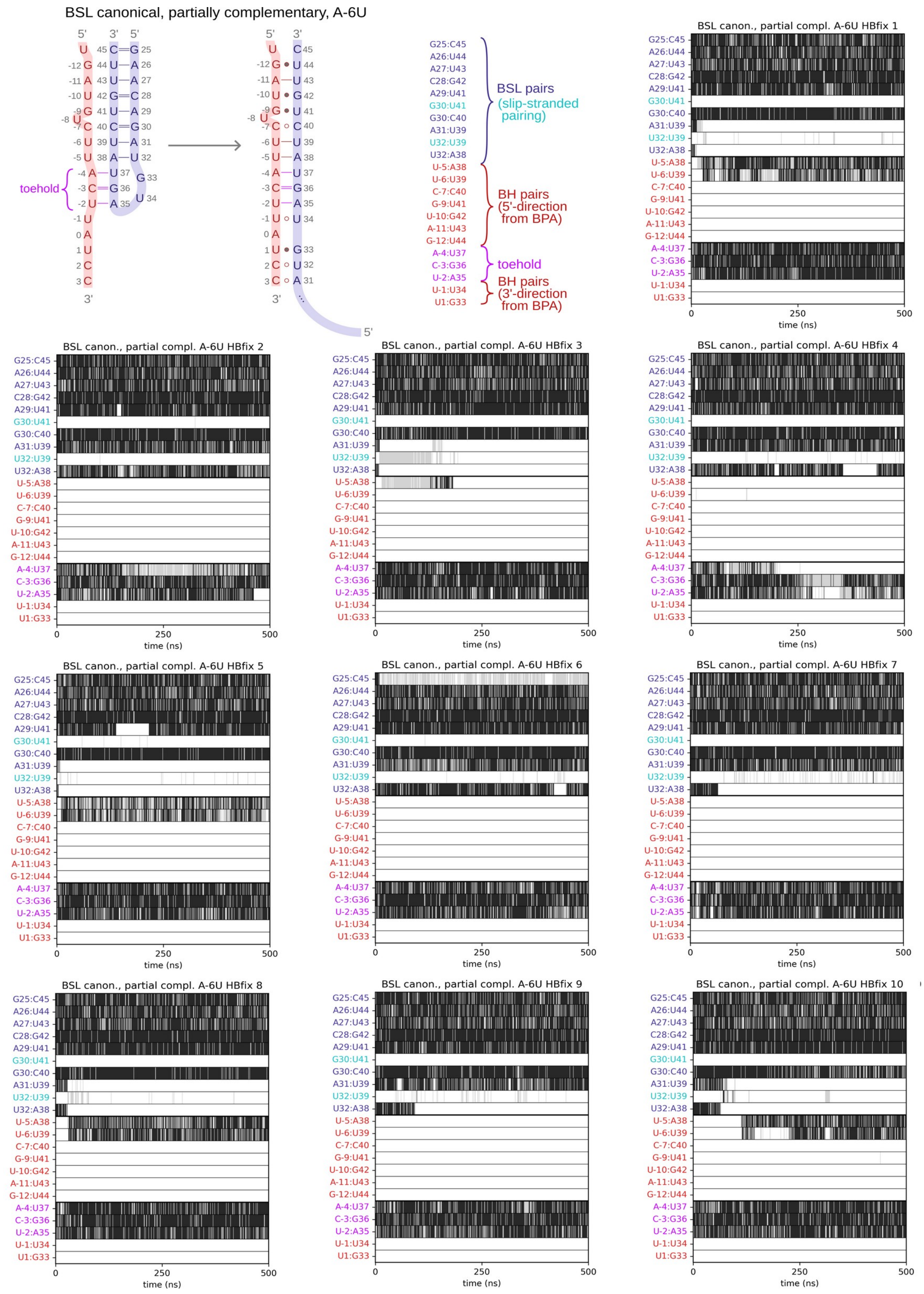


**Figure S11B:** Simulations with the partially complementary intron with A−6U mutation, idealized canonical BSL, and HB-fix modification. Black, grey, and white colours indicate the full formation of the base pair (heavy-atom distance < 3.2 Å and angle > 140° for all expected H-bonds), weak pairing (distance < 3.5 Å and angle > 120° for at least one of the expected H-bonds), and the absence of base pairing. The pairs of BSL are labelled in blue, while the hydrogen-bonds of the branch helix are labelled in red, the slip-stranded pairs are cyan and the toehold pairs are violet. A scheme of the simulated system is shown in the upper left corners.

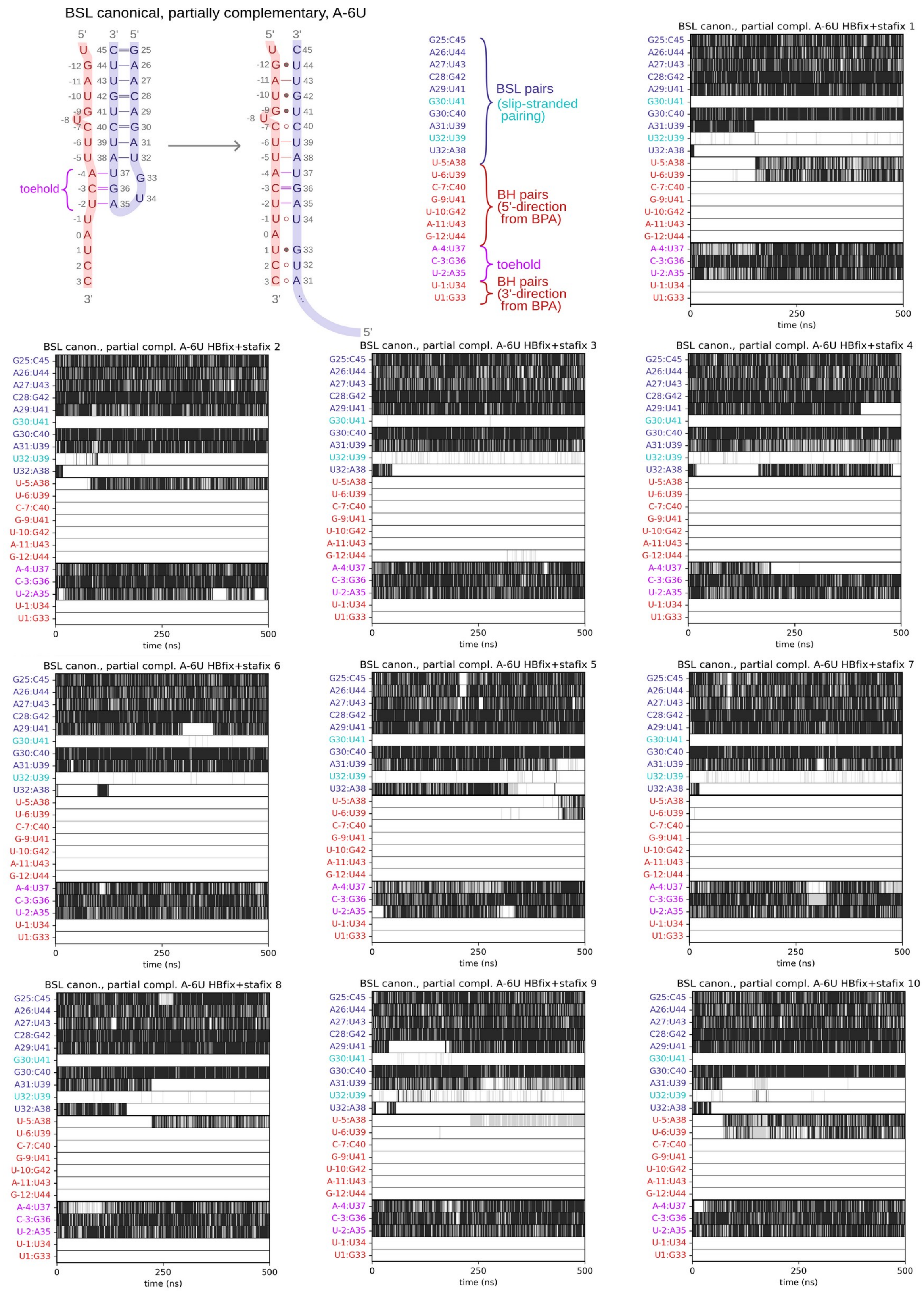


**Figure S11C:** Simulations with the partially complementary intron with A−6U mutation, idealized canonical BSL, and HB-fix+sta-fix modifications. Black, grey, and white colours indicate the full formation of the base pair (heavy-atom distance < 3.2 Å and angle > 140° for all expected H-bonds), weak pairing (distance < 3.5 Å and angle > 120° for at least one of the expected H-bonds), and the absence of base pairing. The pairs of BSL are labelled in blue, while the hydrogen-bonds of the branch helix are labelled in red, the slip-stranded pairs are cyan and the toehold pairs are violet. A scheme of the simulated system is shown in the upper left corners.

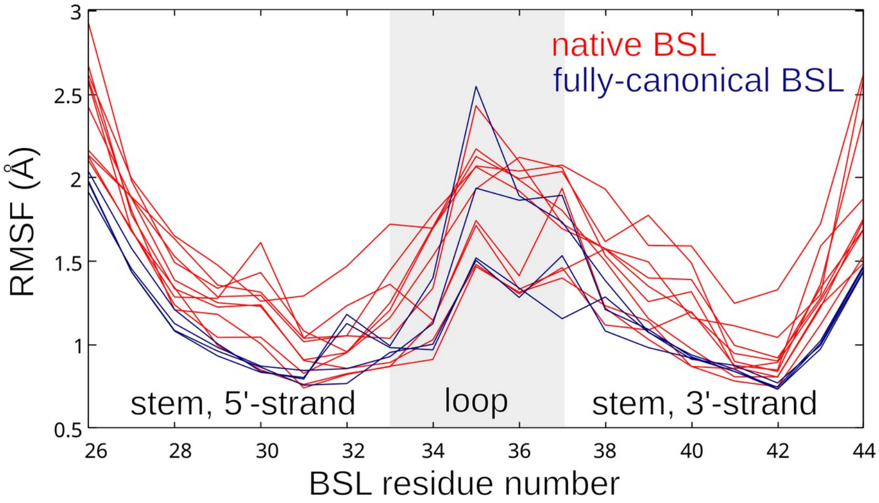


**Figure S12: Per-residue RMSF (Å) of the BSL.** Each line represents an individual trajectory. Only trajectories without force-field modifications that did not sample BSL unwinding are included. Trajectories with the native BSL are taken from simulations of the RNA-only construct with both partially and fully complementary introns.

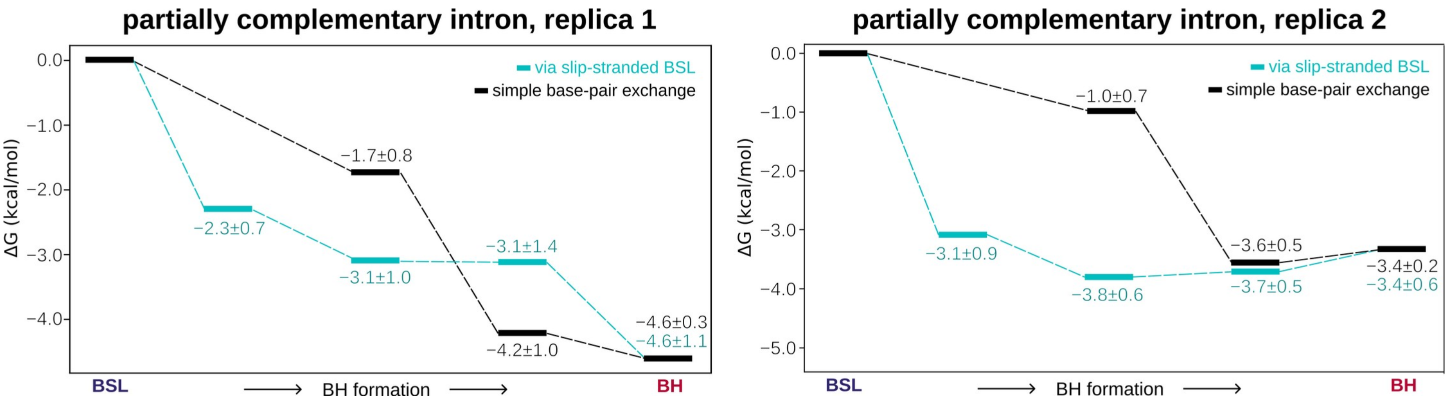


**Figure S13: Scheme of the relative free energy (kcal/mol) of the two competitive pathways (slip-stranded and base-pair exchange pathways) leading to the formation of the branch helix (BH).** The errors for the energy difference between the consecutive steps are reported. The formation of the slip-stranded state is the driving force for the slip-stranded pathway (show in cyan), while the driving force for the base-pair exchange pathway (shown in black) is the formation of two new branch-helix base pairs.

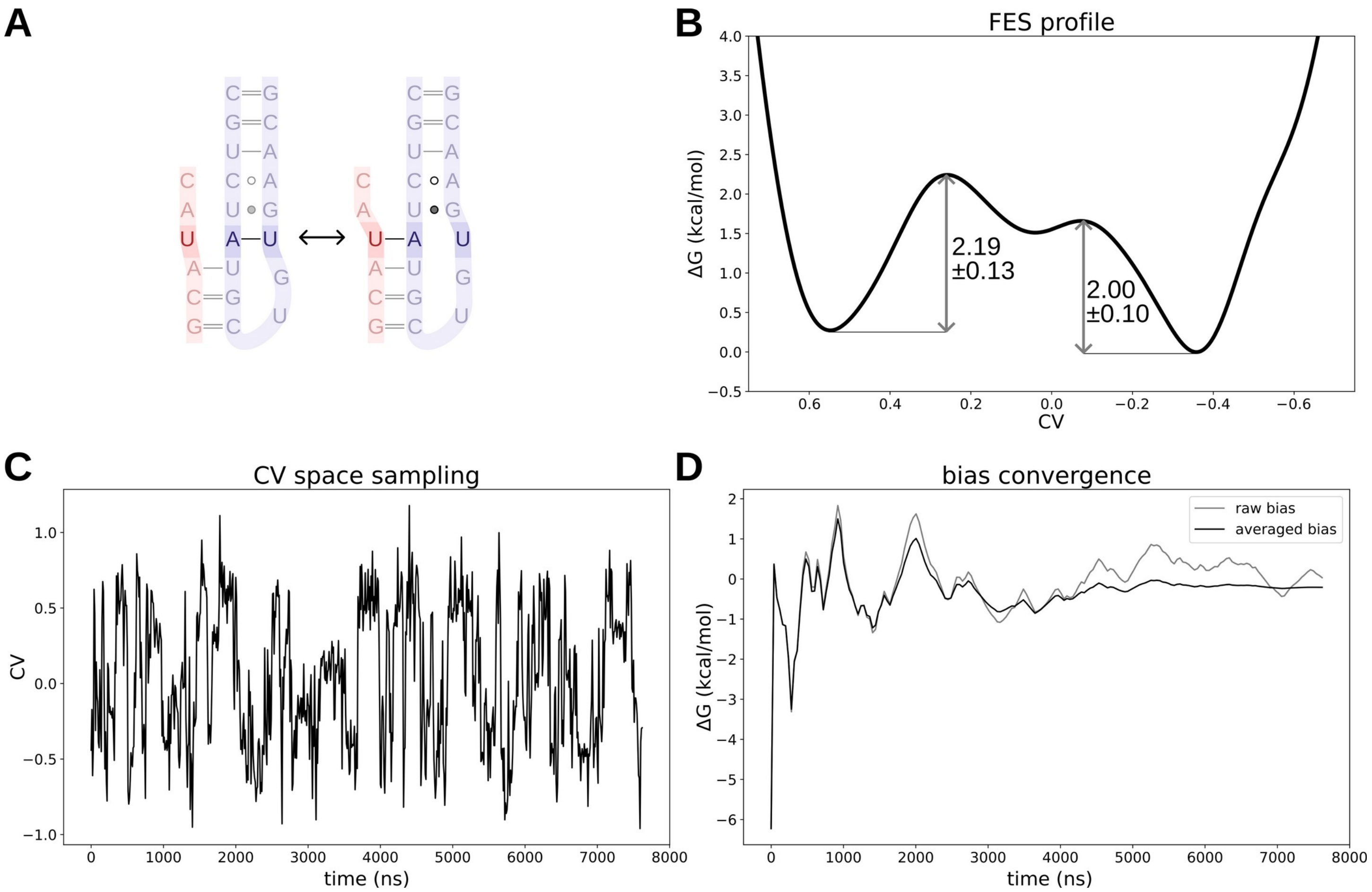


**Figure S14: Supplementary metadynamics simulation of a single base-pair exchange.** This simulation was conducted to complement the qualitative barrier height estimates performed for the metadynamics simulations reported in the main text. **(A)** The construct used and the two target states. **(B)** The free energy profile along the CV. The CV was defined as the difference in the distances of the A(N1)-U(H3) atoms of the two target AU pairs; specifically, as A(N1)-U(H3)$_{BSL}$ – A(N1)-U(H3)$_{BH}$. **(C)** Sampling of the distance CV along the trajectory. **(D)** Convergence of the bias.

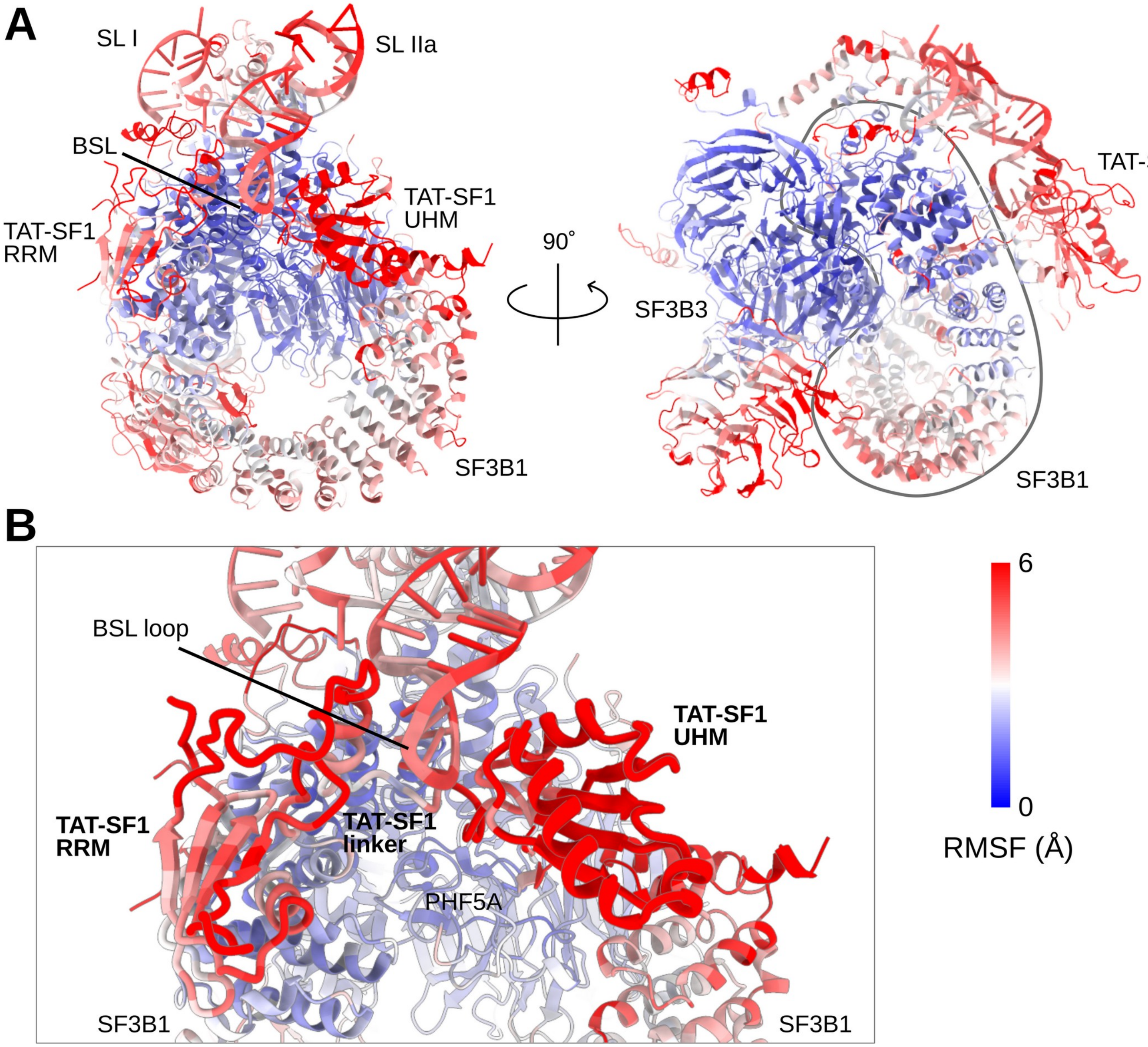


**Figure S15: Root-mean-square fluctuations (RMSF, Å) calculated over the MD simulation trajectories of the U2/SF3b particle with TAT-SF1.** Trajectories of two replicas were collectively analysed. **(A)** Overall model system in front and side views. SF3B1 is highlighted by an outline in the right-hand figure. **(B)** Close-up view of the BSL loop and TAT-SF1 region. TAT-SF1 is highlighted by a thicker cartoon than the rest of the proteins. Colours range from blue to red for low to high RMSF values.

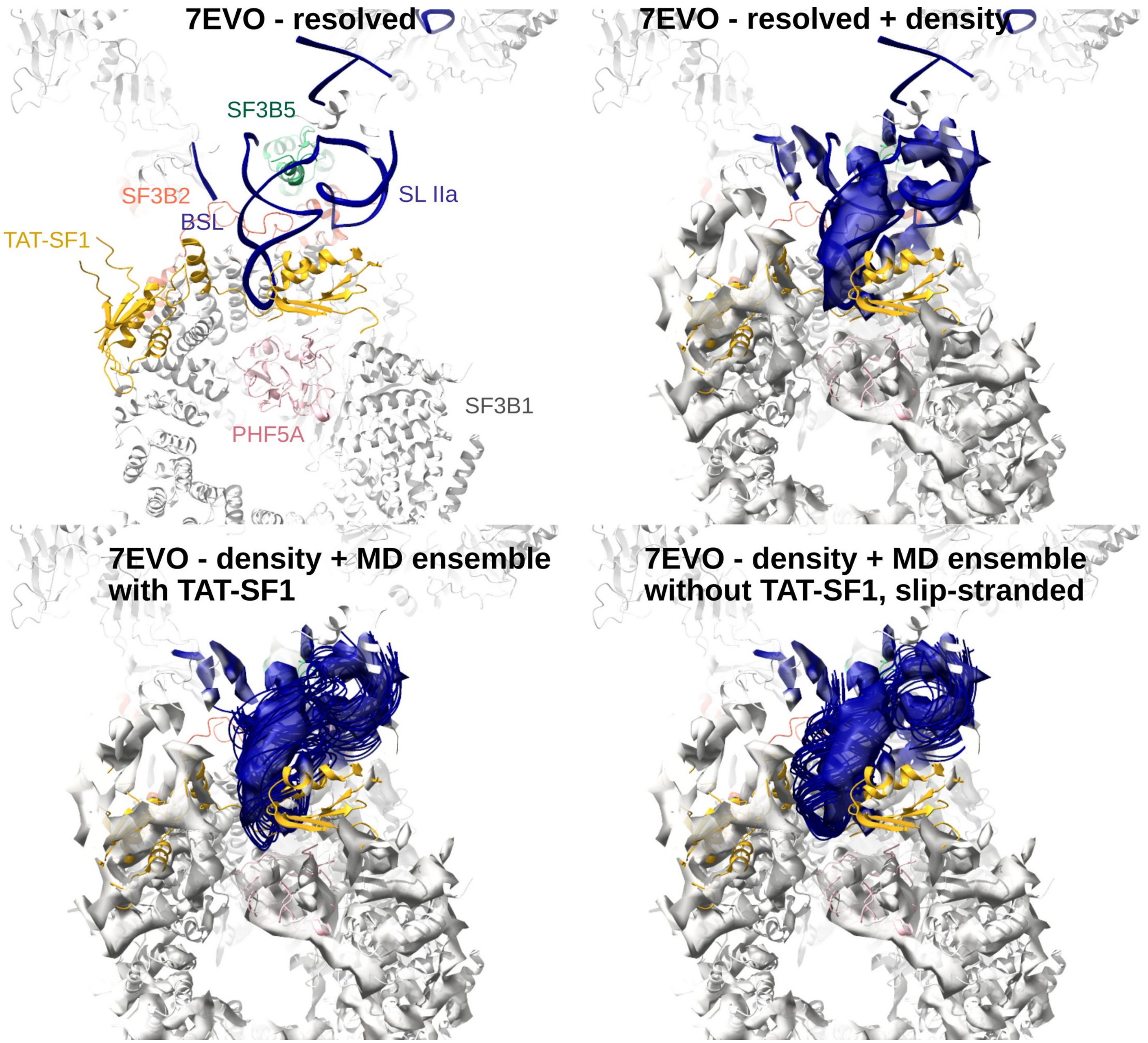


**Figure S16A: Superposition of MD simulation ensembles onto the electron density maps of the 17S U2 snRNP structure available under PDB ID 7EVO.** Twenty simulation snapshots, taken equidistantly along the MD trajectory, are shown to represent the MD ensembles. The snapshots were aligned to the structure using SF3B1 HEAT-domain Cα atoms as a reference, which fluctuate less than the BSL or TAT-SF1 (Figure S15). See also Figures S16B and S16C below.

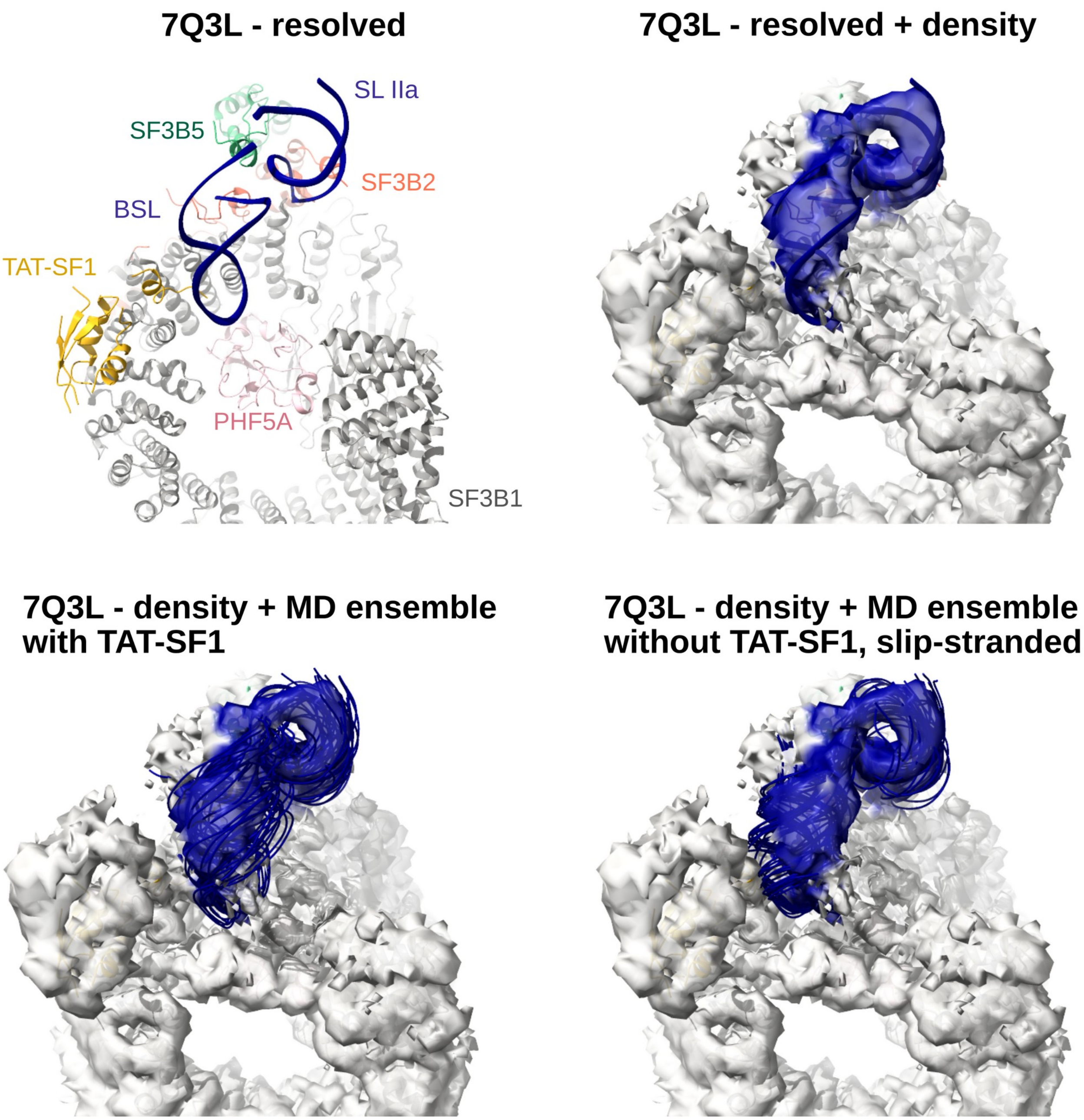


**Figure S16B: Superposition of MD simulation ensembles onto the electron density maps of the 17S U2 snRNP structure available under PDB ID 7Q3L.** Twenty simulation snapshots taken equidistantly along the MD trajectory are shown to represent the MD ensembles. The simulation snapshots were aligned to the structure using SF3B1 HEAT-domain Cα atoms as a reference, which fluctuate less than the BSL or TAT-SF1 (Figure S15). See also Figure S16C below.

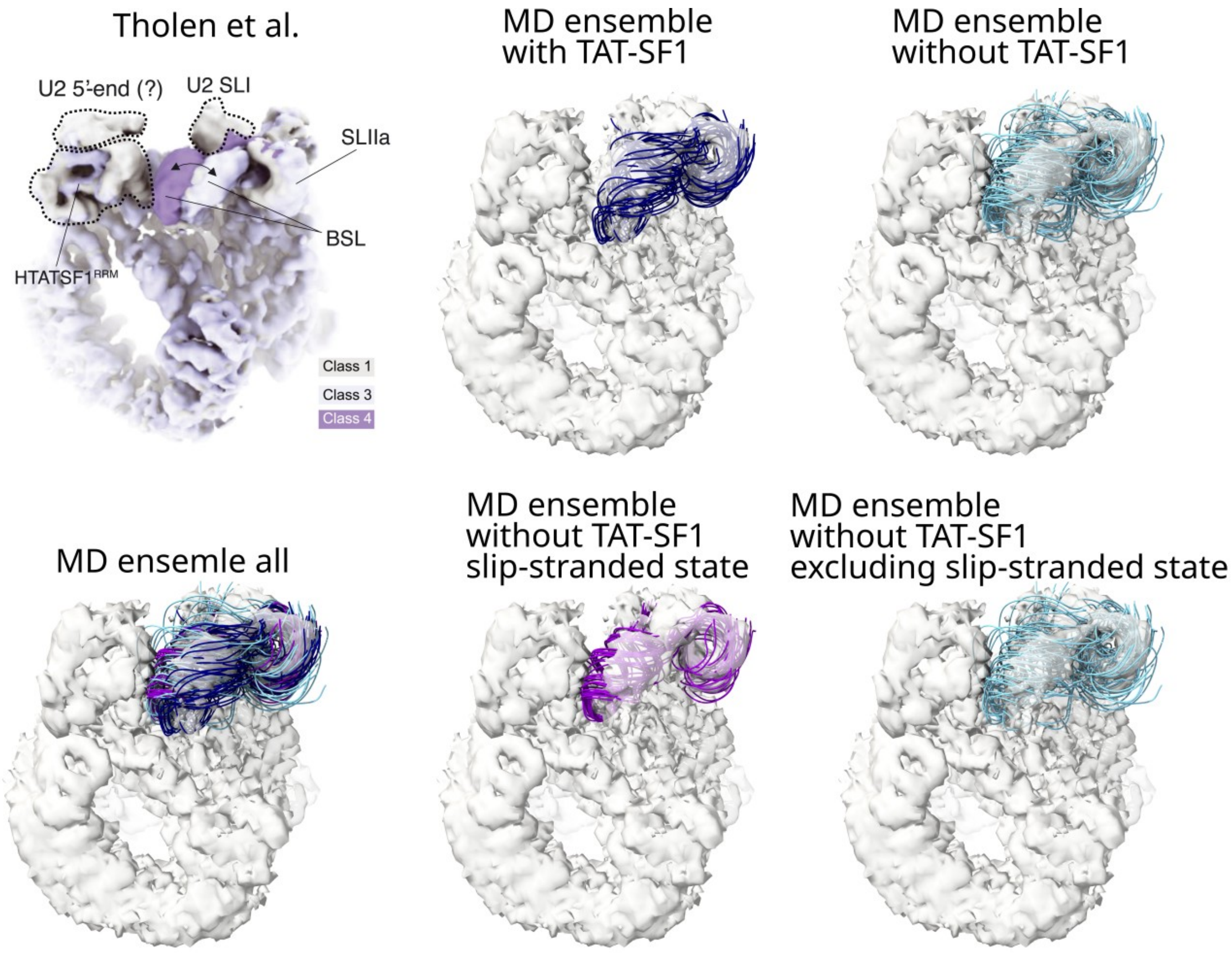


**Fig S16C: BSL conformation in the U2/SF3b complex.** The upper-left corner shows the sub-states identified in Ref. [2]; reproduced from Tholen et al., Science, 10.1126/science.abm4245 2022, AAAS. The remaining figures show different ensembles of BSL and SLIIa taken from MD simulations within the electron density resolved by Tholen et al. (see also Figure S16B). The structures were aligned using SF3B1 Cα atoms as a reference.

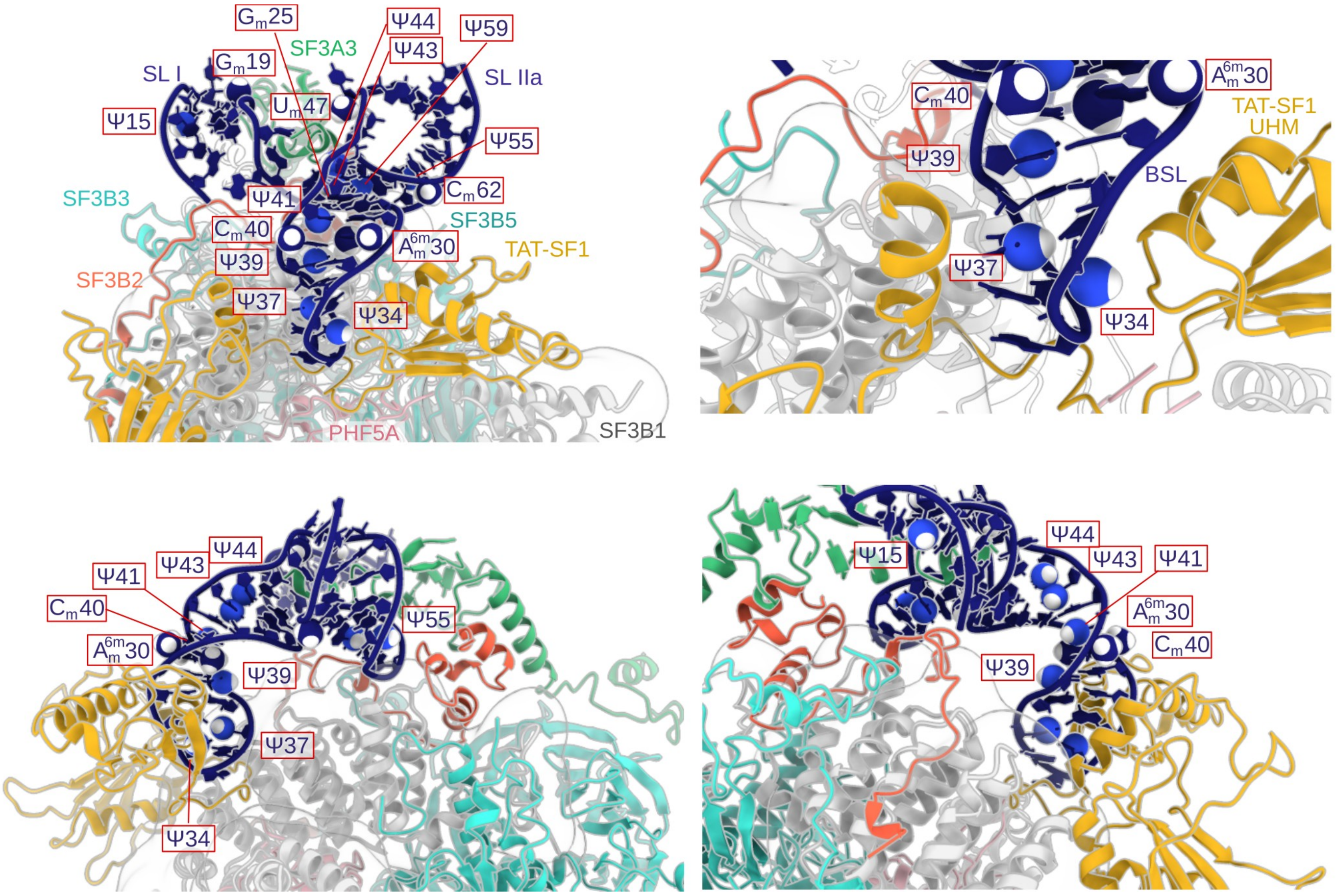


**Figure S17: Locations of U2 snRNA modifications in the 3D structure.** The modified groups are highlighted with spheres. The structural model is built based on the structure deposited as PDB ID 7EVO.

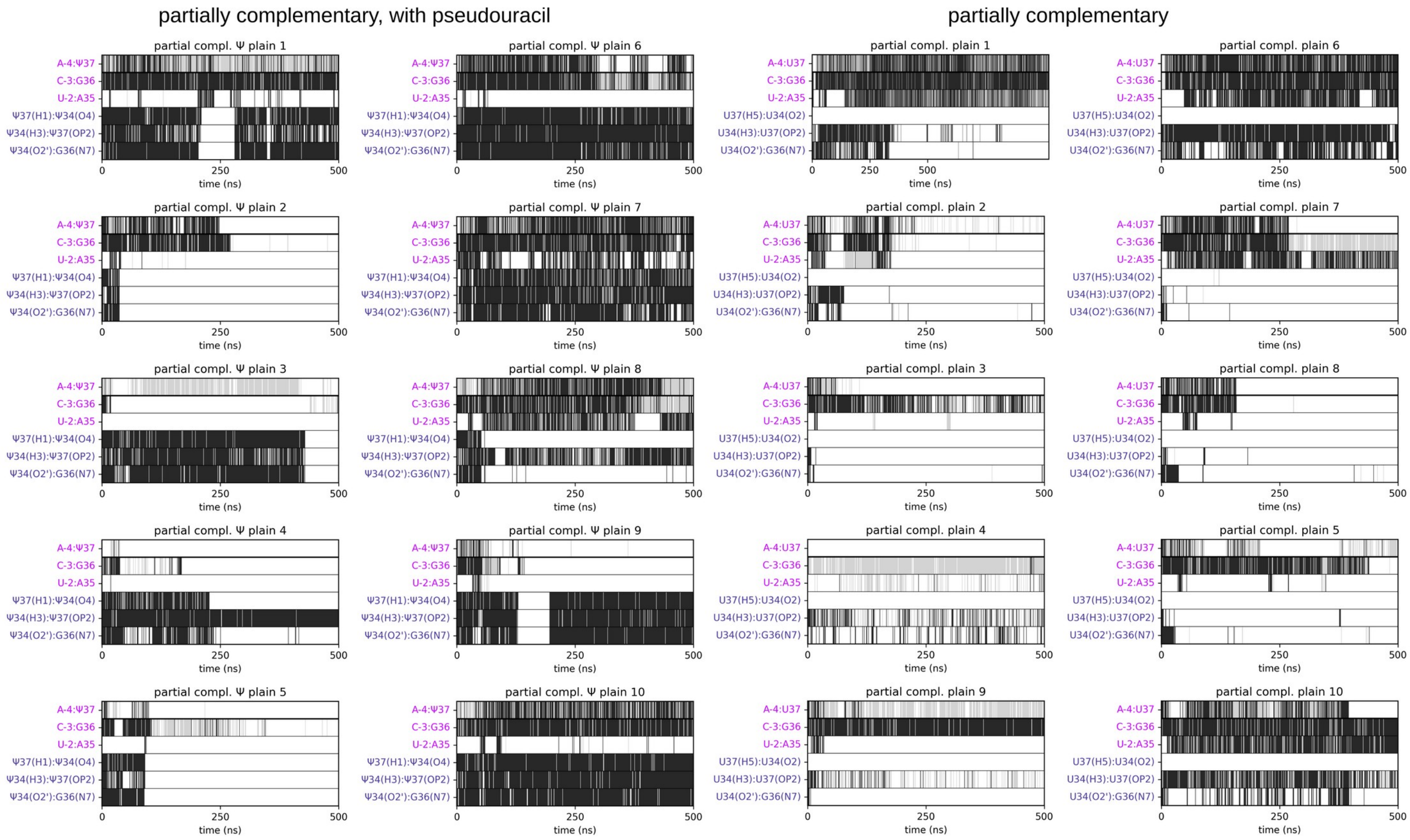


**Figure S18: Base-pairing and H-bond development for the toehold (violet-labelled) and the BSL loop (dark-blue-labelled) in MD simulations of the intron-BSL construct with and without pseudouridines.** No force-field modifications were used in these simulations. Black, grey, and white colours indicate the full formation of the base pair (heavy-atom distance < 3.2 Å and angle > 140° for all expected H-bonds), weak pairing (distance < 3.5 Å and angle > 120° for at least one of the expected H-bonds), and the absence of base pairing. The pairs of the BSL are labelled in blue, while the toehold pairs are violet.

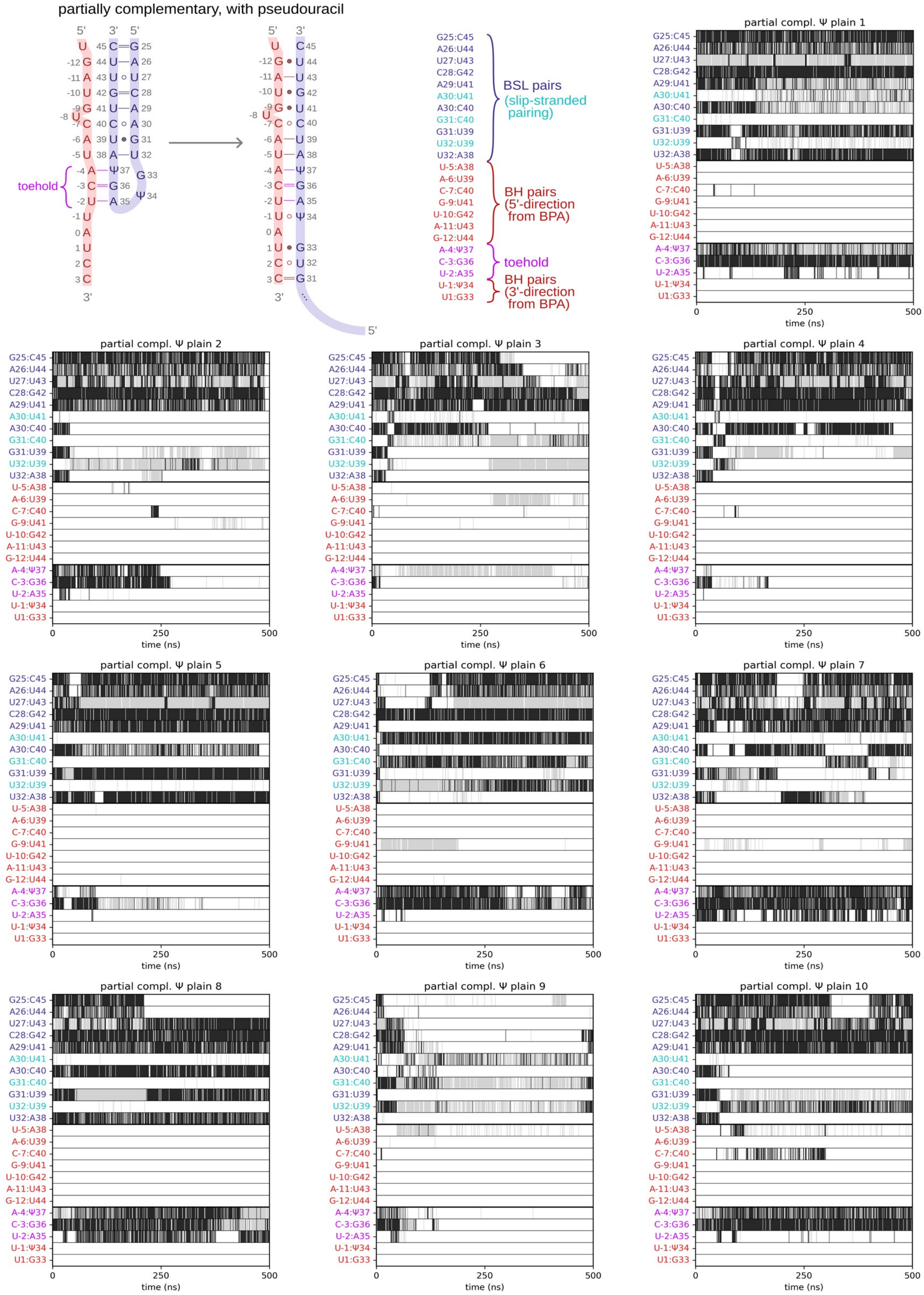


**Figure S19:** Simulations with the partially complementary intron, pseudouridines in BSL loop, and no force field modification. Black, grey, and white colours indicate the full formation of the base pair (heavy-atom distance < 3.2 Å and angle > 140° for all expected H-bonds), weak pairing (distance < 3.5 Å and angle > 120° for at least one of the expected H-bonds), and the absence of base pairing. The pairs of BSL are labelled in blue, while the hydrogen-bonds of the branch helix are labelled in red, the slip-stranded pairs are cyan and the toehold pairs are violet. A scheme of the simulated system is shown in the upper left corners.

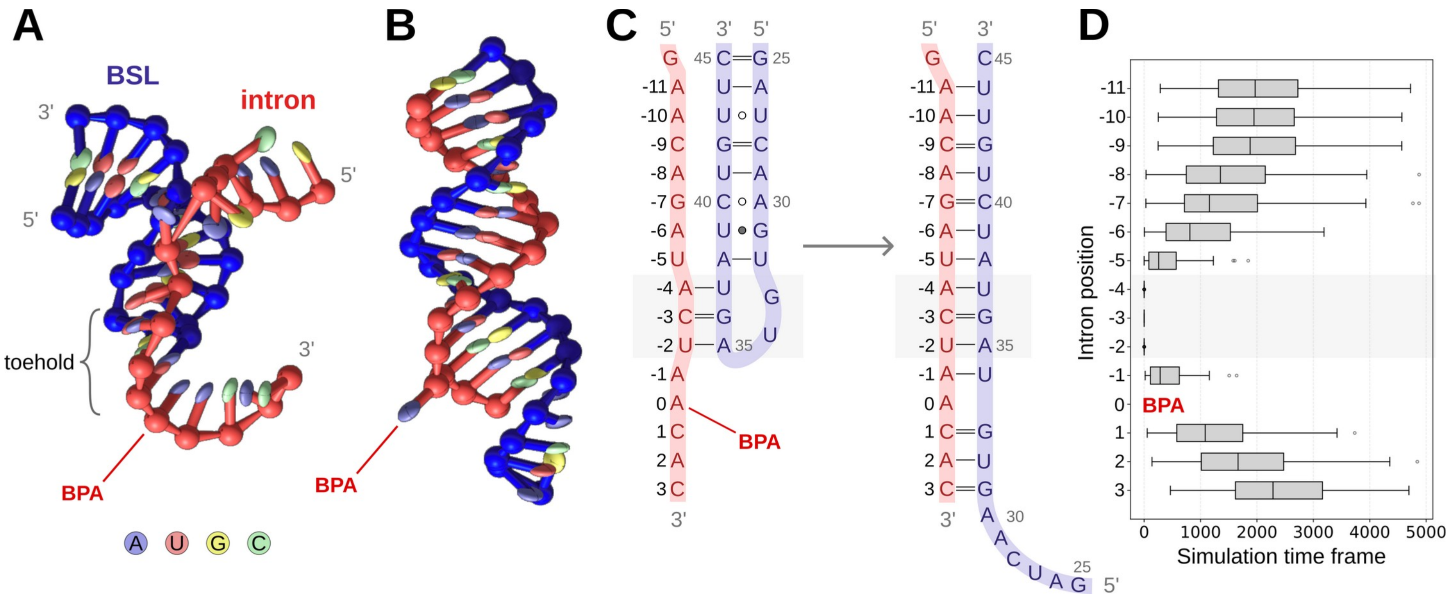


**Figure S20: Branch helix formation in coarse-grained (CG) simulations.** Due to the complexity and the time scale required for this substantial conformational remodelling, we accelerated the sampling by performing CG simulations, witch come at the cost of losing atomic-level details. The chosen oxRNA model accounts only for canonical and G:U wobble base pairs, so we simulated only the intron sequence that is fully complementary to the BSL. CG simulations were started from the same toehold-bound structure used in the all-atom MD simulations **(A)**; **(B)** shows the final structure. In the selected snapshot of the final structure, the BPA is bulged out. **(C)** Schematic representation of **(A)** and **(B)**. The toehold area is marked by a grey box. **(D)** Statistics on the branch helix base pair formation vs. simulation time frame. The average is collected over 100 CG trajectories. In 10 trajectories, the branch helix formed only partially, and in 7 trajectories, the toehold was lost and the two strands dissociated. Additionally, in 30% of the trajectories, the formation of transient BPA bulge-out events was observed. During the growth of the branch helix, the BPA remained intra-helical, being bulged out only after complete formation of the branch helix (Fig. S21). In the intra-helical state, the BPA was mostly without a pairing partner but occasionally paired with U34 or G33 (Fig. S22).

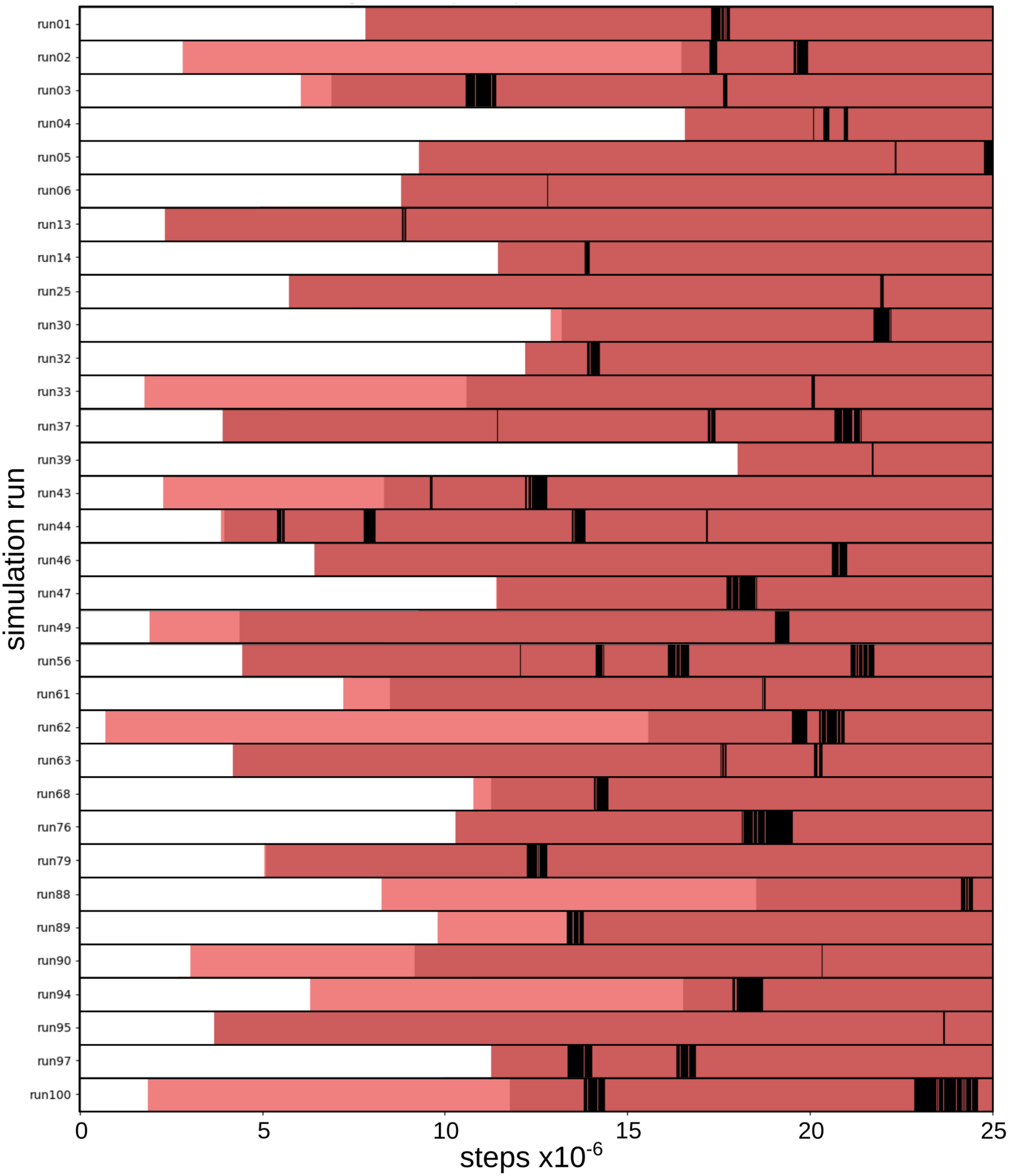


**Figure S21: Branch point adenosine (BPA) bulge-out events versus simulation steps in coarse-grained simulations.** Only the trajectories that sampled the BPA bulge-out events are shown. In the histogram, light red marks the formation of the penultimate base pair of the branch helix end flanking the BPA (A3:U32), dark red marks the formation of the last pair (C4:G31), and the black areas mark the BPA bulge-out events.

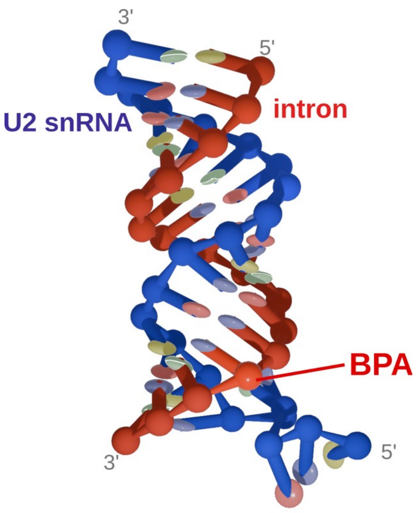


**Figure S22: Snapshot of the CG simulation showing an intra-helical unpaired BPA.**

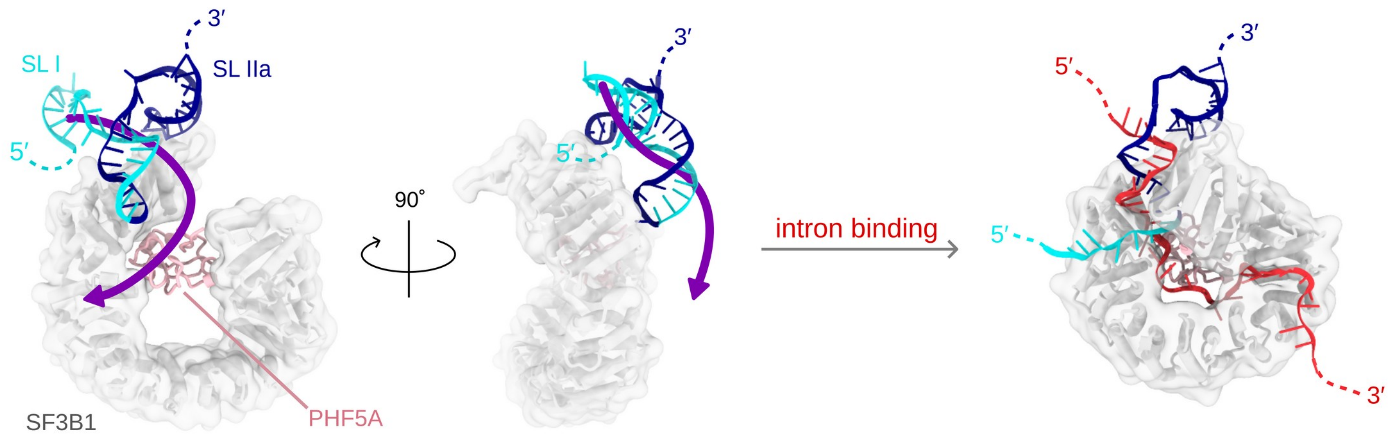


**Figure S23: Relocation of the U2 snRNA 5’-end (in light blue) necessary to create space for intron binding and formation of the branch helix.** The putative relocation direction is indicated by a violet arrow.

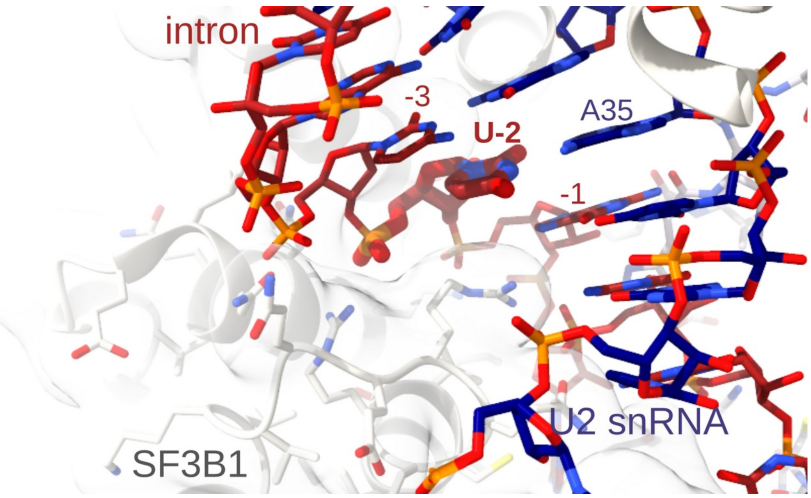


**Figure S24: U−2 docking onto the SF3B1 surface as captured by the structure deposited in PDB ID 6FF4.** Any nucleotide different from U bound to A35 would alter the shape of the backbone, thus deviating from the A-RNA helix geometry.

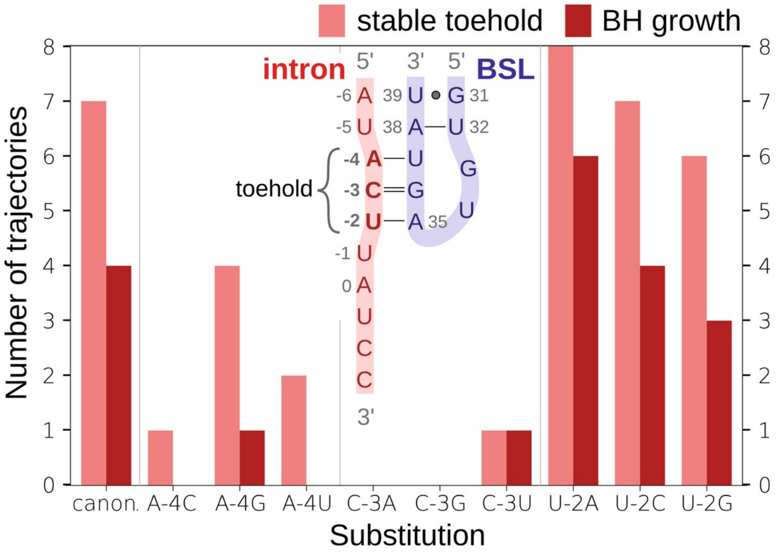


**Figure S25: MD simulations of branch helix growth containing substitutions in the toehold-binding bases of the intron.** Only a small RNA construct was used in these simulations, as depicted in the scheme. The substituted systems were prepared using tleap and carefully equilibrated in two rounds: the first round with all residues that do not participate in the substituted base pair restrained, followed by a second round of standard equilibration. For each sequence variant, 10 MD simulation replicas were run, each for 500 ns. The resulting trajectories were analysed for toehold stability and the growth of the branch helix. A base-pair was considered fully paired (stably-formed) if, for at least 50% of the simulation time and, at the same time, in the last 20 ns of the trajectory, the following geometric criteria were satisfied: heavy-atom distance < 3.25 Å and angle > 140° for all base-pair H-bonds. These criteria were chosen after careful visual analysis of the trajectory evolution. The toehold was considered stable if these criteria were satisfied for all non-substituted base pairs (e.g., all three toehold base pairs for the 'canonical' sequence, while only the pairs involving the −2 and −3 positions for the cases with substitution at the −4 position). The branch helix growth was analysed by applying these criteria to the A−6:U39 pair. The results indicate that the middle (−3) position is more critical than position −4. The −2 position seems to have no dramatic effect. We note, however, that the construct used is very small and only captures local effects, thus likely underestimating the role of the −2 position (see also Fig. S24).

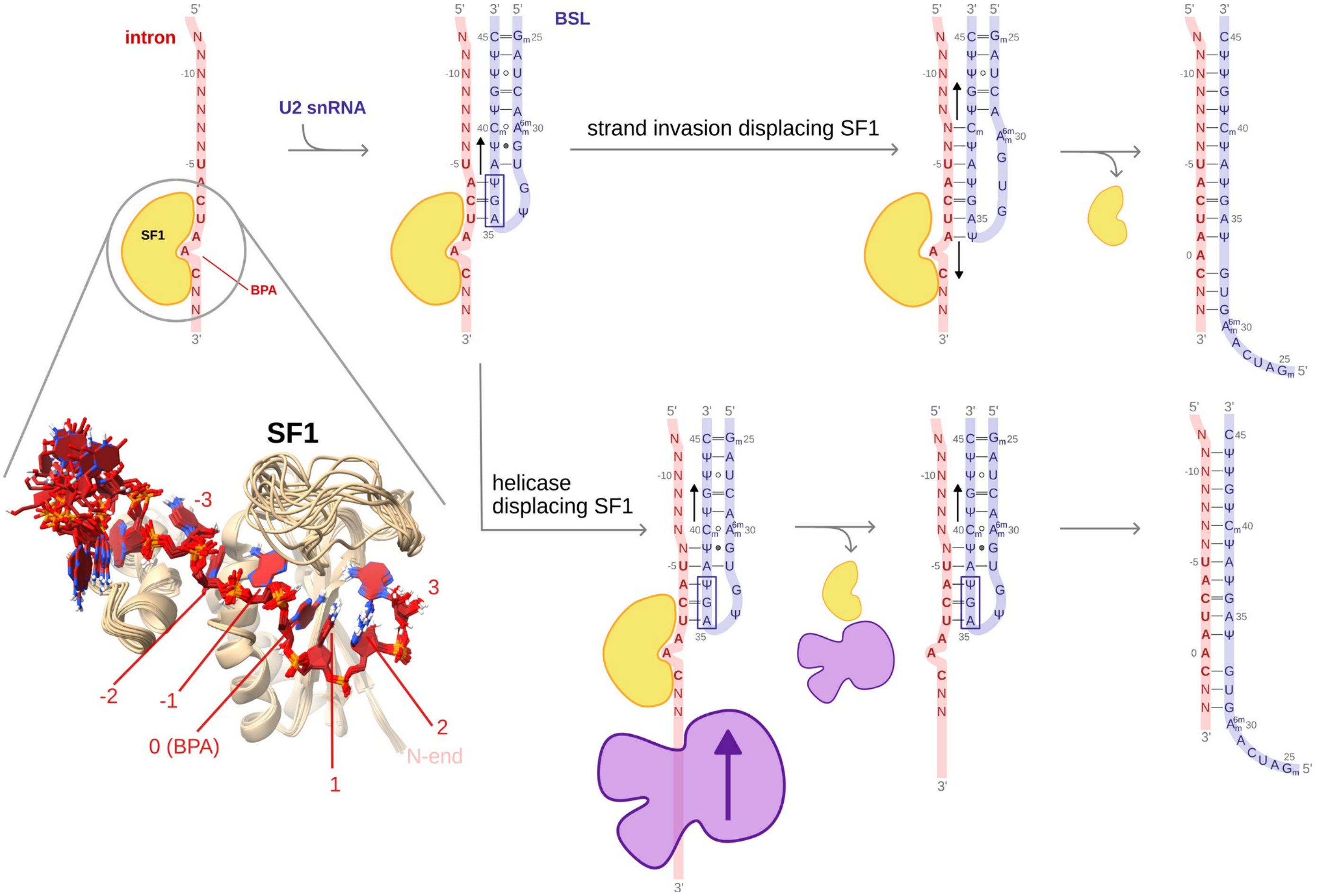


**Fig S26: Possible involvement of SF1 (yellow) and a putative mechanism of SF1 displacement from the intron.** The inset shows an NMR ensemble of RNA binding to SF1. The SF1 binding motif is nearly identical to the consensus branch site motif, differing only in a higher preference for U and A at positions −5 and −4, respectively, and a slightly higher degeneracy for the rest of the sequence [3,4]. The figure was prepared using structure PDB ID 1K1G. Before binding to U2 snRNA, the branch site is cooperatively localized by SF1 and U2AF, which form a platform via direct contacts with the branch point adenosine, the adjacent PPT and 3' splice site, and hand it over to U2 snRNP. Nevertheless, the exact mechanism by which the intron is delivered to U2 snRNP remains unclear. When bound to SF1, the BPA is buried in a protein pocket, while other bases remain solvent-exposed (see inset) [5]. This aligns with the fact that U2 snRNP can bind the toehold-pairing motif of the intron (at positions −4 to −2) before binding the BPA (Fig. 1D' in the main text) [6]. It is thus plausible to assume that SF1 aids intron-U2 snRNA binding not only by fixing the BPA in a "pre-bulged" state [5], but also by juxtaposing the intron toehold-pairing signature to the BSL. SF1 could then be stripped away from the intron by a push/pull from PRP43 (DHX15, violet in the scheme), the other RNA helicase acting in this step of the splicing cycle (bottom row). In this scenario, only a strong initial short branch helix could withstand the action of PRP43, while a weak branch helix could be dissociated. Alternatively, the strand-invasion process itself may be responsible for displacing SF1 (top row), if the toehold-intron pairing is strong enough to support the helix growth. Nevertheless, the contribution of SF1/U2AF is more likely to be that of facilitating rather than strictly enforcing it, due to the the high degree of intrinsic disorder in SF1/U2AF and the fact that SF1 is dispensable for the splicing of strong branch sequences [3,5]. One interesting

hypothesis is that SF1 and U2AF orient U2 snRNP within a probabilistic spatial window that favours BSL interaction, while still leaving flexibility for the BSL to scan nearby branch-site positions [7], and engage on the the most favourable one through strand invasion. The driving force for the BSL scanning might be provided by PRP43, which, unlike PRP5, is a processive translocase gliding on/pulling the RNA strand in the 3′-to-5′ (upstream) direction. PRP43 impairment indeed leads to promiscuous branch site selection and the usage of cryptic branch sites upstream of the canonical ones [8]. Another line of evidence supporting this hypothesis is that branch site antagonists (like pladienolides or spliceostatins) induce the selection of alternative “stronger” (more conserved) branch sequences upstream of the typically engaged one [6,9]. Thus, the BSL—potentially driven by PRP43—can glide along the intron through continuous base-pair exchange until it becomes stabilized at a preferred branch site [7].

# Supplementary References

1. Crean RM, Slusky JSG, Kasson PM and Kamerlin SCL. KIF-Key Interactions Finder: A program to identify the key molecular interactions that regulate protein conformational changes. *J. Chem. Phys.* 2023; **158**: 144114.
2. Tholen J, Razew M, Weis F and Galej WP. Structural basis of branch site recognition by the human spliceosome. *Science*. 2022; **375**: 50–57.
3. Corioni M, Antih N, Tanackovic G, Zavolan M and Krämer A. Analysis of in situ pre-mRNA targets of human splicing factor SF1 reveals a function in alternative splicing. *Nucleic Acids Res.* 2011; **39**: 1868–1879.
4. Pastuszak AW, Joachimiak MP, Blanchette M, Rio DC, Brenner SE and Frankel AD. An SF1 affinity model to identify branch point sequences in human introns. *Nucleic Acids Res.* 2011; **39**: 2344–2356.
5. Liu Z, Luyten I, Bottomley MJ, Messias AC, Houngninou-Molango S, Sprangers R, Zanier K, Krämer A and Sattler M. Structural Basis for Recognition of the Intron Branch Site RNA by Splicing Factor 1. *Science*. 2001; **294**: 1098–1102.
6. Cretu C, Gee P, Liu X, Agrawal A, Nguyen T-V, Ghosh AK, Cook A, Jurica M, Larsen NA and Pena V. Structural basis of intron selection by U2 snRNP in the presence of covalent inhibitors. *Nat. Commun.* 2021; **12**: 4491.
7. Kao C-Y, Cao E-C, Wai HL and Cheng S-C. Evidence for complex dynamics during U2 snRNP selection of the intron branchpoint. *Nucleic Acids Res.* 2021; **49**: 9965–9977.
8. Zhang J, Huang J, Xu K, Xing P, Huang Y, Liu Z, Tong L and Manley JL. DHX15 is involved in SUGP1-mediated RNA missplicing by mutant SF3B1 in cancer. *Proc. Natl. Acad. Sci.* 2022; **119**: e2216712119.
9. Vigevani L, Gohr A, Webb T, Irimia M and Valcárcel J. Molecular basis of differential 3′ splice site sensitivity to anti-tumor drugs targeting U2 snRNP. *Nat. Commun.* 2017; **8**: 2100.
